# Dinuclear PhosphoiminoBINOL-Pd Container for Malononitrile: Catalytic Asymmetric Double Mannich Reaction for Chiral 1,3-Diamine Synthesis

**DOI:** 10.1038/s41598-018-19178-4

**Published:** 2018-01-16

**Authors:** Takayoshi Arai, Katsuya Sato, Ayu Nakamura, Hiroki Makino, Hyuma Masu

**Affiliations:** 1 Soft Molecular Activation Research Center (SMARC), Graduate School of Science, Chiba University, 1-33 Yayoi, Inage Chiba, 263-8522 Japan; 2 Molecular Chirality Research Center (MCRC), Graduate School of Science, Chiba University, 1-33 Yayoi, Inage Chiba, 263-8522 Japan; 3 Department of Chemistry, Graduate School of Science, Chiba University, 1-33 Yayoi, Inage Chiba, 263-8522 Japan; 4 Center for Analytical Instrumentation, Chiba University, 1-33 Yayoi, Inage Chiba, 263-8522 Japan

## Abstract

A phosphoiminoBINOL ligand was designed to form a dinuclear metal complex that could hold a malononitrile molecule. The dinuclear bis(phosphoimino)binaphthoxy-Pd_2_(OAc)_2_ complex catalyzed a double Mannich reaction of *N*-Boc-imines with malononitrile to give chiral 1,3-diamines with high enantioselectivity. The rational asymmetric catalyst, which smoothly introduces the first coupling product to the second coupling reaction while avoiding the reverse reaction, facilitates the over-reaction into a productive reaction process.

## Introduction

**Figure 1:**
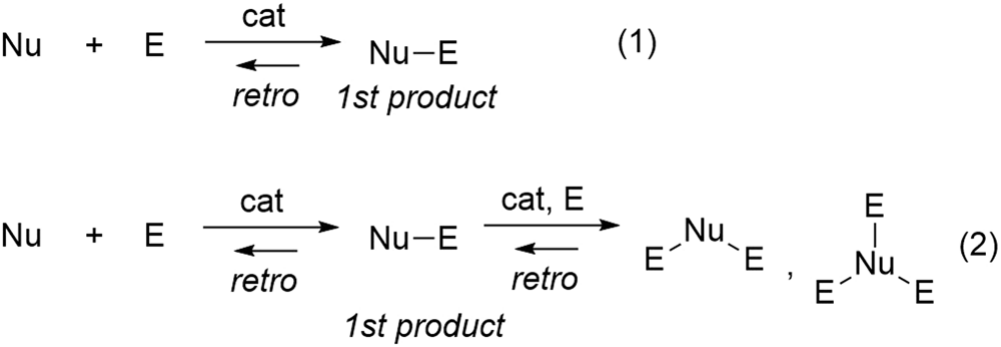
Reaction of a nucleophile (Nu) with an electrophile (E).

The importance of catalysts with high catalytic activity in achieving “green” or sustainable chemistry has been well documented^1^. The benefit of high catalytic activity is not limited to reducing the amount of catalyst used. Catalysts with superior activity have the potential to promote unprecedented chemical transformations. As shown in Fig. 1, in the reaction of a nucleophile (Nu) with an electrophile (E), conventional catalysts are used for the synthesis of the 1:1 coupling adduct Nu-E (eq. 1).

If the first coupling product (Nu − E) can be made to react subsequently with further electrophiles through catalysis, 1:2 and/or 1:3 adducts (i.e. Nu-E_2_, Nu-E_3_) can be obtained. Although reactions of this type can be seen as over-reactions (eq. 2), such multicomponent coupling reactions are fascinating due to their potential as a direct approach toward highly functionalized advanced materials. List *et al*. reported an outstanding example: the condensation of acetaldehyde with two molecules of *N*-Boc imine using 20 mol% proline as a catalyst, which was originally developed for a single condensation reaction^2^. In order to utilize the double condensation reaction as a rational asymmetric catalytic reaction, new concepts for the design of highly active catalysts are required, and the first coupling product must be smoothly introduced to the second coupling reaction while avoiding the reverse reaction. Here, we report the design and development of a dinuclear palladium catalyst that enables a novel double Mannich reaction.

**Figure 2:**
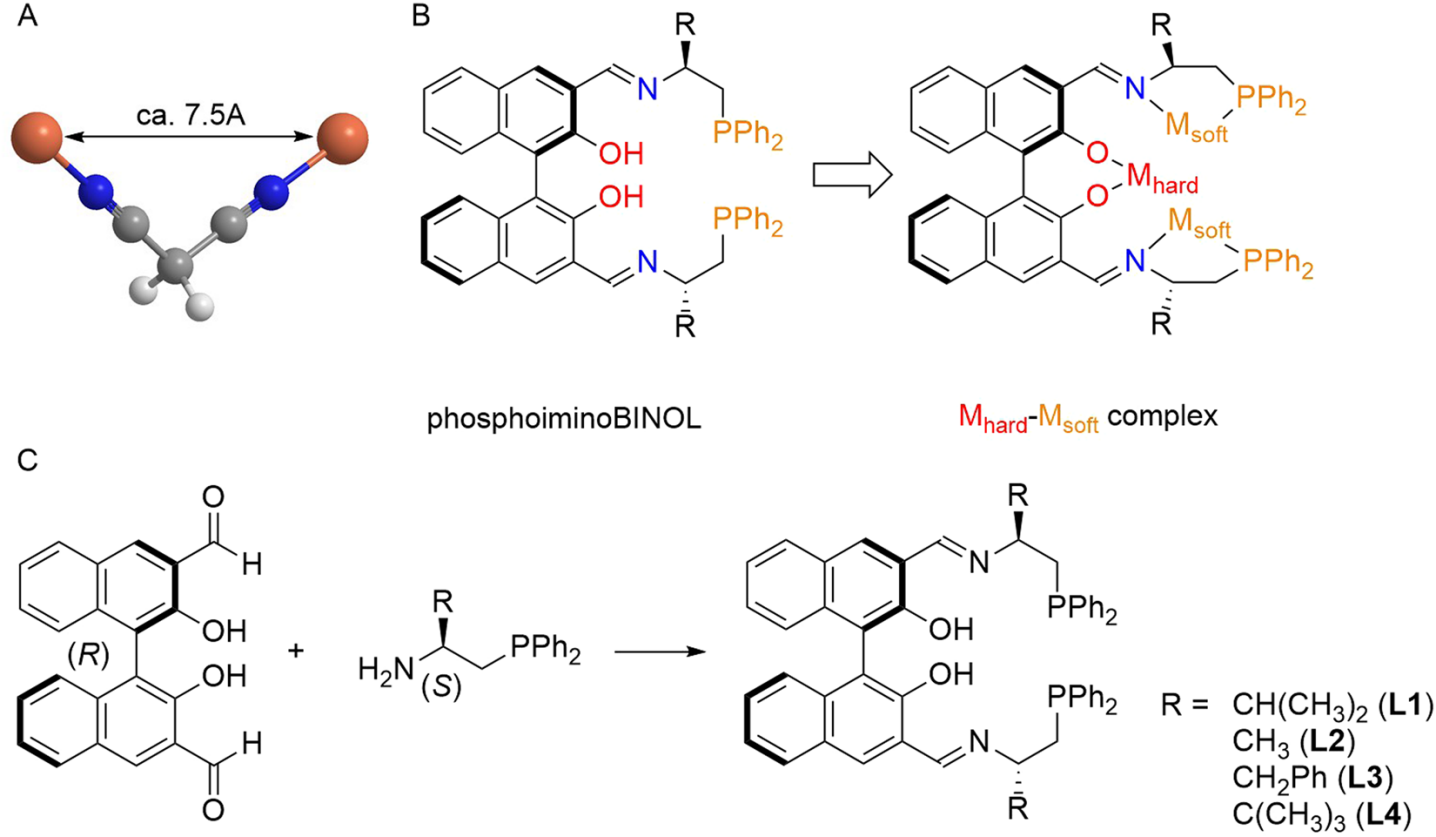
Design of Malononitrile Container. (**A**) Geometry of malononitrile bound to two metals, (**B**) Design of the multifunctional dinuclear phosphoiminoBINOL-metal complex for binding malononitrile. (**C**) Preparation of phosphoiminoBINOL ligands.

In this study, malononitrile was selected as a nucleophile with two acidic protons to be directed toward a double Mannich reaction^3–6^. In the interactions of nitriles with late transition metal salts, malononitrile can bind to two metal atoms^7^. For example, for the 5th-period elements, the two metal centers should be around 7.5 Å from each side of the malononitrile unit (Fig. 2A). In order to achieve a dinuclear reaction in one asymmetric reaction sphere while retaining the same geometry, a novel phosphoiminoBINOL ligand was designed, as shown in Fig. 2B. The phosphoimino moiety is designed to capture soft metals such as Pd, Rh, Au, Ag, and Cu, and the soft dinuclear complex binds strongly to malononitrile (or an anion of malononitrile generated during the reaction). The phenol functions in the ligand contribute by stabilizing the intermediate through hydrogen bonding. If the phenols incorporate a hard metal such as Li, Mg, or Zn, the resulting metal phenoxide acts as a Brønsted base, enhancing the catalytic activity. The hard Lewis acidity of the metal phenoxide is also characteristic.

**Figure 3:**
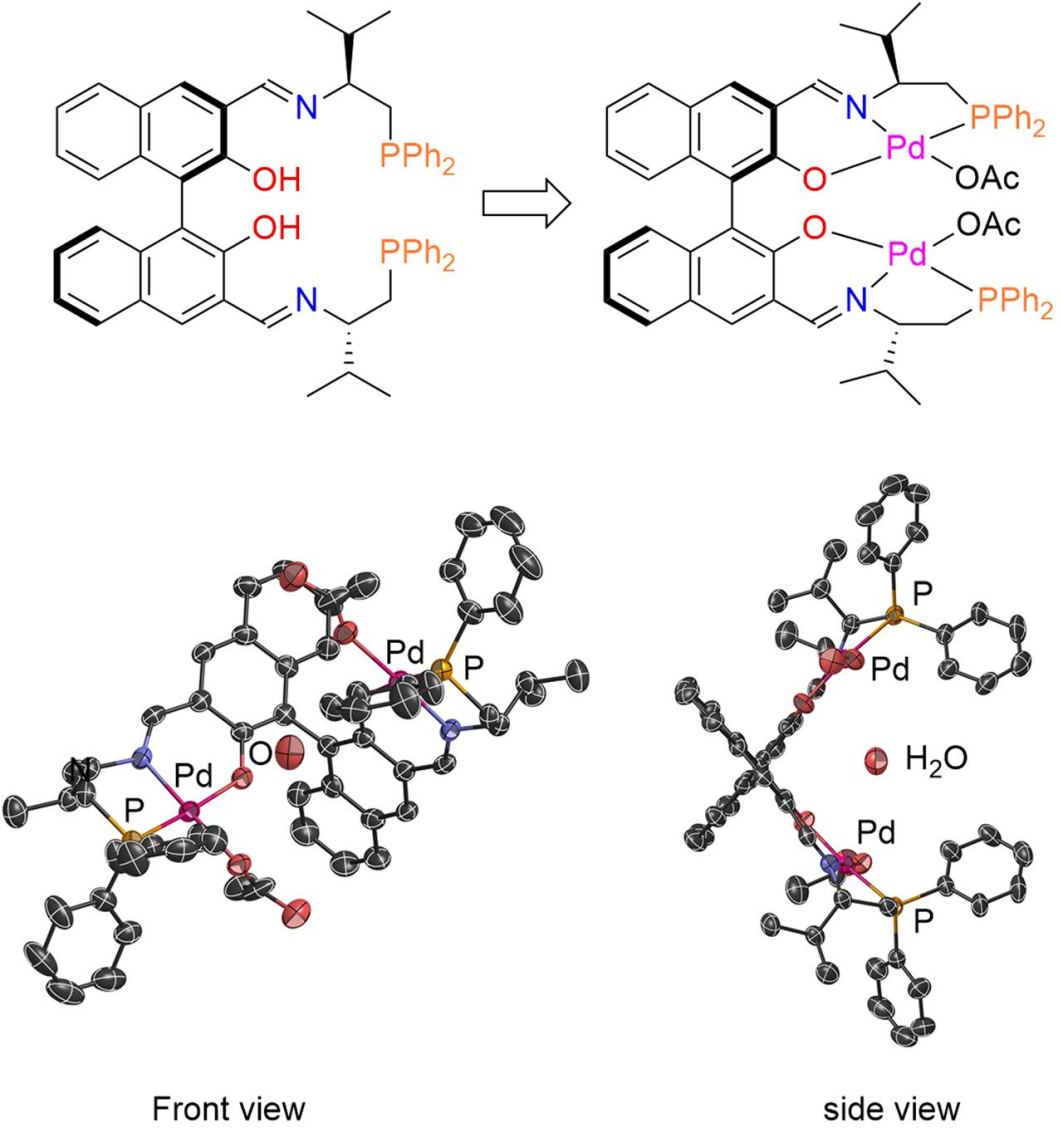
X-ray structure of **L1**-Pd_2_ (CCDC 1543349).

The synthesis of phosphoiminoBINOLs **L1**-**L4** can be readily achieved through imine formation between 3,3′-formyl BINOL and the corresponding aminophosphine (Fig. 2C). The isopropyl-substituted phosphoiminoBINOL (**L1**) was stable to handling under air, and showed a high affinity with various soft metal salts. When 2 mol equiv. of Pd(OAc)_2_ was added to **L1**, the formation of a dinuclear palladium complex took place, as demonstrated by ESI-MS: an ion peak was detected at *m*/*z* = 1117.1705 in CH_2_Cl_2_, and this was attributed to [**L1**(−2H) + Pd_2_(OAc)]^+^. Fine crystals were obtained from a CH_2_Cl_2_ solution, and the structure of phosphoiminobinaphthoxy (**L1**)-Pd_2_(OAc)_2_ was determined by X-ray crystallographic analysis (Fig. 3).

Based on this structure, one of the acetoxy anions of Pd(OAc)_2_ was replaced with **L1** to make a palladium acetoxy phenoxide. The distance between two palladium cations is 6.907 Å, which is suitable for binding malononitrile (or a generated anion of malononitrile), and there is one H_2_O molecule positioned at the center of the asymmetric sphere. **L1**-Pd_2_ reacted smoothly with malononitrile. The ^1^H NMR spectrum of free malononitrile in CDCl_3_ shows a single methylene peak at 3.60 ppm. When malononitrile is added to 1 equiv. of **L1**-Pd_2_, the peak was shifted to 2.43 ppm ^1^H NMR (see details in Supplementary Information). ESI-MS analysis of a 1:1 mixture of malononitrile with **L1**-Pd_2_ showed a clear new peak at *m*/*z* = 1123.1764, attributed to [**L1**(−2H) + Pd_2_ + NCCHCN]^+^.

With this fascinating container for malononitrile (**L1**-Pd_2_) in hand, the optimum catalyst for the conventional single Mannich reaction was examined before we attempted the challenge of the double Mannich reaction (Table 1)^8–23^.

**Table 1:** Development of dinuclear phosphoiminoBINOL-metal catalyst for Mannich reaction using malononitrile.



Entry	Ligand	Additive	Yield (%)^a^	2a/3a	ee of 2a (%)
1	**L1**	—	99	83/17	8
2	**L1**	LiOAc	86	84/16	10
3	**L1**	NaOAc	99	72/28	26
4	**L1**	Mg(OAc)_2_	91	57/43	74
5	**L1**	Ca(OAc)_2_	94	74/26	30
6	**L1**	Zn(OAc)_2_	93	60/40	68
7	**L1**	ZnCl_2_	88	92/8	7
8	**L1**	Zn(OTf)_2_	66	93/7	18
9	**L2**	Zn(OAc)_2_	46	96/4	11
10	**L3**	Zn(OAc)_2_	72	85/15	37
11	**L4**	Zn(OAc)_2_	78	68/32	77
12	**L5**	Zn(OAc)_2_	73	78/22	6
13^b)^	**L6**	Zn(OAc)_2_	75	90/10	10
14^b)^	**L7**	Zn(OAc)_2_	65	92/8	rac
15^c)^	**L1**	Zn(OAc)_2_	70	73/27	91

^a)^Conversion yield (see details for the determination in SI). ^b)^10 mol % of Pd(OAc)_2_ were used. ^c)^5 mol % of PhosphoiminoBINOL, 10 mol % of Pd(OAc)_2_, and 5 mol % of Zn(OAc)_2_ were used. Imine **1a** was slowly added over 4 h.

Although the Mannich reaction was smoothly catalyzed by the simple use of **L1**-Pd_2_, the Mannich product **2a** was obtained with only 8% ee. As we expected cooperative effects due to the phenoxy unit of **L1**-Pd_2_ (Fig. 2B), the addition of several hard metal salts was examined (Entries 2–8). Several metal acetates were effective in improving asymmetric induction. With assistance from Zn(OAc)_2_ or Mg(OAc)_2_, **L1**-Pd_2_ smoothly catalyzed the Mannich reaction to give **2a** with 68% ee and 74% ee, respectively. The structure-activity relationship of the ligands is also shown in Table 1. The equivalent catalyst using the methyl analog of **L1** ((**L2**)-Pd_2_) gave **2a** with only 11% ee (entry 9). Although the *t*-Butyl analog **L4**-Pd_2_ gave **2a** with 77% ee, the catalyst activity was reduced (entry 11). The diastereomeric ligand **L5** synthesized from (*S*)-BINOL, and **L6** and **L7** for constructing mono-nuclear palladium complex resulted in low levels of asymmetric induction. For **L1**-Pd_2_ with a Zn(OAc)_2_ catalyst system, when the *N*-Boc imine **1a** was added slowly, the Mannich adduct **2a** was obtained with 91% ee in 5 mol % catalyst use (entry 15).

**Figure 4:**
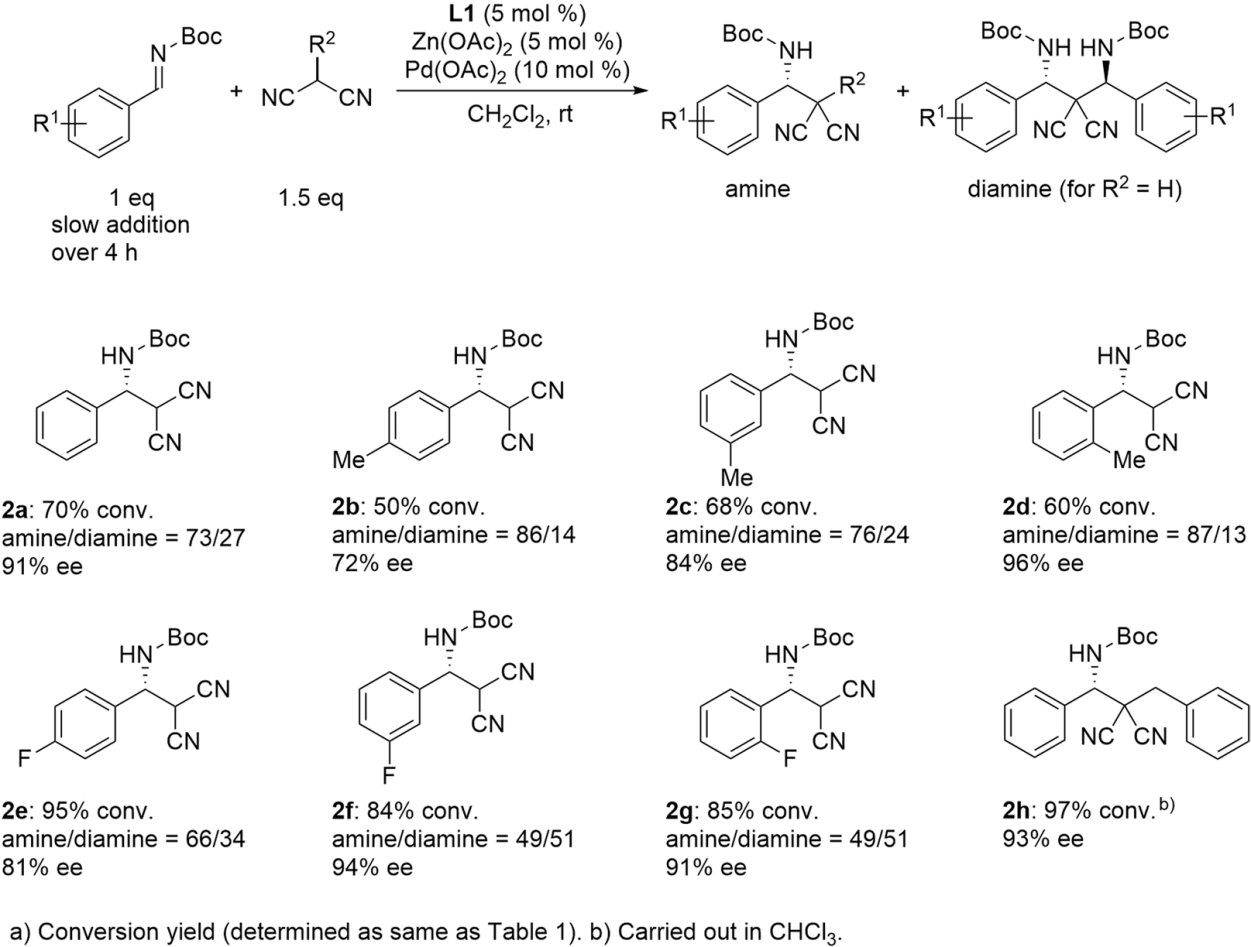
Dinuclear phosphoiminoBINOL-Pd catalyzed asymmetric Mannich reaction using malononitrile.

The results of the **L1**-Pd_2_-catalyzed asymmetric Mannich reaction under the optimized conditions are summarized in Fig. 4. Aromatic imines with various substituents were smoothly converted to the Mannich products with high enantioselectivity.

**Figure 5:**
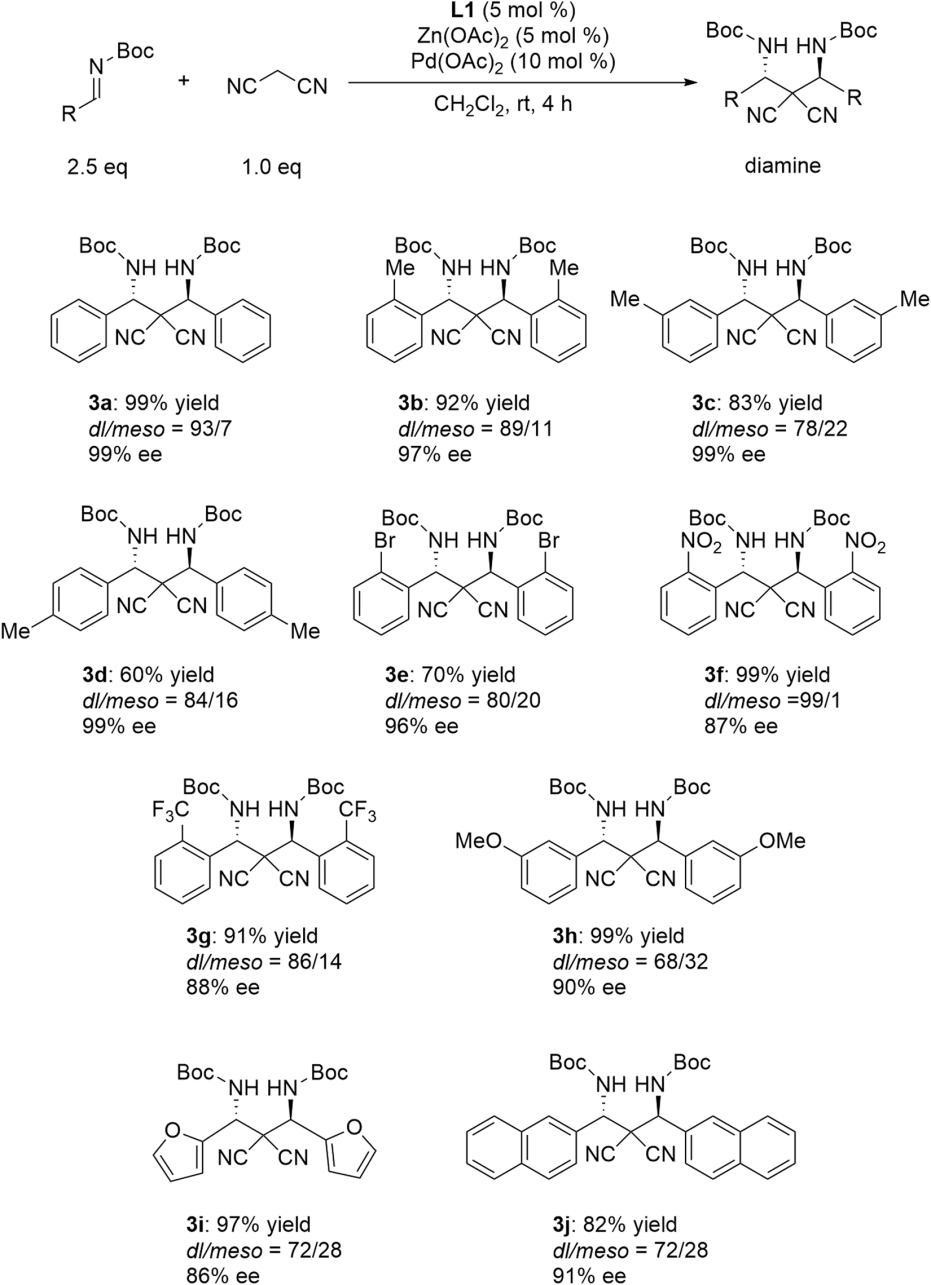
Catalytic asymmetric double Mannich reaction.

Using monobenzylated malononitrile, the chiral amine **2 h** having adjacent quaternary carbon center was obtained in 97% yield with 93% ee. This suggests the strong catalyst activity of the dinuclear **L1**-Pd_2_ complex. *We assumed that the highly active dinuclear* **L1**-*Pd*_2_ *catalyst would facilitate the conversion of the Mannich adducts* (**2a–d**) *to the second Mannich reaction for giving the 1*,*3-diamines* **3**. Actually, the use of **L1**-Pd_2_ with a Zn(OAc)_2_ catalyst system resulted in the production of a significant amount of the 1:2 adduct **3**, as desired. For the synthesis of **2f** and **2g**, co-production of the same amount of diamines was observed even when 1.5 equiv. of malononitrile was applied to the imine substrate^24,25^. For the double Mannich reaction, the reaction conditions were modified so that 2.5 equiv. of *N*-Boc imine were used with respect to the malononitrile. **L1**-Pd_2_ with Zn(OAc)_2_ catalyzed the double Mannich reaction quite smoothly to give the 1,3-diamine **3a** in quantitative yield with high diastereoselectivity (*dl*/*meso* = 93/7)^26–43^. The major *dl*-isomer was obtained in 99% ee. The results of 1,3-diamine synthesis by the double Mannich reaction are summarized in Fig. 5.

For *N*-Boc-imines derived from both electron-donating and electron-deficient benzaldehydes, chiral 1,3-diamines were obtained in a highly enantioselective manner. A chiral bisfuryl-1,3-diamine(**3i**) was obtained with 86% ee, and a bisnaphthyl-1,3-diamine(**3j**) was also obtained successfully with 91% ee. When the second Mannich reaction was examined using *rac*-**2a** with *N*-Boc-imine, **3a** was obtained in 73% ee with increasing co-production of the *meso*-form in *dl*/*meso* = 1:1. This result suggests that the second Mannich reaction is catalyzed independently from the first Mannich reaction, and the enantiomeric excess of **3a** is improved due to the *meso*-trick^44^.

**Figure 6:**
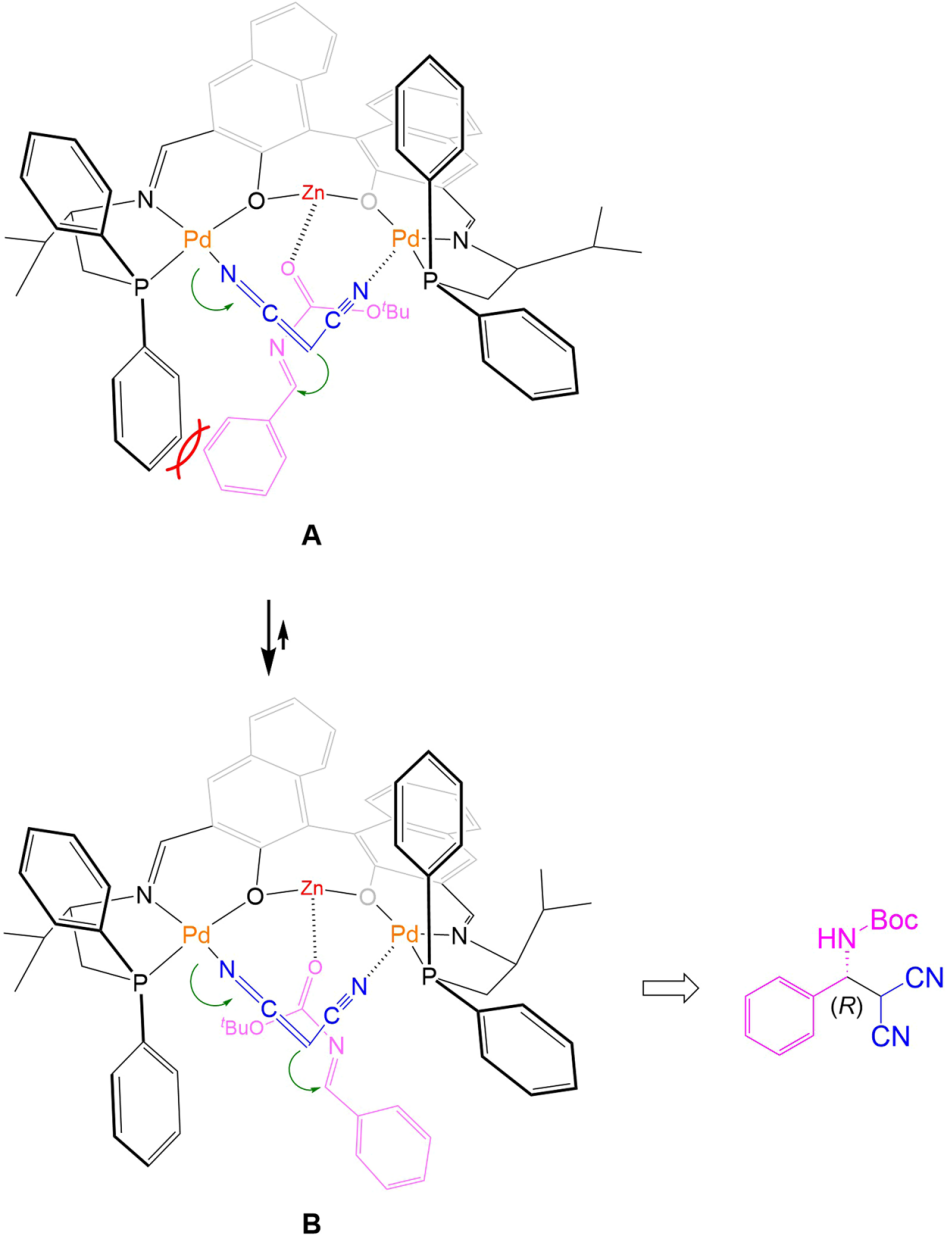
Plausible reaction model for asymmetric Mannich reaction using **L1**-Pd_2_.

The formation of the (*R*)-enriched Mannich adduct using **L1** can be explained using the working model described in Fig. 6.

The dinuclear **L1**-Pd_2_ complex binds to malononitrile at both nitrogen atoms of the nitrile moieties. Although the formation of palladium enolate by simple mixing of malononitrile with **L1**-Pd_2_ is suggested by the detection of an ion peak for [**L1**(−2H) + Pd_2_ + NCCHCN]^+^ at *m*/*z* = 1123.1764, the addition of Zn(OAc)_2_ also assists the smooth formation of the palladium enolate. The low asymmetric induction and catalyst activity of the mono-nuclear palladium complex using **L6** and **L7** suggest the effective role of the dinuclear palladium complex for converting **2a** to **3a** (Table 1, entries 13, 14) Moreover, because asymmetric induction of the Mannich adducts **2a** and **3a** is strongly influenced by the selection of hard metal salts, as shown in Table 1, a Zn(OAc)_2_-driven reactant is incorporated in the asymmetric reaction sphere^45–53^. When the hard zinc atom is captured by the two hard phenoxy oxygens of **L1**-Pd_2_, the zinc atom can act as a Lewis acidic site for activating Boc-imines. During nucleophilic attack by the Pd-enolate malononitrile of the zinc-activated *N*-Boc imine, the approach shown in model A (Fig. 6) involves serious steric repulsion between a benzene ring from the phosphine in **L1** and the *N*-Boc imine. By flipping the face of the *N*-Boc imine, as shown in Model B, the steric repulsion can be released, and nucleophilic attack of the *Re*-face of the *N*-Boc imine gives the (*R*)-enriched Mannich product.

In conclusion, the dinuclear bis(phosphoimino)binaphthoxy-Pd_2_(OAc)_2_ complex described herein facilitated a double Mannich reaction of *N*-Boc-imine with malononitrile to give chiral 1,3-diamines in a highly enantioselective manner. This demonstrates that over-reaction need not always be useless and undesired: well managed over-reaction can open up novel synthetic processes.

## Electronic supplementary material

Supporting Information
