# Supplementary material for: Dinuclear PhosphoiminoBINOL-Pd Container for Malononitrile: Catalytic Asymmetric Double Mannich Reaction for Chiral 1,3-Diamine Synthesis
Source: Sci Rep. 2018 Jan 16;8:837. doi: 10.1038/s41598-018-19178-4 (PMC5770441; doi:10.1038/s41598-018-19178-4)
Supplement: Supplementary file 1 — Supporting Information [file 41598_2018_19178_MOESM1_ESM.pdf]

Supporting information

Dinuclear PhosphoiminoBINOL-Pd Container for Malononitrile: Catalytic Asymmetric Double Mannich Reaction for Chiral 1,3-Diamine Synthesis

Authors:

Takayoshi Arai,[1]* Katsuya Sato,[1] Ayu Nakamura,[1] Hiroki Makino,[1] Hyuma Masu[2]

Affiliations:

[1] Soft Molecular Activation Research Center (SMARC), Molecular Chirality Research Center (MCRC), and Department of Chemistry, Graduate School of Science, Chiba University, 1-33 Yayoi, Inage, Chiba 263-8522, Japan.

[2] Center for Analytical Instrumentation, Chiba University, 1-33 Yayoi, Inage, Chiba 263-8522, Japan.

*Correspondence to: tarai@faculty.chiba-u.jp

Contents

1. General

2. Synthesis of iminophosphine ligands

3. General procedure of enantioselective Mannich reaction

4. Analytical data for product of Mannich reaction

5. General procedure of enantioselective Double Mannich reaction

6. Analytical data for product of Double Mannich reaction

7. ^{1}H and ^{13}C spectra

8. HPLC spectra

9.NMR and MS spectra of the interaction between catalyst and malononitrile

10. CIF data of 3,3’-bisphosphoiminobinaphthol-$Pd_2(OAc)_2$

11. References

1. General

Dry solvents were purchased from commercial suppliers and used without further purification. Analytical thin-layer chromatography (TLC) was performed on glass plates coated with 0.25 mm 230-400 mesh silica gel containing a fluorescent indicator (Merck, #1.05715.0009). Silica-gel column chromatography was performed on Kanto silica gel 60 (spherical, 100-210 μm), Kanto silica gel 60 (spherical, 63-210 μm). IR spectra were recorded on JASCO FT/IR-4100 using ATR. ^{1}H-NMR spectra were recorded on JEOL ECS-400 (400MHz), ECA-500 (500MHz), ECX-400 (400MHz) spectrometers. Chemical shifts of ^{1}H-NMR spectra were reported relative to tetramethyl silane (δ 0). ^{13}C-NMR spectra were recorded on JEOL ECS-400 (100MHz), ECA-500 (125MHz), ECX-400 (100MHz) spectrometers. Chemical shifts of ^{13}C-NMR spectra were reported relative to $CDCl_3$ (δ 77.0). Splitting patterns were reported as s, singlet; d, doublet; t, triplet; q, quartet; m, multiplet; br, broad.

2. Synthesis of iminophosphine ligands

***(R)*-3,3'-bis(*(E)*-(((*S*)-1-(diphenylphosphanyl)-3-methylbutan-2-yl)imino)methyl)-[1,1'-binaphthalene]-2,2'-diol (L1)**

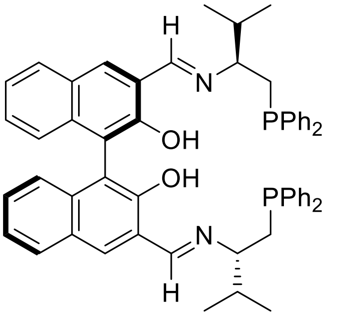

A mixture of (*R*)-2,2'-dihydroxy-[1,1'-binaphthalene]-3,3'-dicarbaldehyde [1)] (0.20 mmol) and (S)-1-(diphenylphosphanyl)-3-methylbutan-2-amine [2)] (0.44 mmol) in ethanol (30 mL) was heated to reflux. After being stirred for 24 hours, the solvent was removed under reduced pressure and the resulting residue was washed with cold ethanol to obtain **L1** as a yellow solid. ^{1}H NMR (400MHz, $CDCl_3$) δ 8.39 (s, 2H) 7.87 (s, 2H), 7.83-7.86 (m, 2H), 7.44-7.49 (m, 4H), 7.34-7.38 (m, 10H), 7.15-7.29 (m, 14H), 3.07-3.14 (m, 2H), 2.48 (dd, *J*=14.0, 4.5 Hz, 2H), 2.35 (dd, *J*=13.9, 8.3 Hz, 2H), 1.95-2.07 (m, 2H), 0.91 (d, *J*=2.92 Hz, 6H), 0.89 (d, *J*=2.92 Hz, 6H); ^{13}C NMR (100MHz, $CDCl_3$) δ 164.5, 154.8, 138.3, 138.2, 138.0, 135.2, 133.4, 133.2, 133.0, 132.8, 132.6, 128.8, 128.7, 128.6 128.5, 128.4, 128.0, 127.4, 124.9, 123.1, 120.9, 116.5, 73.4, 73.3, 33.6, 32.9, 32.8, 19.8, 17.6; HRMS calcd for $C_{56}H_{54}N_2O_2P_2$ $(M+H)^+$: 849.3689, found: 849.3745 ; $[\alpha]_D^{20.0}$ = +141.2 (c=1.0, $CHCl_3$); IR (neat) 2973,1699, 1508, 1495, 1464, 1367, 1323, 1247, 1160, 1051, 743 cm^{-1}.

***(R)*-3,3'-bis(*(E)*-(((*S*)-1-(diphenylphosphanyl)propan-2-yl)imino)methyl)-[1,1'-binaphthalene]-2,2'-diol (L2)**

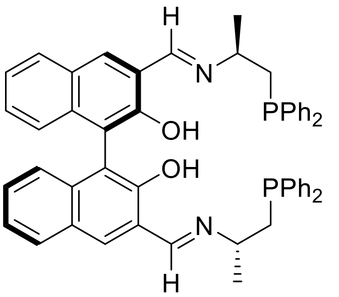

A mixture of (*R*)-2,2'-dihydroxy-[1,1'-binaphthalene]-3,3'-dicarbaldehyde (0.3 mmol) and (S)-1-(diphenylphosphanyl)propan-2-amine (0.75 mmol) in ethanol (12 mL) was heated to reflux. After being stirred for 21 hours, the solvent was removed *in vacuo* and the residue was purified by silica-gel column chromatography (Hexane/AcOEt = 6/1). The mixture was dissolved in ethanol and cooled to -20°C. The reprecipitated solid was filtered and washed with cooled ethanol to obtain **L2** as yellow solid. ^{1}H NMR (400MHz, $CDCl_3$) δ 8.47 (s, 2H), 7.90-7.84 (m, 4H), 7.44-7.38 (m, 8H), 7.31-7.26 (m, 18H) 7.19-7.17 (m, 2H), 3.53-3.46 (m, 2H), 2.45 (dd, *J*=13.8, 6.1 Hz, 2H), 2.34 (dd, *J*=13.7, 7.1 Hz, 2H), 1.41 (d, *J*=6.3 Hz, 6H); ^{13}C NMR (100MHz, $CDCl_3$): δ163.1, 154.7, 138.4, 138.3, 138.1, 137.9, 135.2, 133.2, 132.9, 132.8, 132.7, 132.6, 128.8, 128.6, 128.5, 128.2, 127.5, 124.7, 123.2, 120.9, 116.5, 62.9, 62.8, 37.5, 37.4, 23.6, 23.5; HRMS calcd for $C_{52}H_{47}N_2O_2P_2$ $(M+H)^+$: 793.3107, found: 793.3090 ; $[\alpha]_D^{27.0}$ = +83.3 (c=0.1, $CHCl_3$); IR (neat) 3056, 1632, 1506, 1434, 1384, 1344, 1254, 1217, 1119, 754, 698 cm^{-1}.

***(R)*-3,3'-bis(*(E)*-(((*S)*-1-(diphenylphosphanyl)-3-phenylpropan-2-yl)imino)methyl)-[1,1'-binaphthalene]-2,2'-diol (L3)**

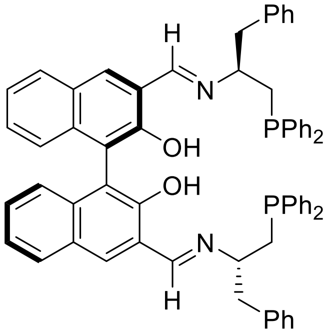

A mixture of (*R*)-2,2'-dihydroxy-[1,1'-binaphthalene]-3,3'-dicarbaldehyde (0.63 mmol) and (S)-1-(diphenylphosphanyl)-3-phenylpropan-2-amine (1.40 mmol) in ethanol (25 mL) was heated to reflux. After being stirred for 20 hours, the solvent was removed under reduced pressure and the resulting residue was washed with cold ethanol. The solid was mixed with (S)-1-(diphenylphosphanyl)-3-phenylpropan-2-amine (0.012 mmol) in ethanol (5 ml). The mixture was heated to reflux. After being stirred for 1 hour, the solvent was removed under reduced pressure and the resulting residue was washed with cold ethanol to obtain **L3** as yellow solid. ^{1}H NMR (400MHz, $CDCl_3$) δ 8.02 (s, 2H), 7.81 (d, *J*=7.7 Hz, 2H), 7.71 (s, 2H), 7.38-7.13 (m, 34H), 7.04 (d, *J*=6.8 Hz, 4H), 3.49-3.41 (m, 2H), 3.17 (dd, *J*=13.7, 4.4 Hz, 2H), 2.89 (dd, *J*=13.5, 8.7 Hz, 2H), 2.52 (dd, *J*=13.8, 5.9 Hz, 2H), 2.43 (dd, *J*=13.8, 7.3 Hz, 2H); ^{13}C NMR (100MHz, $CDCl_3$): δ 164.4, 154.6, 138.3, 135.1, 133.3, 132.9, 132.8 132.7, 132.6, 129.7, 128.8, 128.7, 128.6, 128.5, 128.2, 128.1, 127.4, 126.3, 124.8, 123.2, 120.6, 116.5, 69.6, 69.5, 44.0, 43.9, 35.3, 35.1; HRMS calcd for $C_{64}H_{55}N_2O_2P_2$ $(M+H)^+$: 945.3733, found: 945.3723 ; $[\alpha]_D^{18.3}$= -12.7 (c=1.0, $CHCl_3$); IR (neat) 3057, 1632, 1505, 1435, 1343, 752, 699 cm^{-1}.

***(R)*-3,3'-bis(*(E)*-(((*S)*-1-(diphenylphosphanyl)-3,3-dimethylbutan-2-yl)imino)methyl)-[1,1'-binaphthalene]-2,2'-diol (L4)**

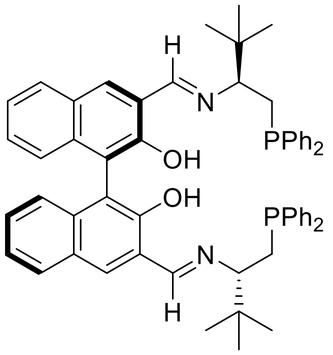

A mixture of (R)-2,2'-dihydroxy-[1,1'-binaphthalene]-3,3'-dicarbaldehyde (0.30 mmol) and (S)-1-(diphenylphosphanyl)-3,3-dimethylbutan-2-amine (0.67 mmol) in ethanol (3 mL) was heated to reflux. After being stirred for 15 hours, the solvent was removed under reduced pressure and the resulting residue was washed with cold ethanol to obtain **L4** as yellow solid. ^{1}H NMR (400MHz, $CDCl_3$) δ 8.37 (s, 2H), 7.87-7.84 (m, 4H), 7.55-7.51 (m, 4H), 7.42-7.38 (m, 6H), 7.33-7.13 (m, 18H), 2.93 (t, *J*=10.8 Hz, 2H), 2.53 (d, J=15.6, 2H), 2.29-2.22 (m, 2H), 0.891 (s, 18H); ^{13}C NMR (100MHz, $CDCl_3$): δ 165.0, 154.9, 138.5, 138.4, 137.9, 137.7, 135.3, 133.8, 133.6, 132.5, 132.3, 129.0, 128.7, 128.6, 128.5, 128.4, 128.3, 128.0, 127.4, 124.9, 123.0, 120.8, 116.4, 35.1, 35.0, 30.2, 30.1, 26.6; HRMS calcd for $C_{58}H_{59}N_2O_2P_2$ $(M+H)^+$: 877.4046, found: 877.4024; $[\alpha]_D^{21.1}$= +218.603 (c=1.0, $CHCl_3$); IR (neat) 2958, 1629, 1433, 1342, 1254, 1001, 736, 694 cm^{-1}.

***(S)*-3,3'-bis(*(E)*-(((*S*)-1-(diphenylphosphanyl)-3-methylbutan-2-yl)imino)methyl)-[1,1'-binaphthalene]-2,2'-diol (L5)**

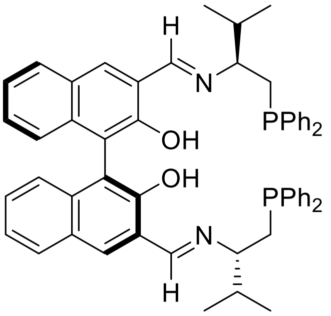

A mixture of (*S*)-2,2'-dihydroxy-[1,1'-binaphthalene]-3,3'-dicarbaldehyde (0.44 mmol) and (S)-1-(diphenylphosphanyl)-3-methylbutan-2-amine (0.88 mmol) in ethanol (8 mL) was heated to reflux. After being stirred for 24 hours, the solvent was removed under reduced pressure and the resulting residue was washed with cold ethanol to obtain **L5** as a yellow solid. ^{1}H NMR (400MHz, $CDCl_3$) δ 8.39 (s, 2H), 7.86-7.84 (m, 4H), 7.46-7.42 (m, 4H), 7.37-7.25 (m, 16H), 7.23-7.15 (m, 8H), 3.19-3.12 (m, 2H) 2.43-2.39 (m, 4H), 2.00-1.92 (m, 2H), 0.91 (d, *J*=4.1 Hz, 6H), 0.89 (d, *J*=4.1 Hz, 6H) ;^{13}C NMR (100MHz, $CDCl_3$): δ 164.6, 154.7, 138.4, 138.2, 135.1, 133.5, 133.1 132.9, 132.7, 132.5 128.8, 128.6, 128.5, 128.4, 128.3, 128.1, 127.5, 125.0, 123.1, 120.8, 116.3, 74.0, 73.8, 34.0, 32.7, 32.5, 19.5, 17.8; HRMS calcd for $C_{56}H_{55}N_2O_2P_2(M+H)^+$: 849.3733, found: 849.3698 ; $[\alpha]_D^{27.8}$ = +82.5 (c=0.1, $CHCl_3$); IR (neat) 3389, 2969, 1631, 1434, 1339, 739, 695 cm^{-1}.

***(R)*-3-(*(E)*-(((*S*)-1-(diphenylphosphanyl)-3-methylbutan-2-yl)imino)methyl)-[1,1'-binaphthalene]-2,2'-diol (L6)**

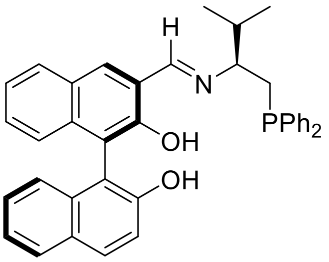

A mixture of (R)- 2,2'-dihydroxy-[1,1'-binaphthalene]-3-carbaldehyde (0.5 mmol) and (S)-1-(diphenylphosphanyl)-3-methylbutan-2-amine (0.6 mmol) in ethanol (10 mL) was heated to reflux. After being stirred for 23 hours, the solvent was removed *in vacuo* and the residue was purified by silica-gel column chromatography (Hexane/AcOEt = 3/1) to obtain **L6** as orange solid. ^{1}H NMR (400MHz, $CDCl_3$) δ 8.41 (s, 1H), 7.92-7.84 (m, 4H), 7.46-7.07 (m, 18H), 5.12 (s, 1H), 3.25-3.18 (m, 1H), 2,49-2.40 (m, 2H), 2.01-1.92 (m, 1H), 0.91 (d, *J*=6.8, 6H); ^{13}C NMR (100MHz, $CDCl_3$): δ 164.1, 155.6, 151.5, 135.2, 134.4, 133.5, 133.0, 132.8, 130.0, 129.3, 128.8, 128.6, 128.5, 128.4, 128.3, 128.2, 127.5, 126.4, 125.0, 124.8, 123.8, 123.2, 120.6, 117.7, 114.4, 113.2, 74.2, 74.0, 34.1, 34.0, 32.5, 32.4, 19.6, 17.9; HRMS calcd for $C_{38}H_{34}NO_2P$ $(M+H)^+$: 568.2400, found: 568.2382; $[\alpha]_D^{20.8}$= +123.031 (c=1.0, $CHCl_3$); IR (neat) 3522, 2960, 1630, 1509, 1466, 1434, 1348, 1195, 1136, 999, 815, 743, 695 cm^{-1}.

***(S,E)*-2-(((1-(diphenylphosphanyl)-3-methylbutan-2-yl)imino)methyl)phenol (L7)**

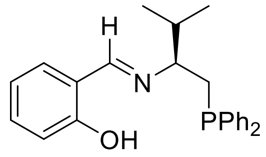

A mixture of 2-hydroxybenzaldehyde (1 mmol) and (S)-1-(diphenylphosphanyl)-3-methylbutan-2-amine (1.1 mmol) in dichloromethane (10 ml) was stirred at room temperature for 17 hours. The solvent was removed *in vacuo*, and the residue was purified by silica-gel column chromatography (Hexane/AcOEt = 10/1) to obtain **L7** as yellow oil. ^{1}H NMR (400MHz, $CDCl_3$) δ 8.09 (s, 1H), 7.46-7.16 (m, 13H), 6.91 (d, *J*=8.2 Hz, 1H), 6.85 (dt, *J*=7.5, 0.5 Hz, 1H), 3.06 (sep, *J*=4.5 Hz, 1H), 2.48 (dd, *J*=14.0, 3.6 Hz, 1H), 2.41 (dd, *J*=13.8, 8.8 Hz, 1H), 2.05-1.94 (m, 1H), 0.95 (d, *J*=6.8 Hz, 3H), 0.90 (d, *J*=6.8 Hz, 3H) ;^{13}C NMR (100MHz, $CDCl_3$): δ 164.2, 161.2, 138.2, 138.0, 133.1, 132.9, 132.8, 132.6, 132.1, 131.3, 128.8, 128.6, 128.5, 128.4, 128.3, 118.6, 118.3, 116.9, 73.1, 73.0, 33.9, 33.8, 33.1, 33.0, 19.7, 17.5 ; HRMS calcd for $C_{24}H_{27}NOP(M+H)^+$: 376.1825, found: 376.1815 ; $[\alpha]_D^{27.5}$ = +107.2 (c=1.0, $CHCl_3$); IR (neat) 3051, 2955, 1631, 1581, 1496, 1462, 1433, 1282, 999, 853, 754, 696 cm^{-1}.

3. General procedure of enantioselective Mannich reaction.

A mixture of 3,3'-bis(phosphoimino)binaphthol (0.005 mmol) and $Pd(OAc)_2$ (0.01 mmol) was stirred for 12 hour in anhydrous dichloromethane (4.0 ml) at rt. Arter that, $Zn(OAc)_2$ (0.005 mmol) was added and stirred for 12 hours at rt. Malononitrile (0.15 mmol) added to the resulting red solution. Then, *N*-Boc imine (0.1 mmol) in dichloromethane (1 ml) was added over 4 hours. After being stirred for appropriate time, the reaction mixture was quenched with H_2O, and then the products were extracted with dichloromethane in 3 times. The collected organic layer was dried over Na_2SO_4. After removal of the solvent under reduced pressure, the residue was purified by silica-gel column chromatography. The enantiomeric excesses of the products were determined by chiral stationary phase HPLC using a Daicel Chiralcel OD-H and Chiralpak IA column.

[For calculating the conversion yield of Table 1 and Fig. 4]

The molar ratio between amine and diamine (1:d) was analyzed from the intensity of ^{1}H-NMR spectra for the mixture of products. Based on the molar ratio, each chemical yields of the amine (A mmol) and the diamine (A x d mmol) was determined on the isolated amount of the products. Finally, the conversion yield (A + A x 2d) was calculated as the consumption of imine substrates.

4. Analytical data for product of Mannich reaction and Double Mannich reaction

***tert*-butyl *(R)*-(2,2-dicyano-1-phenylethyl)carbamate (2a)**

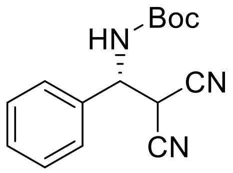

^{1}H NMR (400 MHz, $CDCl_3$): δ 7.48 (s, 5H), 5.27 (d, *J*= 5.9 Hz, 1H), 5.09 (t, *J*= 5.4 Hz, 1H), 4.82 (d, *J*= 4.3 Hz, 1H), 1.48 (s, 9H). The NMR spectra was identical to those previously reported. $[\alpha]_D^{20.2}$= +17.4° (c=0.1, $CHCl_3$, 91% ee). Enantiomeric excess was determined by HPLC with a Chiralpak IA column (hexane:2-propanol = 90:10, 0.5 mL/min, 254 nm) ; major enantiomer t_r = 21.8 min, minor enantiomer t_r = 35.0 min, 91% ee.

***tert*-butyl *(R)*-(2,2-dicyano-1-(*p*-tolyl)ethyl)carbamate (2b)**

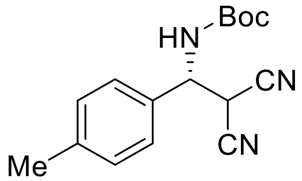

^{1}H NMR (400 MHz, $CDCl_3$): δ 7.36 (d, *J*= 7.9 Hz, 2H), 7.27 (d, *J*= 8.2 Hz, 2H), 5.27 (br, 1H), 5.09 (t, *J*= 5.4 Hz, 1H), 4.82 (d, *J*= 4.1 Hz, 1H), 2.39 (s, 3H), 1.47 (s, 9H) The NMR spectra was identical to those previously reported. $[\alpha]_D^{27.7}$= +8.2° (c=0.1, $CHCl_3$, 72% ee). Enantiomeric excess was determined by HPLC with a Chiralpak IA column (hexane:2-propanol = 90:10, 0.3 mL/min, 254 nm) ; major enantiomer t_r = 34.0 min, minor enantiomer t_r = 46.1 min, 72% ee.

***tert*-butyl *(R)*-(2,2-dicyano-1-(*m*-tolyl)ethyl)carbamate (2c)**

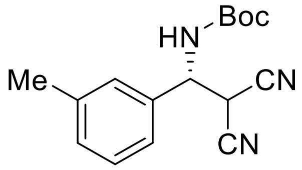

^{1}H NMR (400 MHz, $CDCl_3$): δ 7.39-7.32 (m, 1H), 7.30-7.21 (m, 3H), 5.30 (br, 1H), 5.09-5.01 (m 1H), 4.78 (br, 1H), 2.40 (s, 3H) 1.48 (s, 9H). The NMR spectra was identical to those previously reported. $[\alpha]_D^{19.7}$= +17.6° (c=0.1, $CHCl_3$, 84% ee). Enantiomeric excess was determined by HPLC with a Chiralpak IA column (hexane:2-propanol = 90:10, 0.5 mL/min, 254 nm) ; major enantiomer t_r = 18.9 min, minor enantiomer t_r = 31.3 min, 84% ee.

***tert*-butyl *(R)*-(2,2-dicyano-1-(*o*-tolyl)ethyl)carbamate (2d)**

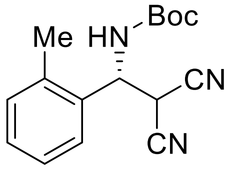

^{1}H NMR (400 MHz, $CDCl_3$): δ 7.55 (br, 1H), 7.36-7.33 (m, 2H), 7.30-7.27 (m, 1H), 5.47 (t, *J*= 6.1 Hz, 1H), 5.17 (br, 1H), 4.74 (d, *J*= 4.5 Hz, 1H), 2.45 (s, 3H), 1.48 (s, 9H). The NMR spectra was identical to those previously reported. $[\alpha]_D^{18.2}$= +39.6° (c=0.5, $CHCl_3$, 84% ee). Enantiomeric excess was determined by HPLC with a Chiralpak IA column (hexane:2-propanol = 95:5, 0.3 mL/min, 254 nm) ; major enantiomer t_r = 66.2 min, minor enantiomer t_r = 62.8 min, 96% ee.

tert-butyl (R)-(2,2-dicyano-1-(4-fluorophenyl)ethyl)carbamate (2e)

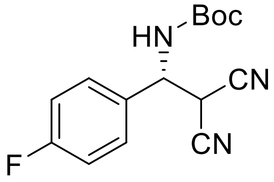

^{1}H NMR (400 MHz, $CDCl_3$): δ 7.49 (dd, *J*= 8.6, 5.0 Hz, 2H), 7.17 (dt, *J*= 8.6, 8.5, 1.4 Hz, 2H), 5.26 (br, 1H), 5.12-5.06 (m, 1H), 4.82 (br, 1H), 1.48 (s, 9H). The NMR spectra was identical to those previously reported. $[\alpha]_D^{18.9}$= +19.5° (c=0.1, $CHCl_3$, 81% ee). Enantiomeric excess was determined by HPLC with a Chiralpak IA column (hexane:2-propanol = 90:10, 0.5 mL/min, 254 nm) ; major enantiomer t_r = 21.5 min, minor enantiomer t_r = 37.0 min, 81% ee.

tert-butyl (R)-(2,2-dicyano-1-(3-fluorophenyl)ethyl)carbamate (2f)

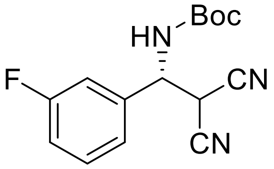

^{1}H NMR (400 MHz, $CDCl_3$): δ 7.47 (dt, *J*= 7.8, 5.9 Hz, 1H), 7.28 (d, *J*= 8.2 Hz, 1H), 7.23-7.16 (m, 2H), 5.26 (br, 1H), 5.11 (t, *J*= 5.2 Hz, 1H), 4.80 (br, 1H), 1.48 (s, 9H). The NMR spectra was identical to those previously reported. $[\alpha]_D^{18.8}$ = +5.2° (c=0.1, $CHCl_3$, 94% ee). Enantiomeric excess was determined by HPLC with a Chiralcel OD-H column (hexane:2-propanol = 90:10, 0.5 mL/min, 254 nm) ; major enantiomer t_r = 22.3 min, minor enantiomer t_r = 57.4 min, 94% ee.

tert-butyl (R)-(2,2-dicyano-1-(2-fluorophenyl)ethyl)carbamate (2g)

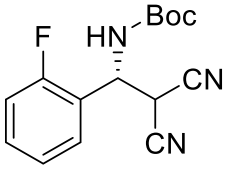

^{1}H NMR (400 MHz, $CDCl_3$): δ 7.53-7.43 (m, 2H), 7.23 (dt, *J*= 7.6, 1.1 Hz, 1H), 7.19 (ddd, *J*= 10.8, 8.4, 1.1 Hz, 1H), 5.56 (t, *J*= 7.0 Hz, 1H), 5.43 (d, *J*= 7.0 Hz, 1H), 4.57 (d, *J*= 5.7 Hz, 1H), 1.48 (s, 9H). The NMR spectra was identical to those previously reported. $[\alpha]_D^{18.8}$= +14.9° (c=0.1, $CHCl_3$, 91% ee). Enantiomeric excess was determined by HPLC with a Chiralcel OD-H column (hexane:2-propanol = 70:30, 0.3 mL/min, 254 nm) ; major enantiomer t_r = 24.5 min, minor enantiomer t_r = 22.1 min, 91% ee.

tert-butyl (S)-(2,2-dicyano-1,3-diphenylpropyl)carbamate (2h)

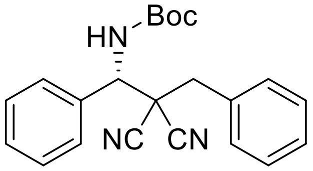

^{1}H NMR (400 MHz, $CDCl_3$): δ 7.51-7.45 (m, 5H), 7.41-7.38 (m, 5H), 5.54 (d, *J*= 9.60 Hz, 1H), 5.32 (d, *J*= 9.60 Hz, 1H), 3.27 (d, *J*= 14.0 Hz, 1H), 3.17 (d, *J*= 13.6 Hz, 1H), 1.47 (s, 9H); ^{13}C NMR (100 MHz, $CDCl_3$): δ 154.8, 130.1, 129.4, 128.5, 127.0, 111.1, 110.8, 81.7, 55.5, 29.6, 28.1; IR (neat) 3347, 2980, 1706, 1521, 1498, 1368, 1250, 1167, 762, 705 cm^{-1}; $[\alpha]_D^{20.4}$= +30.5° (c=1.0, $CHCl_3$, 93% ee).

Enantiomeric excess was determined by HPLC with a Chiralpak IA column (hexane:2-propanol = 80:20, 1.0 mL/min, 254 nm) ; major enantiomer t_r = 9.73 min, minor enantiomer t_r = 6.03 min, 93% ee; HRMS (ESI+) calcd for $C_{22}H_{23}O_2N_3$ $(M+H)^+$: 362.1863, found: 362.1860.

5. General procedure of enantioselective Double Mannich reaction.

A mixture of 3,3'-bis(phosphoimino)binaphthol (0.005 mmol) and $Pd(OAc)_2$ (0.01 mmol) was stirred for 12 hour in anhydrous dichloromethane (4.0 ml) at rt. Arter that, $Zn(OAc)_2$ (0.005 mmol) was added and stirred for 12 hours at rt. Malononitrile (0.1 mmol) added to the resulting red solution. Then, *N*-Boc imine (0.25 mmol) was added. After being stirred for appropriate time, the reaction mixture was quenched with H_2O, and then the products were extracted with dichloromethane in 3 times. The collected organic layer was dried over Na_2SO_4. After removal of the solvent under reduced pressure, the residue was purified by silica-gel column chromatography. The enantiomeric excesses of the products were determined by chiral stationary phase HPLC using a Daicel Chiralcel OD-H, Chiralpak IA, Chiralpak AD-H and Chiralpak AS-H column.

6. Analytical data for product of Double Mannich reaction

di-*tert*-butyl (*(1S,3S)*-2,2-dicyano-1,3-diphenylpropane-1,3-diyl)dicarbamate (3a)

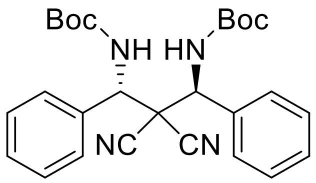

^{1}H NMR (400 MHz, $CDCl_3$): δ 7.42 (s, 10H), 5.53 (d, *J*=10.2 Hz, 2H), 5.33 (br, 2H), 1.44 (s, 18H). The NMR spectra was identical to those previously reported. Enantiomeric excess was determined by HPLC with a Chiralpak IA column (hexane:2-propanol = 90:10, 0.5 mL/min, 254 nm) ; major enantiomer t_r = 17.5 min, minor enantiomer t_r = 19.1 min, 99% ee.

di-*tert*-butyl (*(1S,3S)*-2,2-dicyano-1,3-di-*o*-tolylpropane-1,3-diyl)dicarbamate (3b)

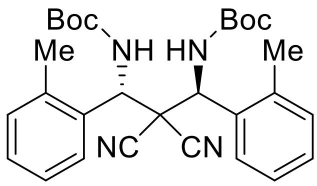

^{1}H NMR (400 MHz, $CDCl_3$): δ 7.77 (d, *J*=7.25, 2H), 7.33-7.28 (m, 4H), 7.16 (d, *J*=7.3 Hz, 2H), 5.68 (br, 2H), 5.36 (d, *J*=10.0 Hz, 2H) 2.03 (s, 6H), 1.46 (s, 18H) ; ^{13}C NMR (100 MHz, $CDCl_3$): δ 154.2, 137.2, 134.2, 131.3, 129.3, 127.2, 125.3, 113.6, 81.3, 51.8, 51.2, 28.1, 19.1; IR (neat) 3244, 2915, 1705, 1346, 1248, 1161, 1088, 1024, 891, 728 cm^{-1}. ; $[\alpha]_D$ $^{27.8}$= +61.1° (c=1.0, $CHCl_3$, 97% ee).

Enantiomeric excess was determined by HPLC with a Chiralpak IA column (hexane:2-propanol = 90:10, 1.0 mL/min, 254 nm) ; major enantiomer t_r = 18.7 min, minor enantiomer t_r = 22.0 min, 97% ee; HRMS (ESI+) calcd for $C_{29}H_{37}O_4N_4$ $(M+H)^+$: 505.2815, found: 505.2819.

di-*tert*-butyl (*(1S,3S)*-2,2-dicyano-1,3-di-*m*-tolylpropane-1,3-diyl)dicarbamate (3c)

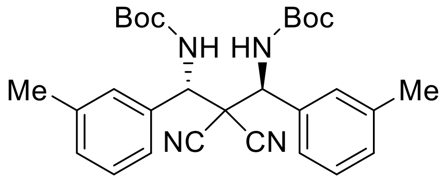

^{1}H NMR (400 MHz, $CDCl_3$): δ 7.33-7.21 (m, 6H), 7.12 (s, 2H), 5.48 (d, *J*=10.8 Hz, 2H), 5.24 (br, 2H), 2.36 (s, 6H), 1.44 (s, 18H); ^{13}C NMR (100 MHz, $CDCl_3$): δ 154.1, 138.8, 134.7, 130.5, 129.0, 128.8, 124.4, 113.3, 81.3, 56.5, 51.6, 28.1, 21.4; IR (neat) 2979, 1703, 1689, 1489, 1366, 1246, 1160, 1022, 752 cm^{-1}; $[\alpha]_D$ $^{23.8}$= +41.4° (c=1.0, $CHCl_3$, 99% ee).

Enantiomeric excess was determined by HPLC with a Chiralpak IA column (hexane:2-propanol = 95:5, 0.5 mL/min, 254 nm) ; major enantiomer t_r = 18.0 min, minor enantiomer t_r = 25.2 min, 99% ee; HRMS (ESI+) calcd for $C_{29}H_{37}O_4$ N_4 $(M+H)^+$: 505.2809, found: 505.2806.

di-*tert*-butyl (*(1S,3S)*-2,2-dicyano-1,3-di-*p*-tolylpropane-1,3-diyl)dicarbamate (3d)

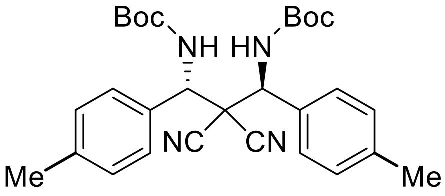

^{1}H NMR (400 MHz, $CDCl_3$): δ 7.30 (d, *J*=8.4 Hz, 2H), 7.21 (d, *J*=8.0 Hz, 2H), 5.45 (d, *J*=10.4, 2H), 5.24 (br, 2H), 2.37 (s, 6H), 1.43 (s, 18H); ^{13}C NMR (100 MHz, $CDCl_3$): δ 154.1, 139.7, 131.9, 129.8, 127.6, 113.4, 81.3, 56.3, 51.9, 28.2, 21.2; IR (neat) 2974, 1701, 1510, 1366, 1245, 1162, 1019, 755 cm^{-1}; $[\alpha]_D$ $^{23.9}$= +30.2° (c=1.0, $CHCl_3$, 99% ee).

Enantiomeric excess was determined by HPLC with a Chiralpak OD-H column (hexane:2-propanol = 70:30, 0.5 mL/min, 254 nm) ; major enantiomer t_r = 9.9 min, minor enantiomer t_r = 18.3 min, 99% ee; HRMS (ESI+) calcd for $C_{29}H_{37}O_4$ N_4 $(M+H)^+$: 505.2809, found: 505.2805.

di-*tert*-butyl (*(1R,3R)*-1,3-bis(2-bromophenyl)-2,2-dicyanopropane-1,3-diyl)dicarbamate (3e)

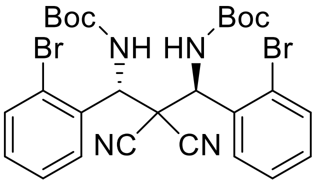

^{1}H NMR (400 MHz, $CDCl_3$): δ 7.68-7.63 (m, 4H), 7.46-7.42(m, 2H), 7.31-7.27 (m, 2H), 6.16 (br, 2H), 5.54 (br, 2H), 1.46 (s, 18H); ^{13}C NMR (100 MHz, $CDCl_3$): δ 153.8, 135.3, 133.6, 131.0, 128.5, 127.6, 124.9, 111.7, 81.6, 54.7, 51.0 28.2; IR (neat) 1717, 1508, 1473, 1368, 1246, 1163, 752 cm^{-1}; $[\alpha]_D$ $^{20.2}$= +43.5 (c=1.0, $CHCl_3$,96% ee). Enantiomeric excess was determined by HPLC with a Chiralpak AD-H column (hexane:2-propanol = 90:10, 1.0 mL/min, 254 nm) ; major enantiomer t_r =10.3 min, minor enantiomer t_r = 30.7 min, 96% ee; HRMS (ESI+) calcd for $C_{27}H_{31}O_4N_4Br_2$ $(M+H)^+$: 633.0712, found: 633.0711.

di-*tert*-butyl (*(1S,3S)*-2,2-dicyano-1,3-bis(2-nitrophenyl)propane-1,3-diyl)dicarbamate (3f)

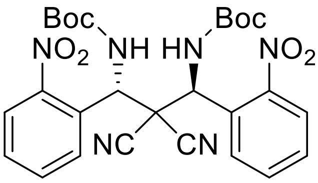

^{1}H NMR (400 MHz, $CDCl_3$): δ 8.24 (d, *J* = 8.15 Hz, 2H), 7.85-7.77 (m, 4H), 7.65-7.61 (m, 2H), 6.85 (br, 2H), 5.84 (br, 2H), 1.47(s, 18H) ^{13}C NMR (100 MHz, $CDCl_3$): δ 153.9, 147.8, 134.6, 131.6, 130.6, 128.7, 126.0, 111.6, 82.0, 50.8, ,50.4, 28.1; IR (neat) 1717, 1532, 1350, 1248, 1159, 759 cm^{-1}; $[\alpha]_D$ $^{20.2}$= -31.6° (c=1.0, $CHCl_3$, 87% ee).

Enantiomeric excess was determined by HPLC with a Chiralpak IA column (hexane:2-propanol = 70:30, 1.0 mL/min, 254 nm) ; major enantiomer t_r = 22.3 min, minor enantiomer t_r = 11.0 min, 87% ee; HRMS (ESI+) calcd for $C_{27}H_{31}O_8N_6$ $(M+H)^+$: 567.2203, found: 567.2199.

di-tert-butyl (*(1S,3S)*-2,2-dicyano-1,3-bis(2-(trifluoromethyl)phenyl)propane-1,3-diyl)dicarbamate (3g)

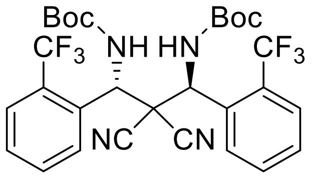

^{1}H NMR (400 MHz, $CDCl_3$): δ 7.91 (d, *J*=8.0 Hz, 2H), 7.77 (d, *J*=8.0 Hz, 2H), 7.68 (t, *J*=7.8 Hz, 2H), 7.55 (t, *J*=7.8 Hz, 2H), 5.95 (d, *J*=10.0 Hz, 2H), 5.51 (br, 2H), 1.47 (s, 18H); ^{13}C NMR (100 MHz, $CDCl_3$): δ 153.5, 135.1, 133.0, 130.0, 128.6 (d, $^2J_{CF}$=30.5 Hz), 127.5, 126.8 (d, $^3J_{CF}$=5.7 Hz), 123.7 (d, $^1J_{CF}$=279.5 Hz), 112.4, 81.7, 52.2, 51.8, 28.0; IR (neat) 2981, 1705, 1491, 1367, 1311, 1158, 1125, 1038, 768 cm^{-1}; $[\alpha]_D^{20.6}$= +65.2° (c=1.0, $CHCl_3$, 88% ee).

Enantiomeric excess was determined by HPLC with a Chiralpak IA column (hexane:2-propanol = 95:5, 0.5 mL/min, 254 nm) ; major enantiomer t_r = 43.3 min, minor enantiomer t_r = 58.0 min, 88% ee; HRMS (ESI+) calcd for $C_{29}H_{31}F_6O_4N_4$ $(M+H)^+$: 613.2244, found: 613.2241.

di-*tert*-butyl (*(1S,3S)*-2,2-dicyano-1,3-bis(3-methoxyphenyl)propane-1,3-diyl)dicarbamate (3h)

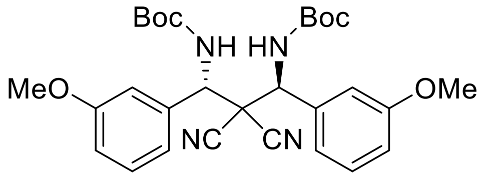

^{1}H NMR (400 MHz, $CDCl_3$): δ 7.33 (t, *J*=8.0 Hz, 2H), 7.04 (d, *J*=7.9 Hz, 2H), 6.94 (dd, *J*=8.3, 1.9 Hz, 2H), 6.88 (t, *J*=1.8 Hz, 2H), 5.48 (d, *J*=10.0 Hz, 2H), 5.27 (br, 2H), 3.80 (s, 6H), 1.44 (s, 18H); ^{13}C NMR (100 MHz, $CDCl_3$): δ 159.8, 154.1, 136.2, 130.3, 119.6, 115.5, 113.4, 113.3, 81.4, 56.6, 55.3, 51.5, 28.2; IR (neat) 2978, 1705, 1521, 1496, 1368, 1252, 1164, 1049, 758 cm^{-1}; $[\alpha]_D^{20.2}$= +20.6° (c=1.0, $CHCl_3$, 90% ee).

Enantiomeric excess was determined by HPLC with a Chiralpak AD-H column (hexane:2-propanol = 85:15, 1.0 mL/min, 254 nm) ; major enantiomer t_r = 7.1 min, minor enantiomer t_r = 19.0 min, 90% ee; HRMS (ESI+) calcd for $C_{29}H_{37}O_6N_4$ $(M+H)^+$: 537.2713, found: 537.2707.

di-*tert*-butyl (*(1R,3R)*-2,2-dicyano-1,3-di(furan-2-yl)propane-1,3-diyl)dicarbamate (3i)

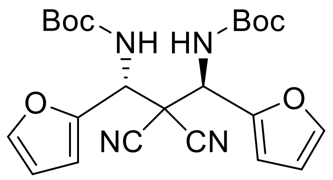

^{1}H NMR (400 MHz, $CDCl_3$): δ 7.50 (d, *J*=1.1 Hz, 2H), 6.58 (br, 2H), 6.43-6.41 (m, 2H), 5.54 (d, *J*=10.0 Hz, 2H), 5.43 (d, *J*=10.2 Hz, 2H) 1.48 (s, 18H); ^{13}C NMR (100 MHz, $CDCl_3$): δ 154.0, 146.8, 144.0, 112.4, 110.8, 110.6, 81.6, 51.2, 50.1, 28.1; IR (neat) 3304, 1706, 1528, 1368, 1248, 1162, 750cm^{-1}; $[\alpha]_D^{20.2}$= +12.0° (c=1.0, $CHCl_3$, 86% ee).

Enantiomeric excess was determined by HPLC with a Chiralpak IA column (hexane:2-propanol = 90:10, 1.0 mL/min, 254 nm) ; major enantiomer t_r = 14.3 min, minor enantiomer t_r = 18.8 min, 86% ee; HRMS (ESI+) calcd for $C_{23}H_{29}N_4O_6$ $(M+H)^+$: 457.2087, found: 457.2084.

di-tert-butyl (*(1S,3S)*-2,2-dicyano-1,3-di(naphthalen-2-yl)propane-1,3-diyl)dicarbamate (3j)

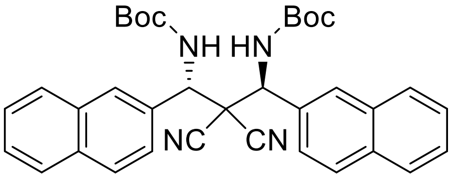

^{1}H NMR (400 MHz, $CDCl_3$): δ 7.93-7.79 (m, 8H), 7.60-7.50 (m, 6H), 5.65 (d, *J*=10.4 Hz, 2H), 5.50 (br, 2H), 1.44 (s, 18H); ^{13}C NMR (100 MHz, $CDCl_3$): δ 154.1, 133.6, 132.8, 131.9, 129.3, 128.4, 128.1, 127.7, 127.2, 126.9, 124.2, 133.4, 81.5, 57.1, 51.5, 28.2; IR (neat) 2978, 1700, 1507, 1367, 1245, 1159, 751 cm^{-1}; $[\alpha]_D$ $^{20.3}$= +26.6° (c=1.0, $CHCl_3$, 91% ee).

Enantiomeric excess was determined by HPLC with a Chiralpak AS-H column (hexane:2-propanol = 90:10, 1.0 mL/min, 254 nm) ; major enantiomer t_r = 13.9 min, minor enantiomer t_r = 29.2 min, 91% ee; HRMS (ESI+) calcd for $C_{35}H_{37}O_4$ N_4 $(M+H)^+$: 577.2809, found: 577.2808.

7. ^{1}H and ^{13}C spectra

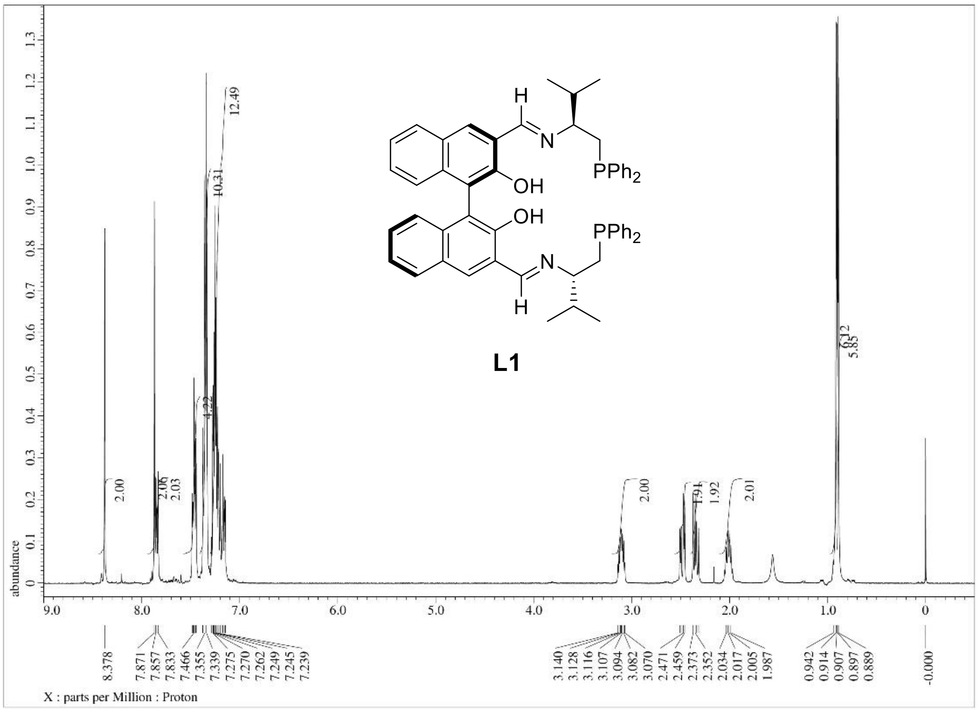

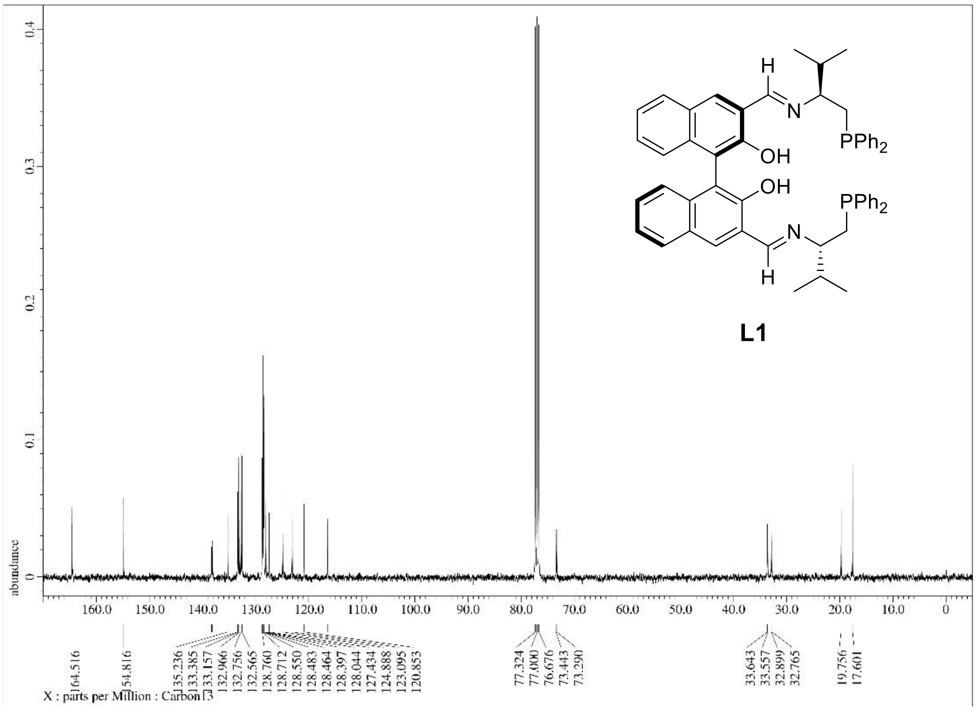

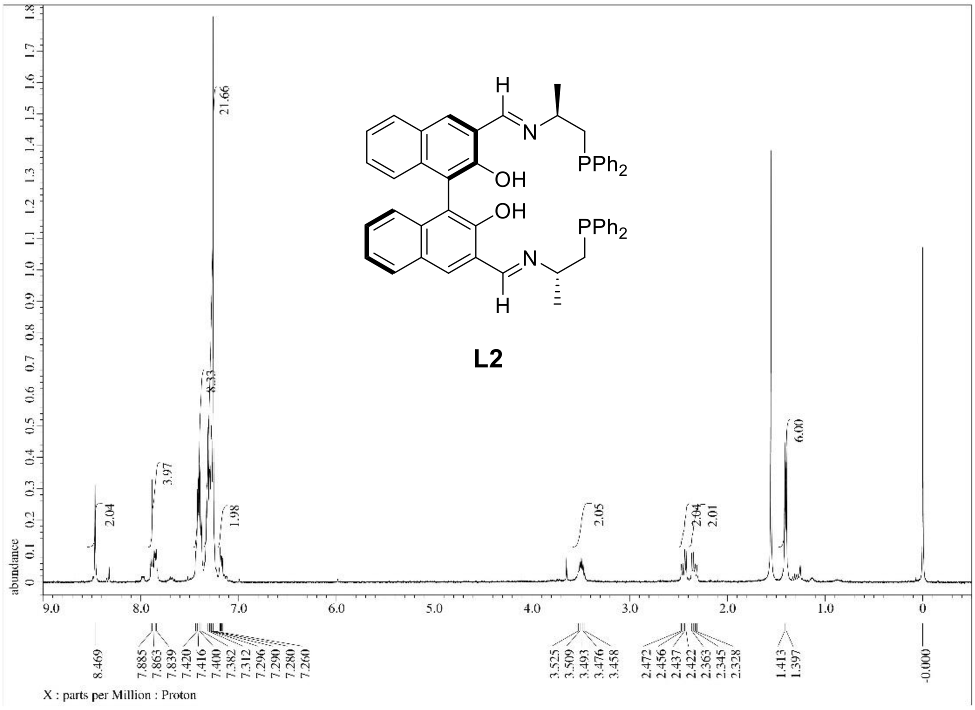
L2
X : parts per Million : Proton
abundance
8.469
7.885
7.863
7.839
7.420
7.416
7.400
7.382
7.312
7.296
7.290
7.280
7.260
3.525
3.509
3.493
3.476
3.458
2.472
2.456
2.437
2.422
2.363
2.345
2.328
1.413
1.397
-0.000
2.04
3.97
8.33
21.66
1.98
2.05
2.04
2.01
6.00

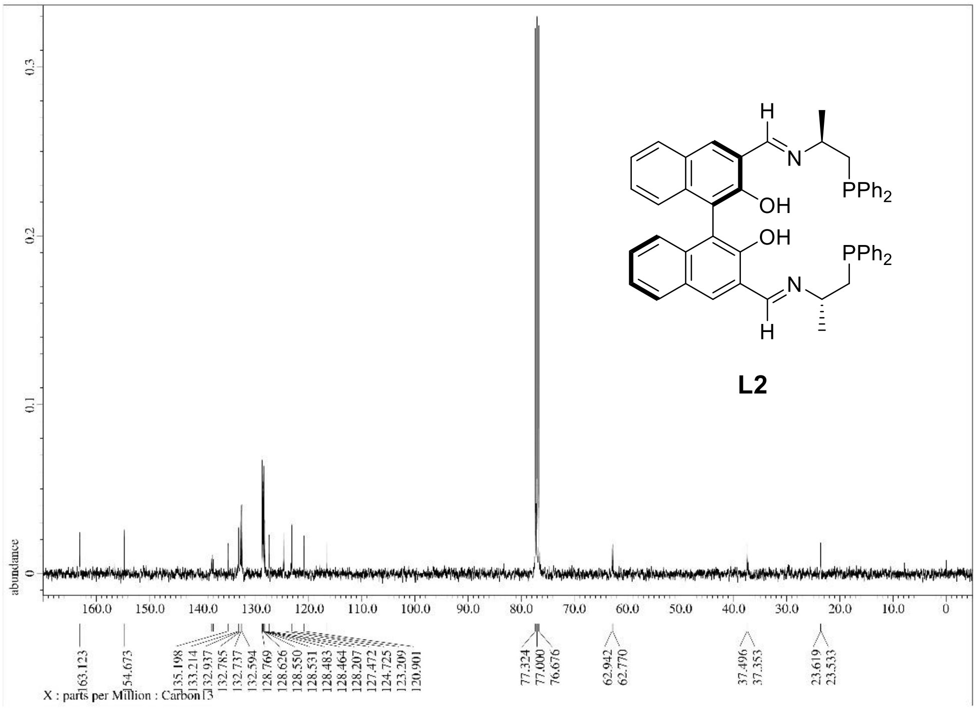
L2
X : parts per Million : Carbon13
abundance
163.123
154.673
135.198
133.214
132.937
132.785
132.737
132.594
128.769
128.626
128.550
128.531
128.483
128.464
128.207
127.472
124.725
123.209
120.901
77.324
77.000
76.676
62.942
62.770
37.496
37.353
23.619
23.533

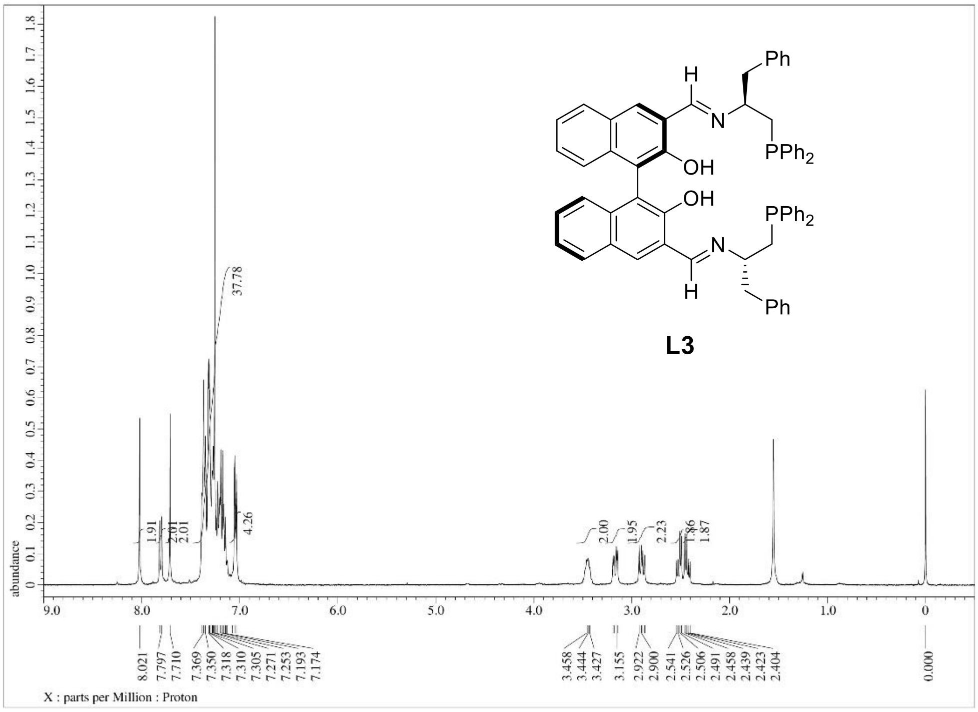
abundance
X : parts per Million : Proton
L3
OH
PPh2
Ph

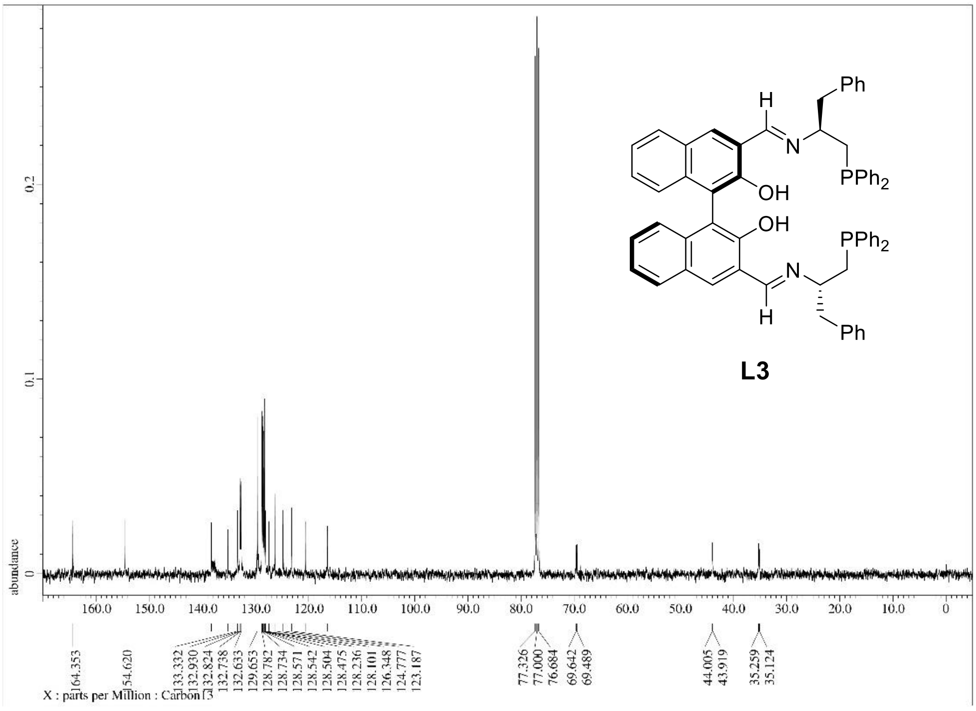
abundance
X : parts per Million : Carbon13
L3

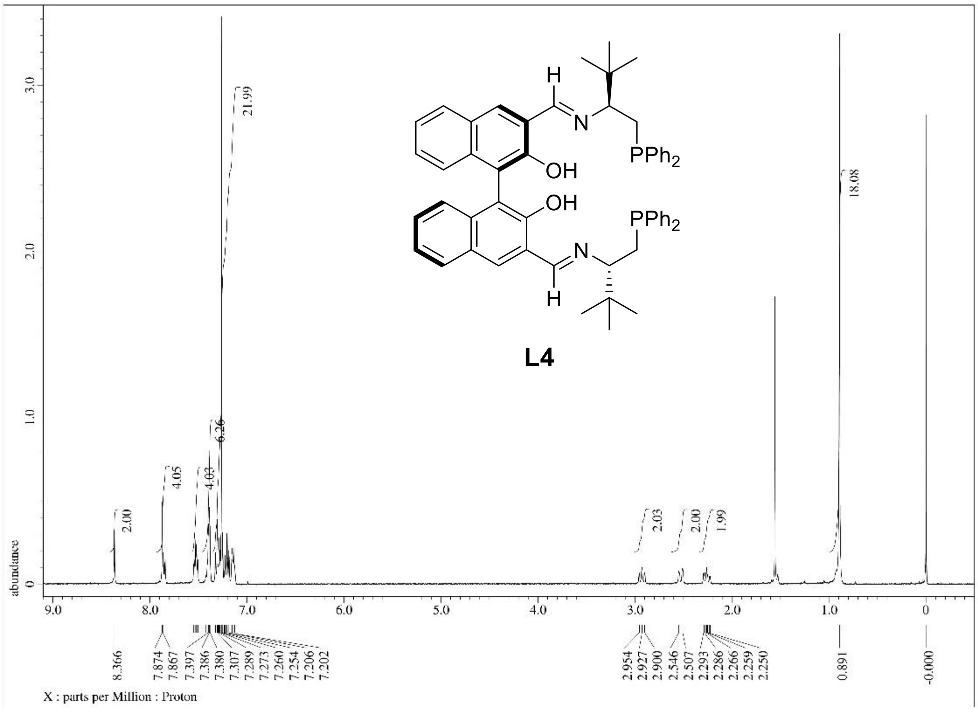

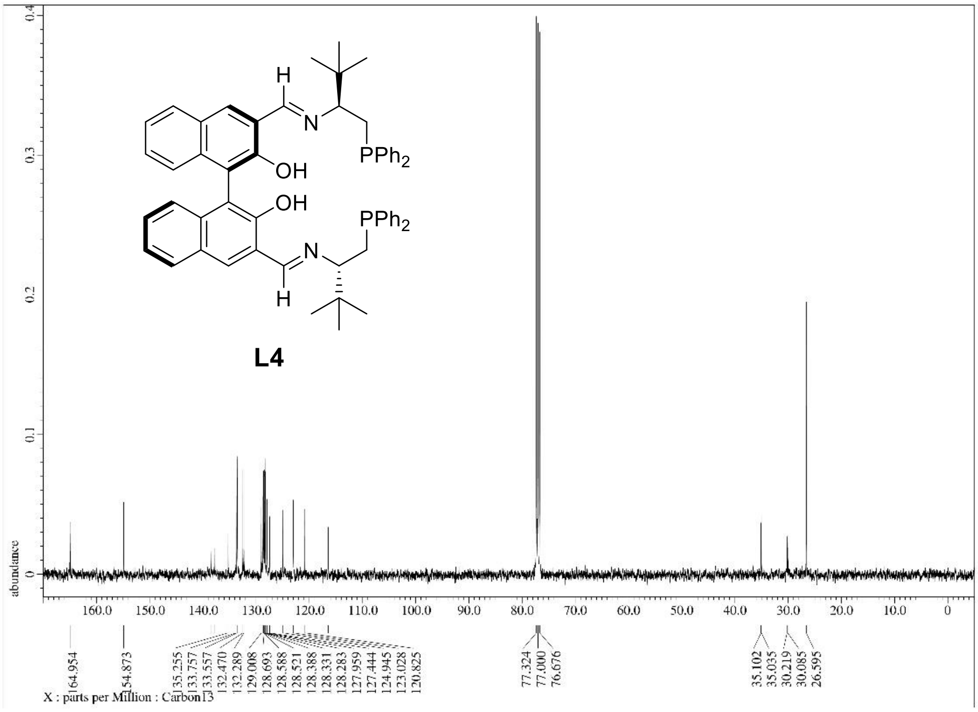

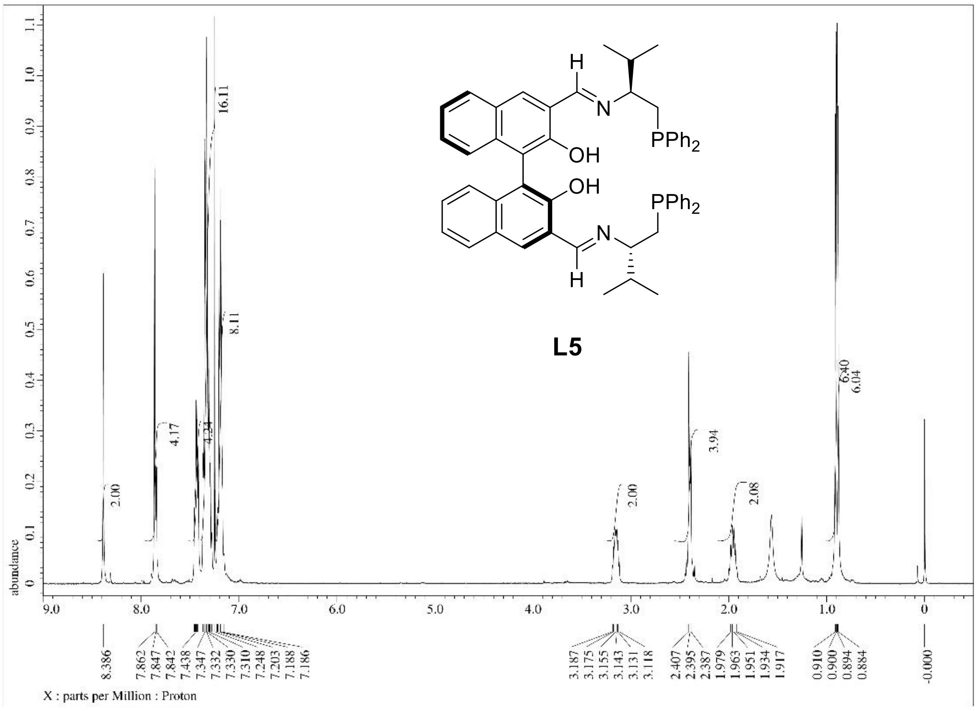
L5
X : parts per Million : Proton
abundance

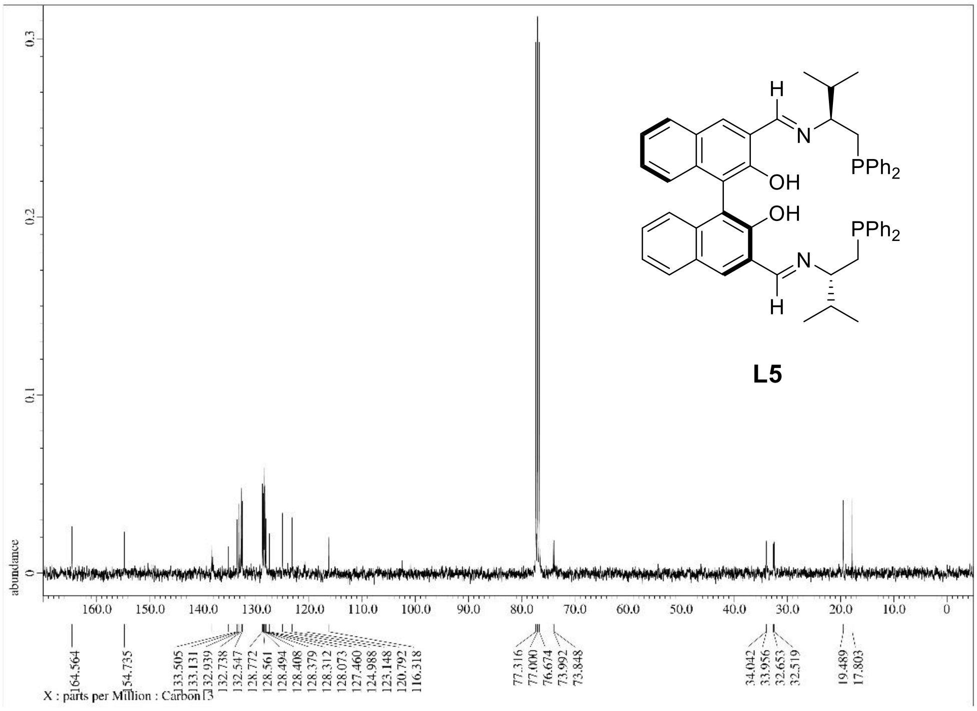
L5
X : parts per Million : Carbon13
abundance

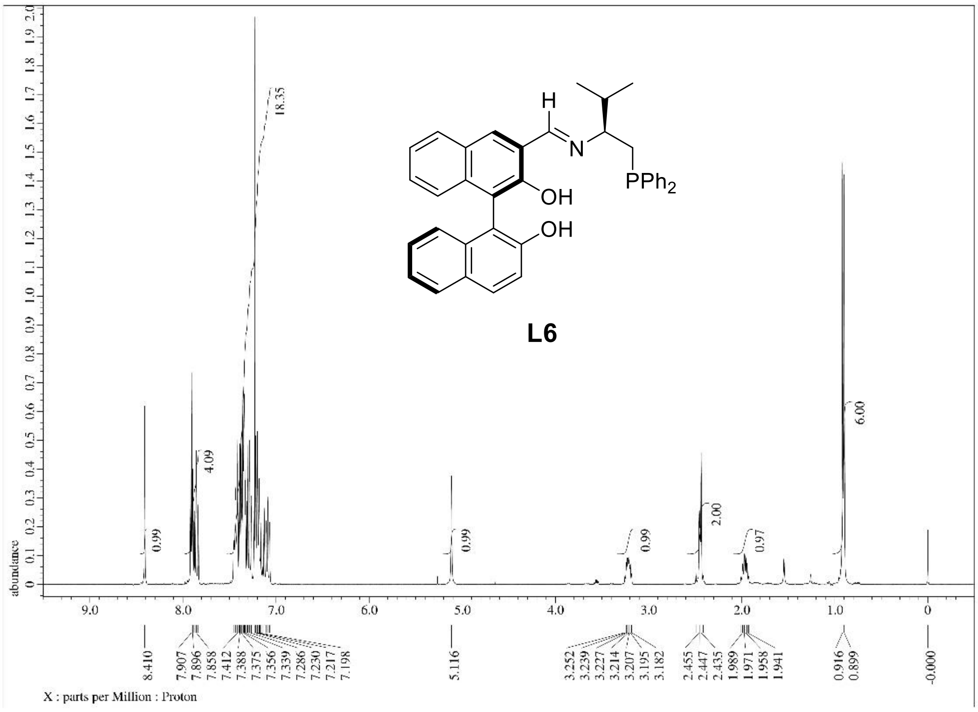
L6
OH
OH
N
H
PPh2
abundance
X : parts per Million : Proton
8.410
7.907
7.896
7.858
7.412
7.388
7.375
7.356
7.339
7.286
7.230
7.217
7.198
5.116
3.252
3.239
3.227
3.214
3.207
3.195
3.182
2.455
2.447
2.435
1.989
1.971
1.958
1.941
0.916
0.899
-0.000
0.99
4.09
18.35
0.99
0.99
2.00
0.97
6.00

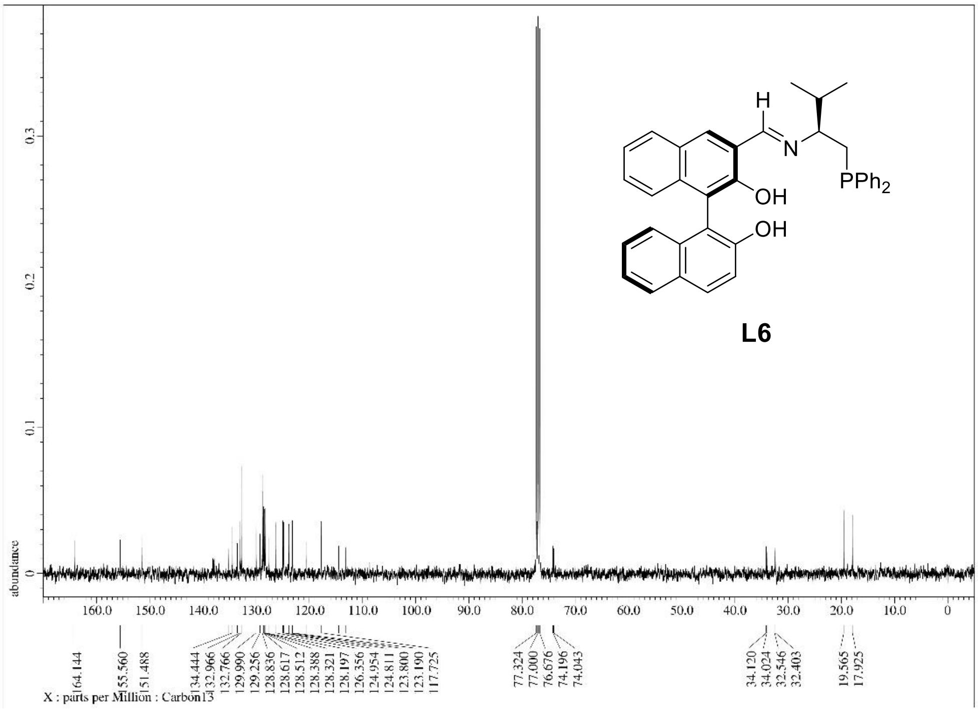
L6
OH
OH
N
H
PPh2
abundance
X : parts per Million : Carbon13
164.144
155.560
151.488
134.444
132.966
132.766
129.990
129.256
128.836
128.617
128.512
128.388
128.321
128.197
126.356
124.954
124.811
123.800
123.190
117.725
77.324
77.000
76.676
74.196
74.043
34.120
34.024
32.546
32.403
19.565
17.925

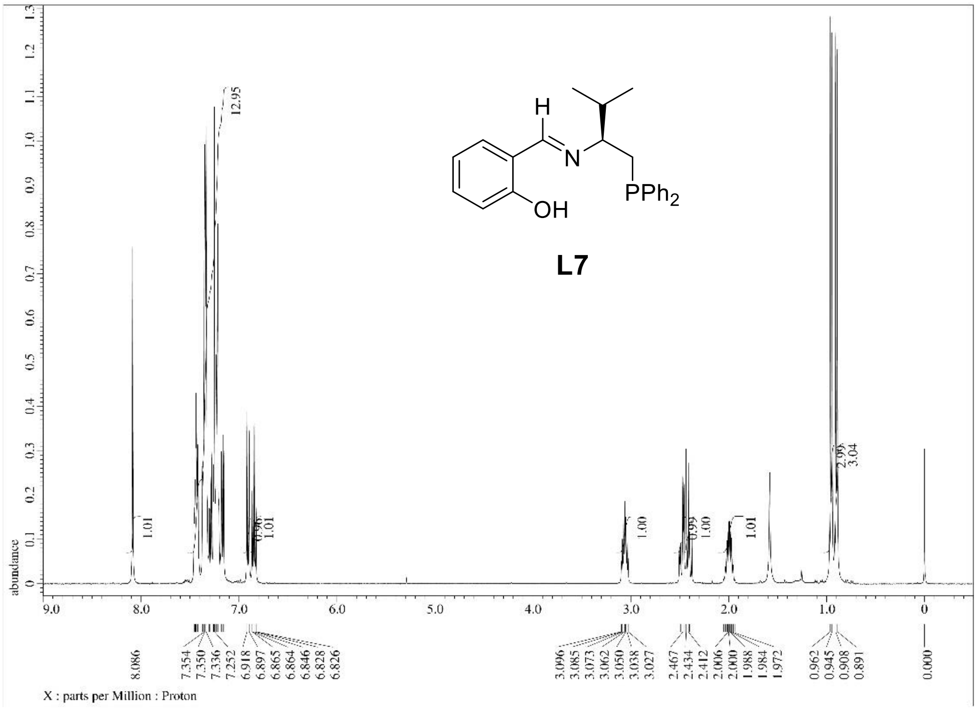
L7
PPh2
OH
X : parts per Million : Proton
abundance

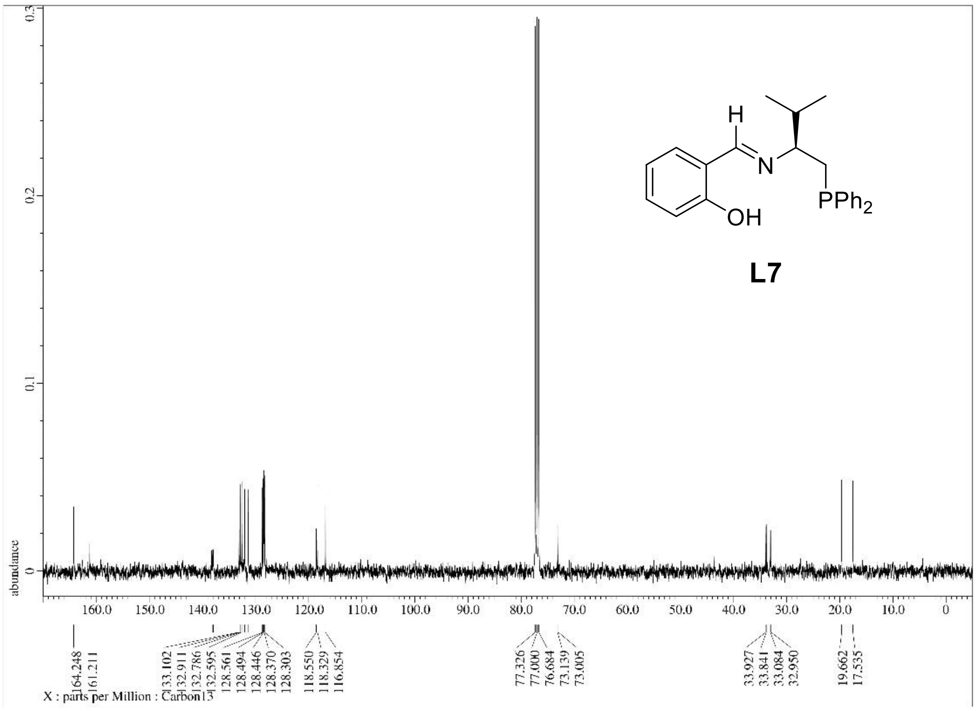
L7
PPh2
OH
X : parts per Million : Carbon13
abundance

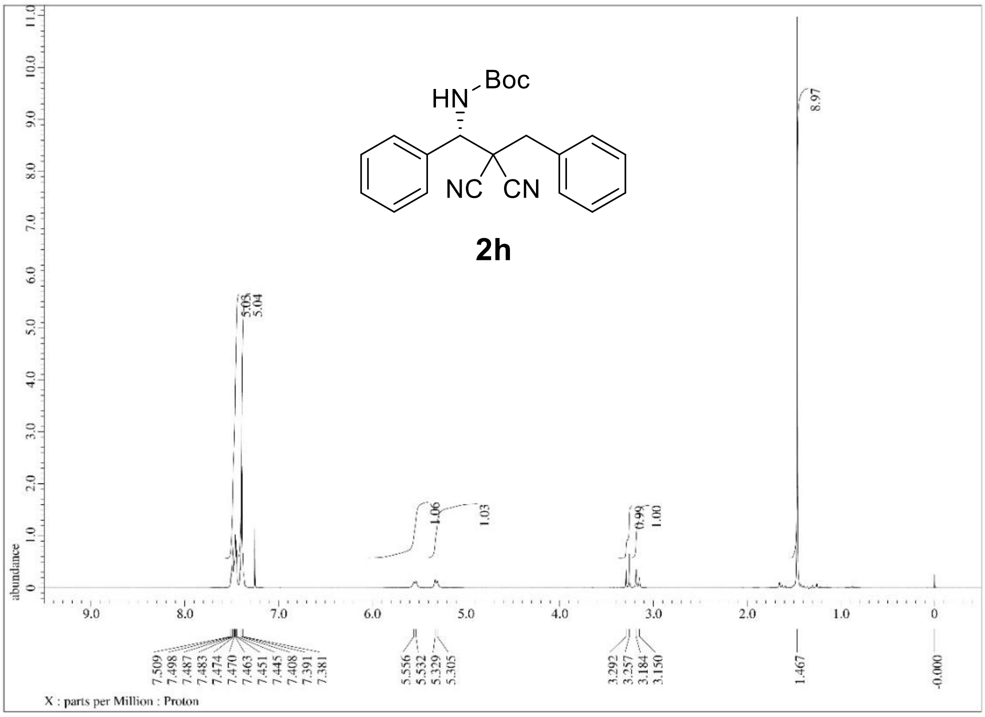
HN
Boc
NC
CN
2h
abundance
X : parts per Million : Proton
8.97
5.03
5.04
1.06
1.03
0.99
1.00
7.509
7.498
7.487
7.483
7.474
7.470
7.463
7.451
7.445
7.408
7.391
7.381
5.556
5.532
5.329
5.305
3.292
3.257
3.184
3.150
1.467
-0.000

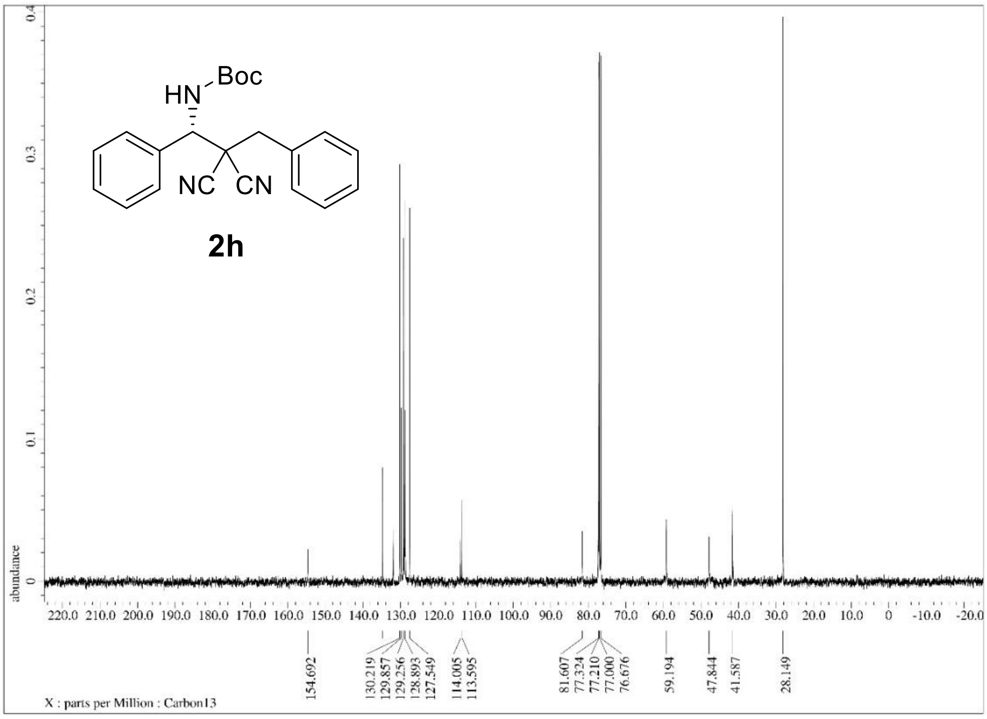
HN
Boc
NC
CN
2h
abundance
X : parts per Million : Carbon13
154.692
130.219
129.857
129.256
128.893
127.549
114.005
113.595
81.607
77.324
77.210
77.000
76.676
59.194
47.844
41.587
28.149

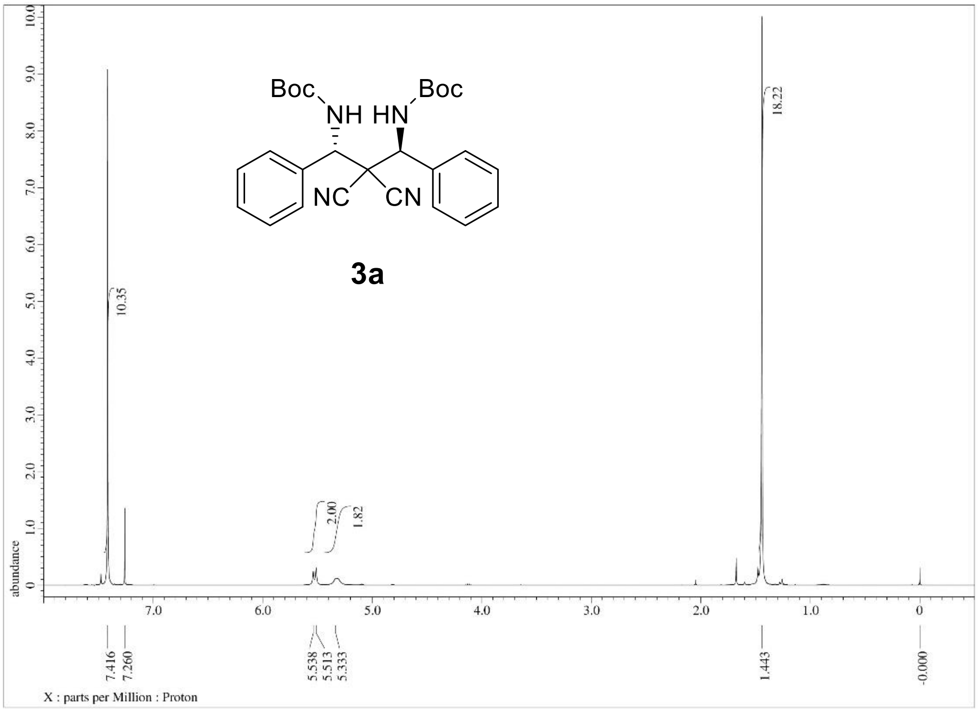
3a
10.35
2.00
1.82
18.22
7.416
7.260
5.538
5.513
5.333
1.443
-0.000
abundance
X : parts per Million : Proton

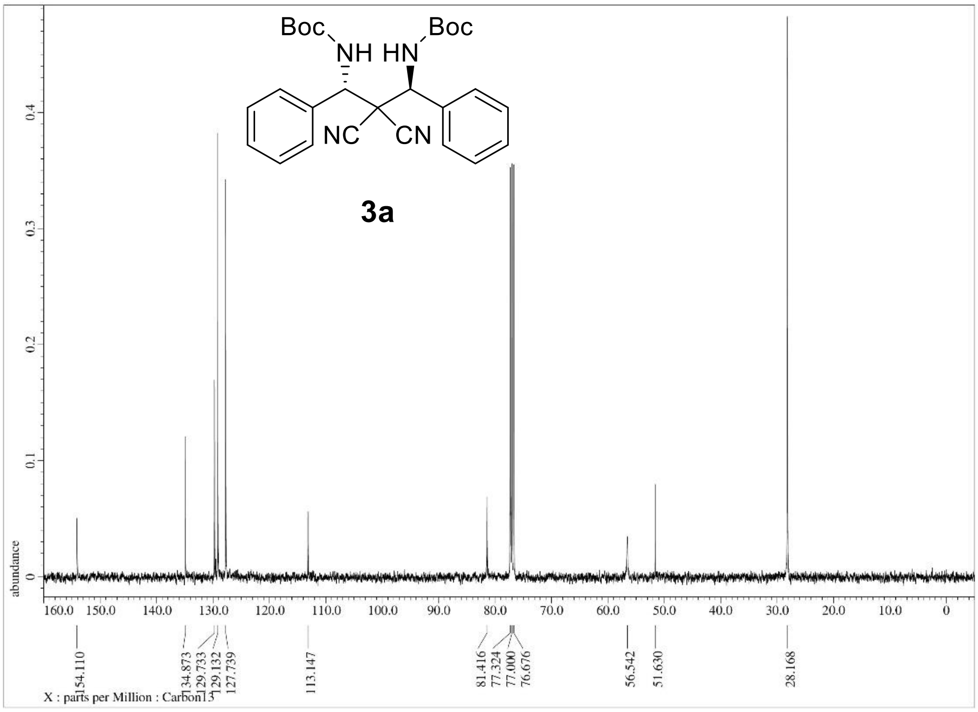
3a
154.110
134.873
129.733
129.132
127.739
113.147
81.416
77.324
77.000
76.676
56.542
51.630
28.168
abundance
X : parts per Million : Carbon13

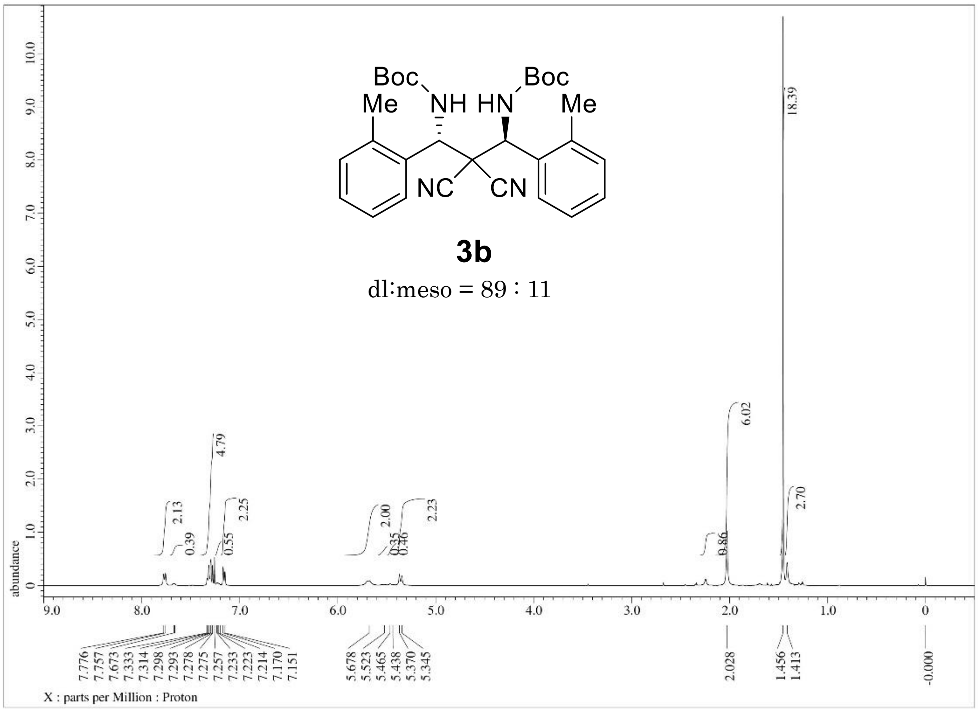
Boc
Me
NH
HN
Boc
Me
NC
CN
3b
dl:meso = 89 : 11
abundance
X : parts per Million : Proton
18.39
6.02
4.79
2.70
2.25
2.23
2.13
2.00
0.86
0.55
0.39
7.776
7.757
7.673
7.333
7.314
7.298
7.293
7.278
7.275
7.257
7.233
7.223
7.214
7.170
7.151
5.678
5.523
5.465
5.438
5.370
5.345
2.028
1.456
1.413
-0.000

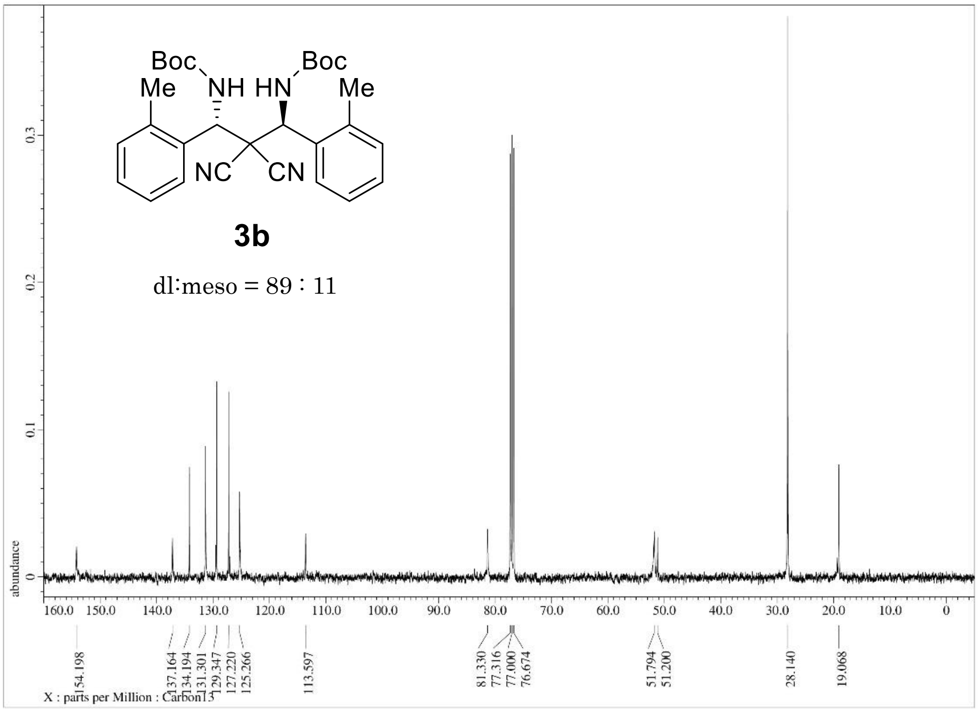
Boc
Me
NH
HN
Boc
Me
NC
CN
3b
dl:meso = 89 : 11
abundance
X : parts per Million : Carbon13
154.198
137.164
134.194
131.301
129.347
127.220
125.266
113.597
81.330
77.316
77.000
76.674
51.794
51.200
28.140
19.068

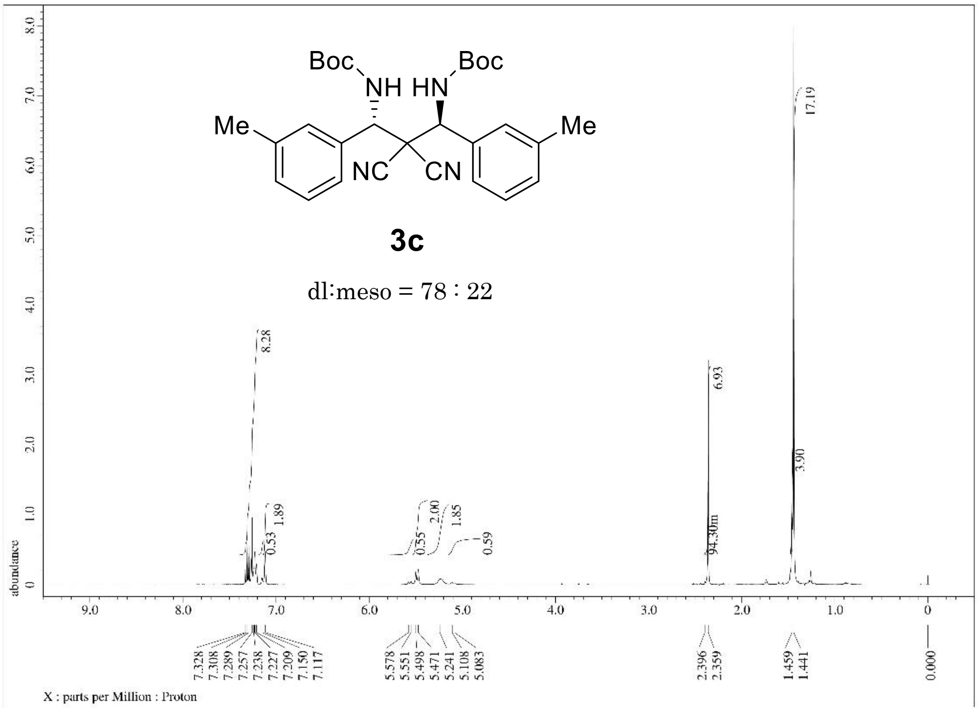
Boc
NH HN
Boc
Me
Me
NC
CN
3c
dl:meso = 78 : 22
abundance
8.28
1.89
0.53
0.55
2.00
1.85
0.59
6.93
94.30m
3.90
17.19
7.328
7.308
7.289
7.257
7.238
7.227
7.209
7.150
7.117
5.578
5.551
5.498
5.471
5.241
5.108
5.083
2.396
2.359
1.459
1.441
0.000
X : parts per Million : Proton

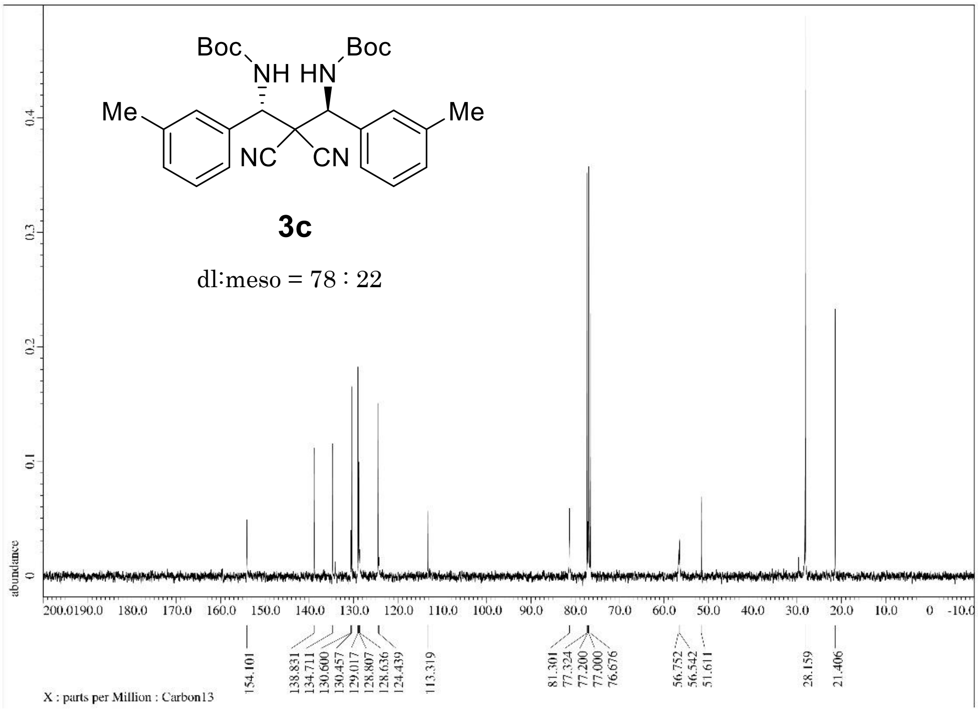
Boc
NH HN
Boc
Me
Me
NC
CN
3c
dl:meso = 78 : 22
abundance
154.101
138.831
134.711
130.600
130.457
129.017
128.807
128.636
124.439
113.319
81.301
77.324
77.200
77.000
76.676
56.752
56.542
51.611
28.159
21.406
X : parts per Million : Carbon13

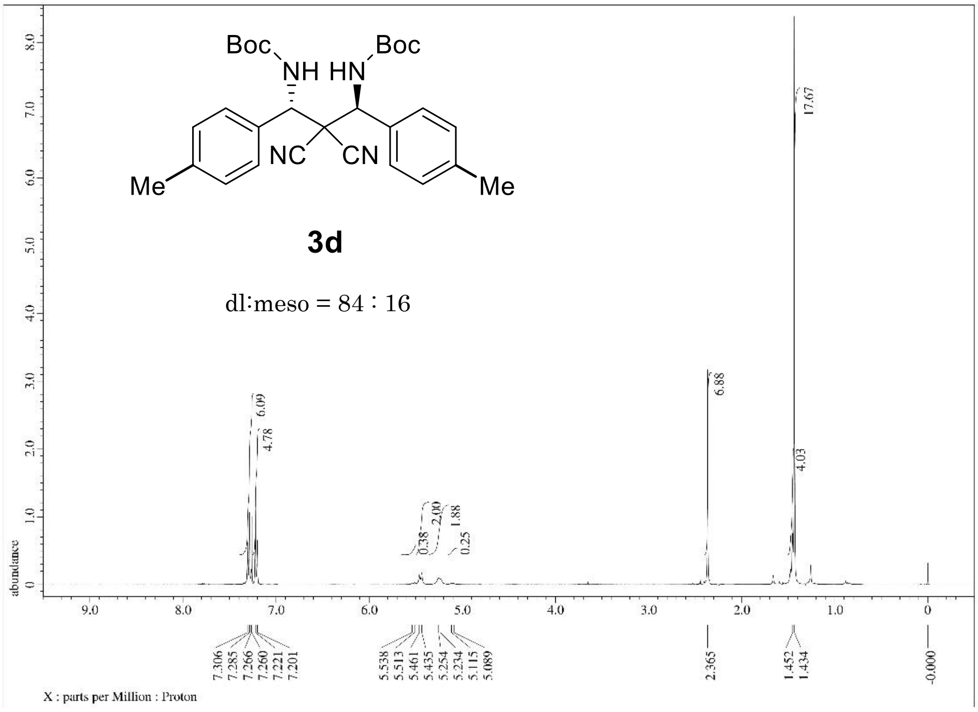
Boc
NH
HN
Boc
NC
CN
Me
Me
3d
dl:meso = 84 : 16
abundance
X : parts per Million : Proton
7.306
7.285
7.266
7.260
7.221
7.201
5.538
5.513
5.461
5.435
5.254
5.234
5.115
5.089
2.365
1.452
1.434
-0.000
6.09
4.78
0.38
2.00
1.88
0.25
6.88
17.67
4.03

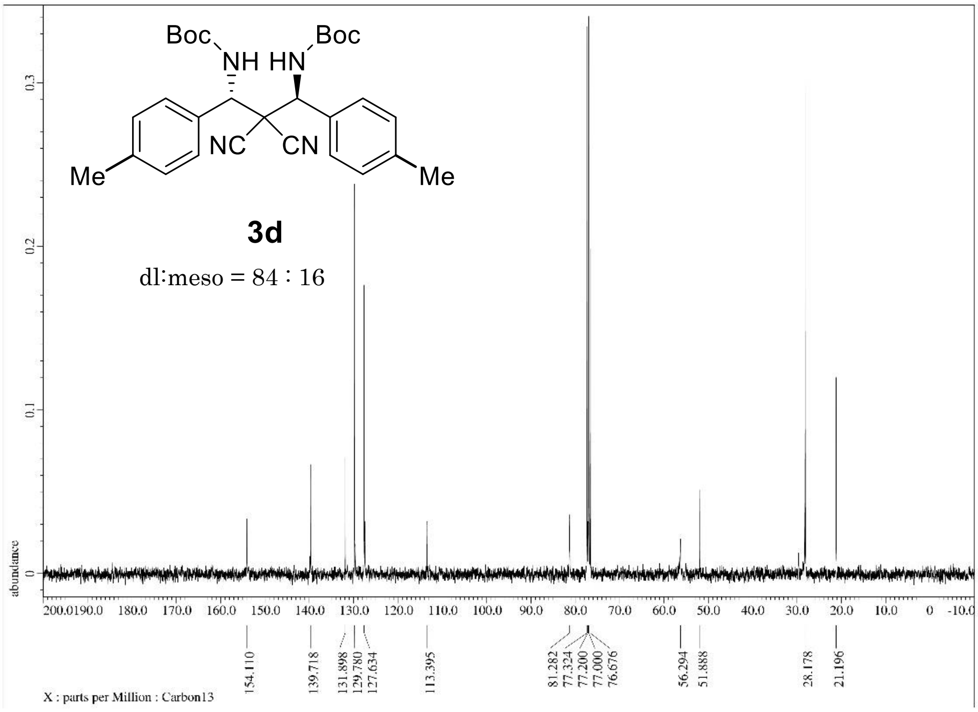
Boc
NH
HN
Boc
NC
CN
Me
Me
3d
dl:meso = 84 : 16
abundance
X : parts per Million : Carbon13
154.110
139.718
131.898
129.780
127.634
113.395
81.282
77.324
77.200
77.000
76.676
56.294
51.888
28.178
21.196

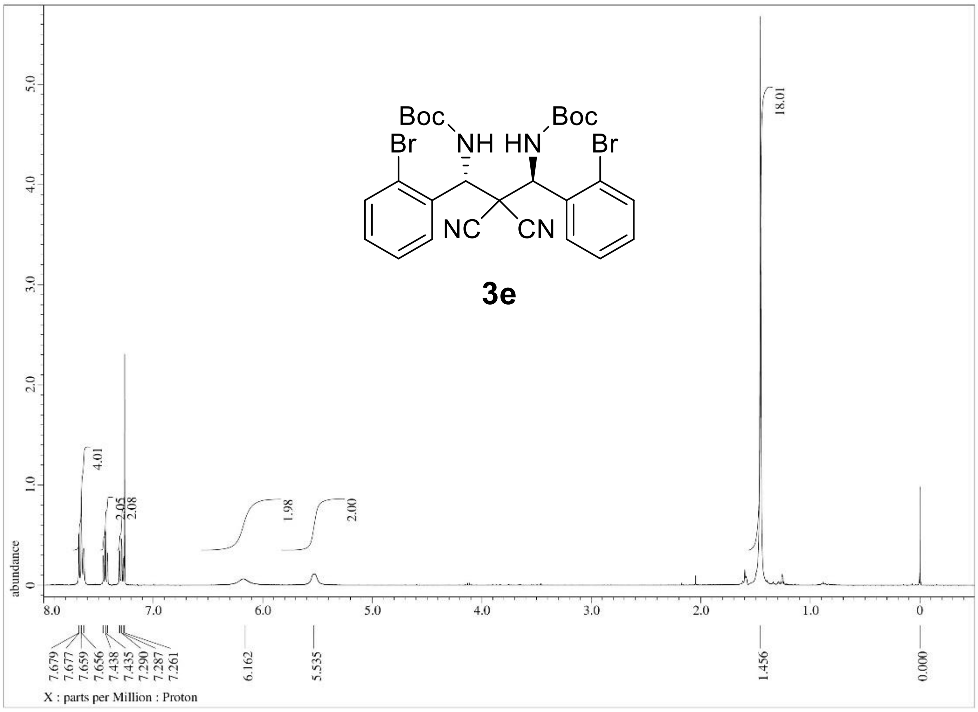
Boc
NH
HN
Boc
Br
Br
NC
CN
3e
18.01
4.01
2.05
2.08
1.98
2.00
abundance
7.679
7.677
7.659
7.656
7.438
7.435
7.290
7.287
7.261
6.162
5.535
1.456
0.000
X : parts per Million : Proton

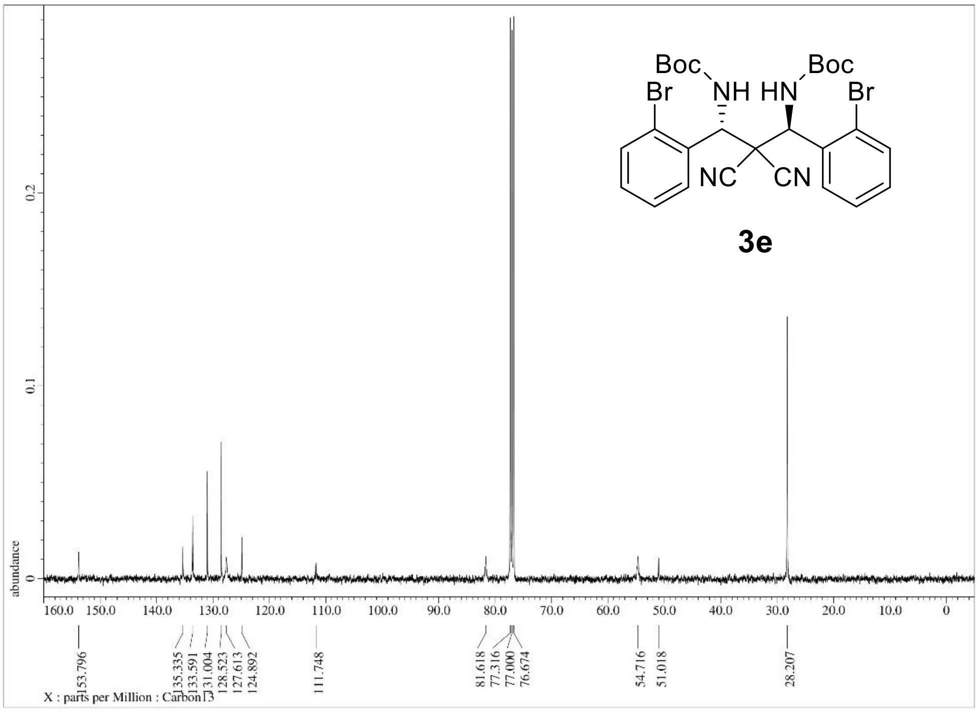
Boc
NH
HN
Boc
Br
Br
NC
CN
3e
abundance
153.796
135.335
133.591
131.004
128.523
127.613
124.892
111.748
81.618
77.316
77.000
76.674
54.716
51.018
28.207
X : parts per Million : Carbon13

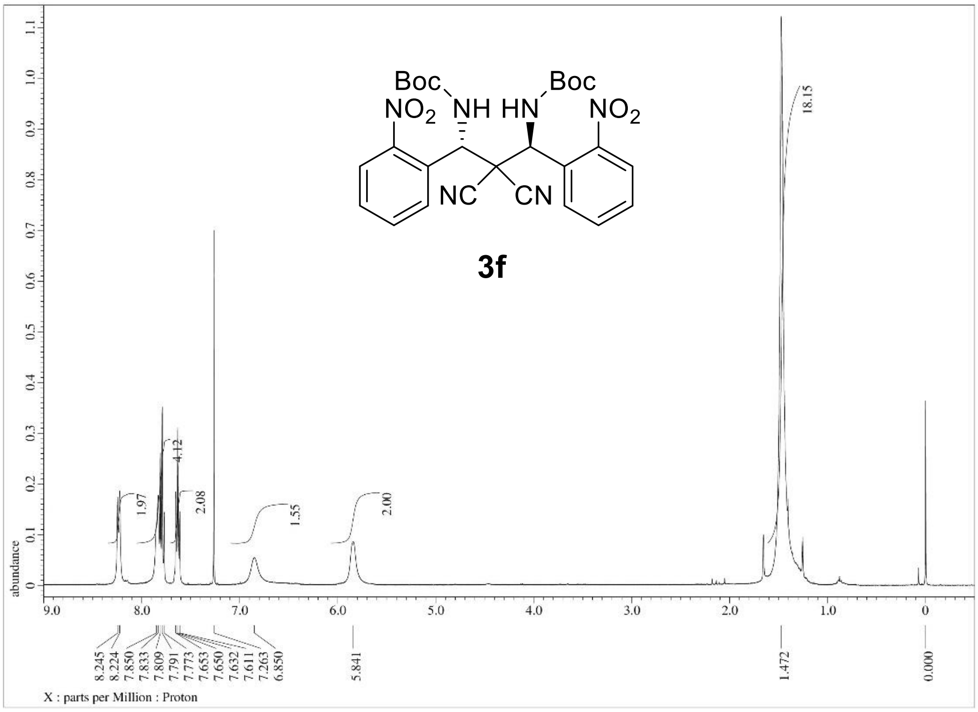
Boc
NO2
NH
HN
Boc
NO2
NC
CN
3f
1.97
4.12
2.08
1.55
2.00
18.15
abundance
8.245
8.224
7.850
7.833
7.809
7.791
7.773
7.653
7.650
7.632
7.611
7.263
6.850
5.841
1.472
0.000
X : parts per Million : Proton

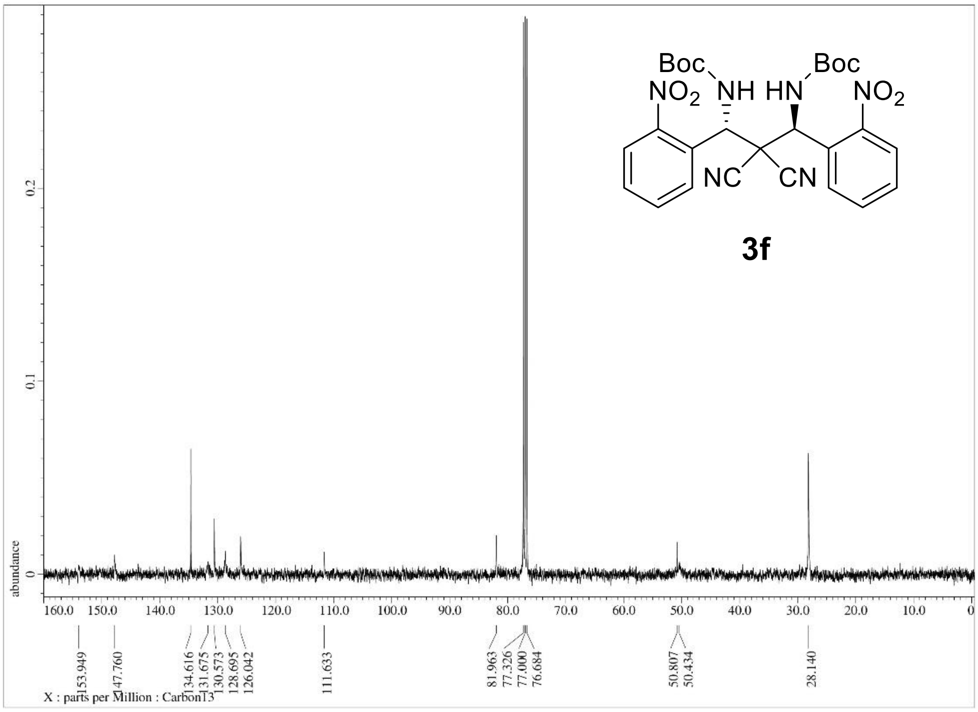
Boc
NO2
NH
HN
Boc
NO2
NC
CN
3f
abundance
153.949
147.760
134.616
131.675
130.573
128.695
126.042
111.633
81.963
77.326
77.000
76.684
50.807
50.434
28.140
X : parts per Million : Carbon13

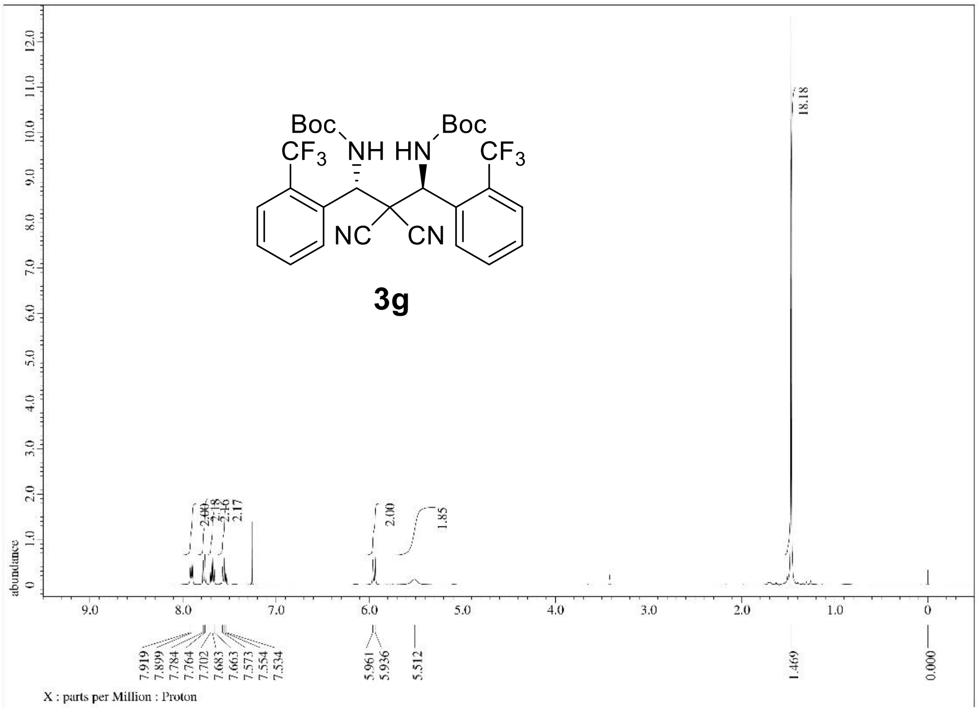
Boc
NH
HN
Boc
CF3
CF3
NC
CN
3g
18.18
2.00
1.85
7.919
7.899
7.784
7.764
7.702
7.683
7.663
7.573
7.554
7.534
5.961
5.936
5.512
1.469
0.000
abundance
X : parts per Million : Proton

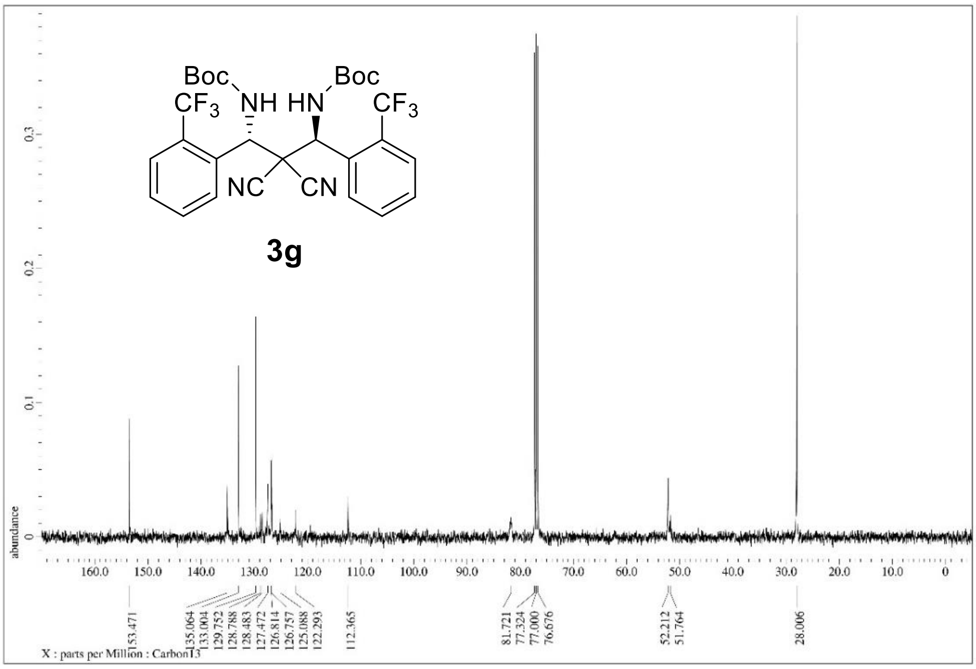
Boc
NH
HN
Boc
CF3
CF3
NC
CN
3g
153.471
135.064
133.004
129.752
128.788
128.483
127.472
126.814
126.757
125.088
122.293
112.365
81.721
77.324
77.000
76.676
52.212
51.764
28.006
abundance
X : parts per Million : Carbon13

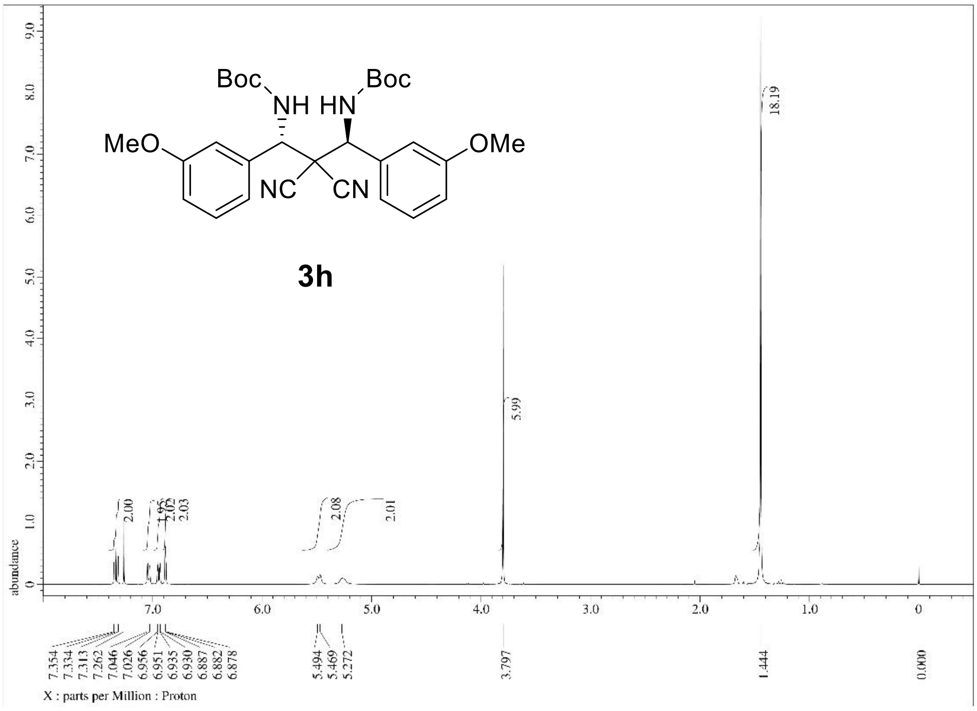
Boc
NH HN
Boc
MeO
OMe
NC
CN
3h
18.19
5.99
2.00
1.95
2.02
2.03
2.08
2.01
abundance
7.354
7.334
7.313
7.262
7.046
7.026
6.956
6.951
6.935
6.930
6.887
6.882
6.878
5.494
5.469
5.272
3.797
1.444
0.000
X : parts per Million : Proton

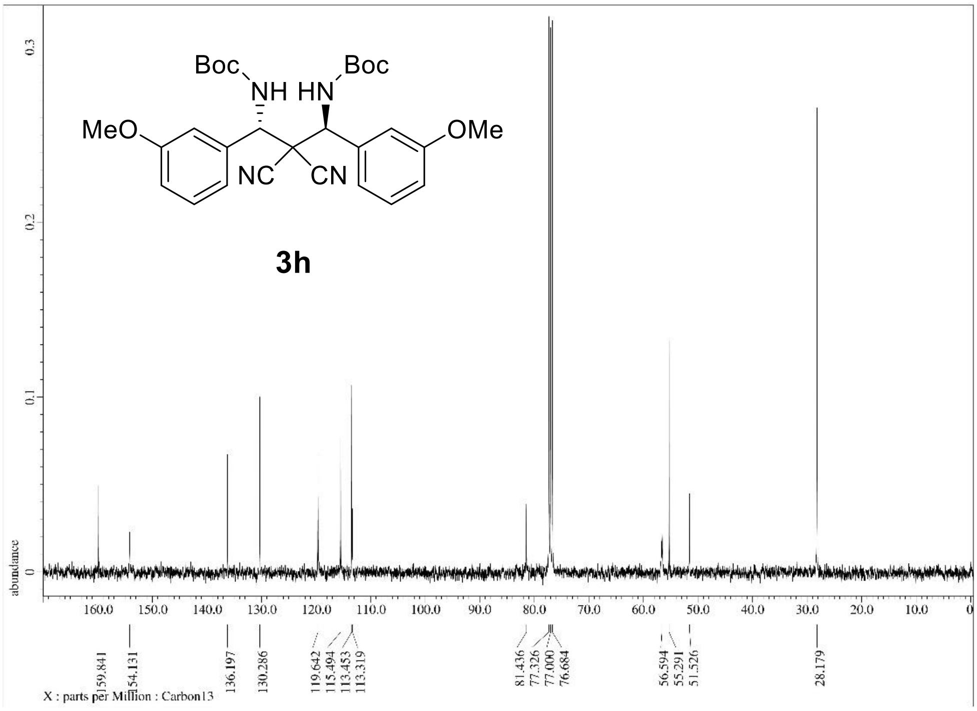
Boc
NH HN
Boc
MeO
OMe
NC
CN
3h
abundance
159.841
154.131
136.197
130.286
119.642
115.494
113.453
113.319
81.436
77.326
77.000
76.684
56.594
55.291
51.526
28.179
X : parts per Million : Carbon13

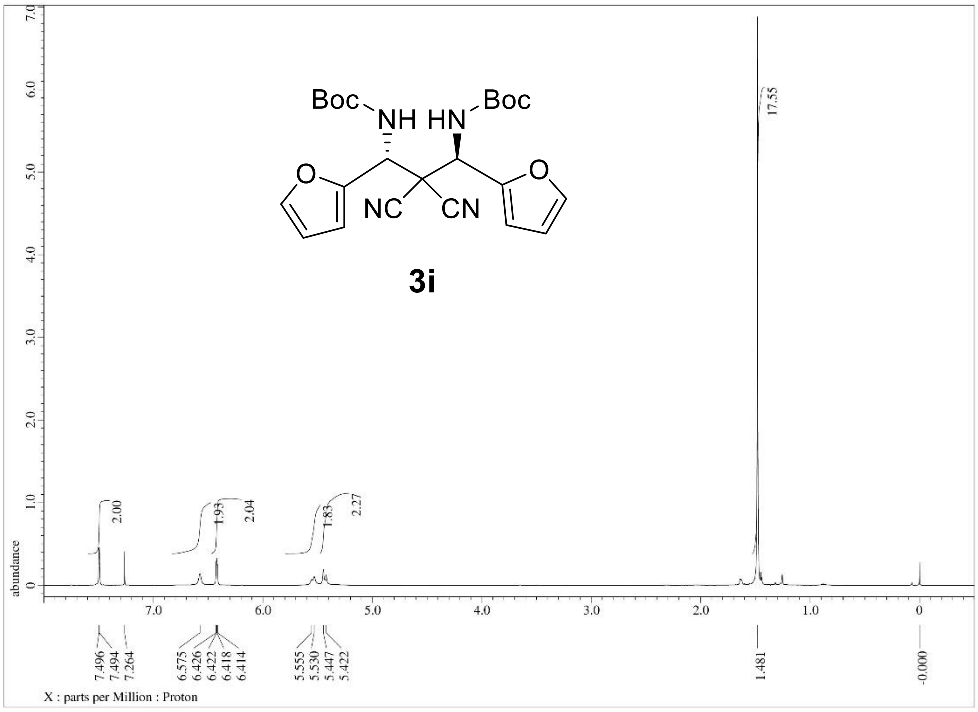
Boc
NH
HN
Boc
O
NC
CN
O
3i
17.55
2.00
1.93
2.04
1.83
2.27
abundance
7.0
6.0
5.0
4.0
3.0
2.0
1.0
0
7.496
7.494
7.264
6.575
6.426
6.422
6.418
6.414
5.555
5.530
5.447
5.422
1.481
-0.000
X : parts per Million : Proton

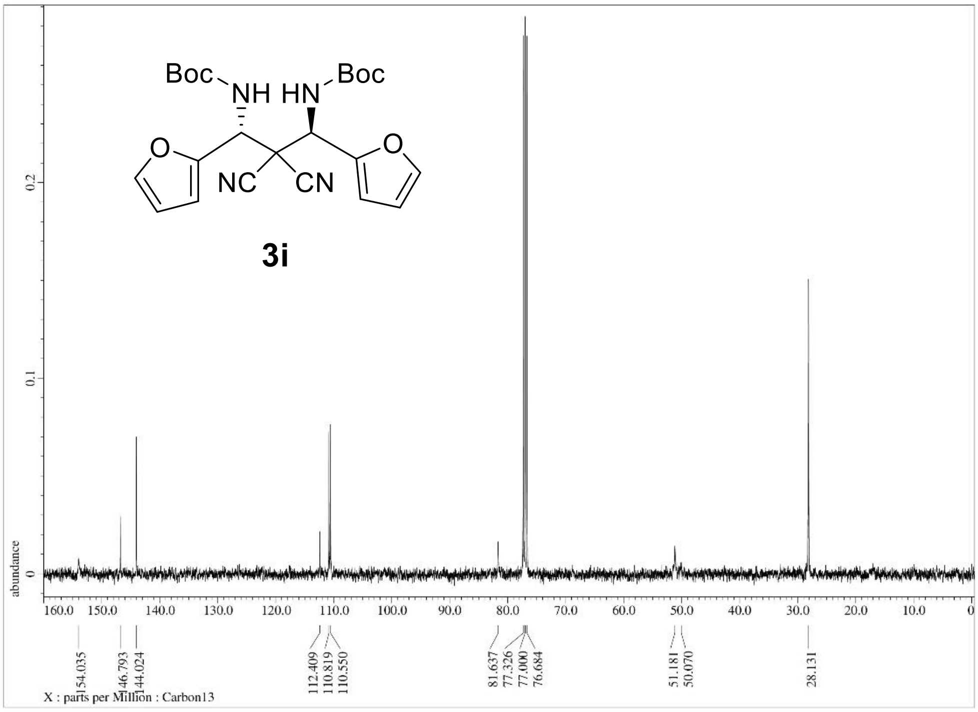
Boc
NH
HN
Boc
O
NC
CN
O
3i
abundance
0.2
0.1
0
160.0
150.0
140.0
130.0
120.0
110.0
100.0
90.0
80.0
70.0
60.0
50.0
40.0
30.0
20.0
10.0
154.035
146.793
144.024
112.409
110.819
110.550
81.637
77.326
77.000
76.684
51.181
50.070
28.131
X : parts per Million : Carbon13

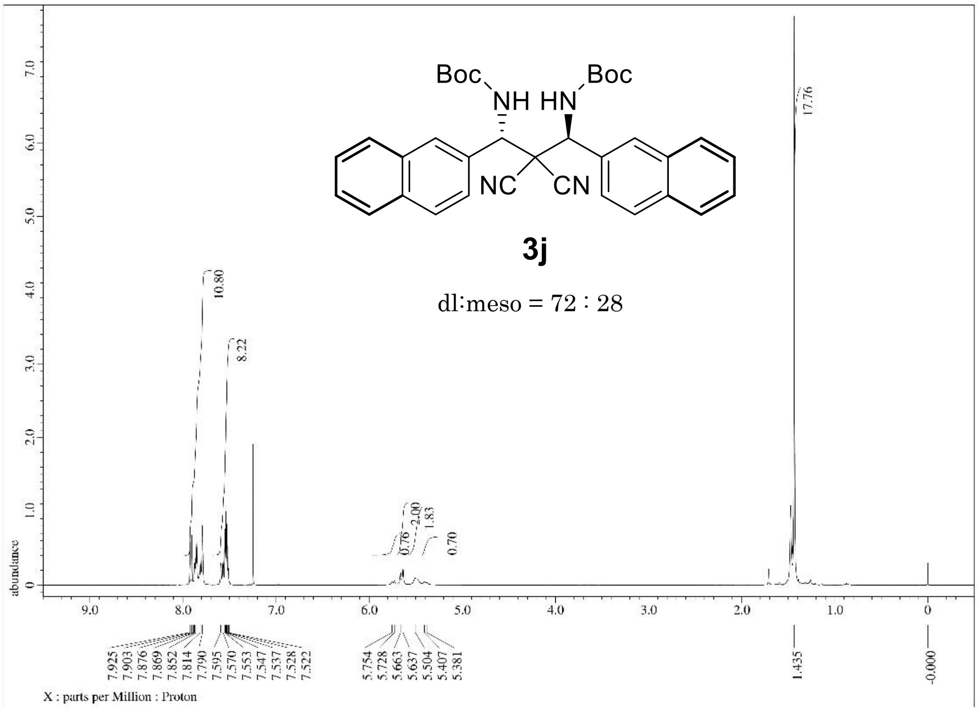
Boc
NH
HN
Boc
NC
CN
3j
dl:meso = 72 : 28
10.80
8.22
0.76
2.00
1.83
0.70
17.76
7.925
7.903
7.876
7.869
7.852
7.814
7.790
7.595
7.570
7.553
7.547
7.537
7.528
7.522
5.754
5.728
5.663
5.637
5.504
5.407
5.381
1.435
-0.000
abundance
X : parts per Million : Proton

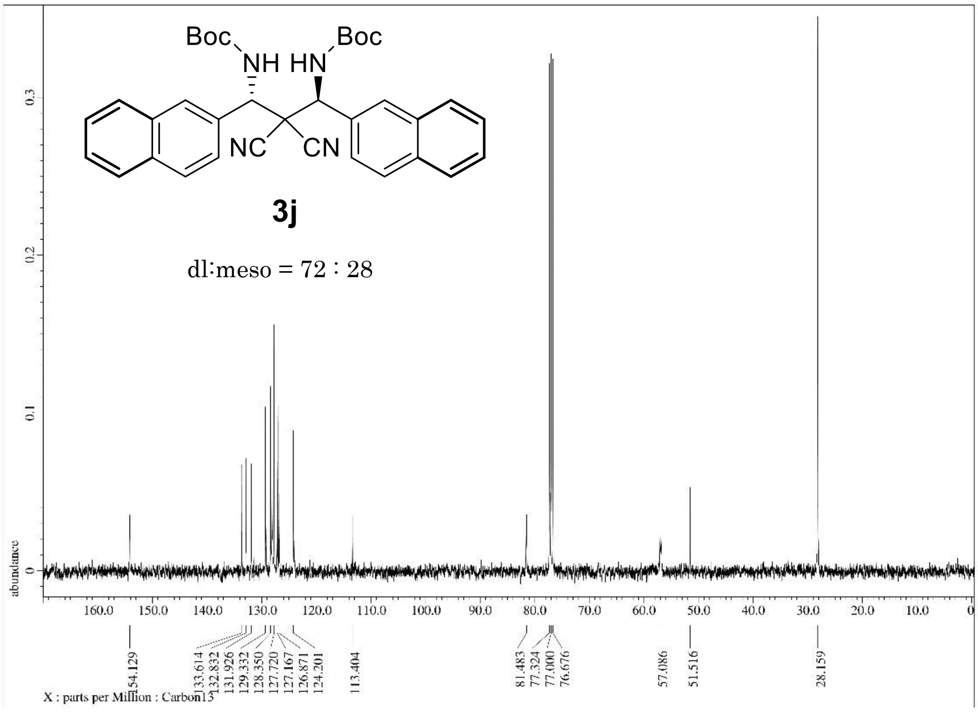
Boc
NH
HN
Boc
NC
CN
3j
dl:meso = 72 : 28
154.129
133.614
132.832
131.926
129.332
128.350
127.720
127.167
126.871
124.201
113.404
81.483
77.324
77.000
76.676
57.086
51.516
28.159
abundance
X : parts per Million : Carbon13

8. HPLC spectra

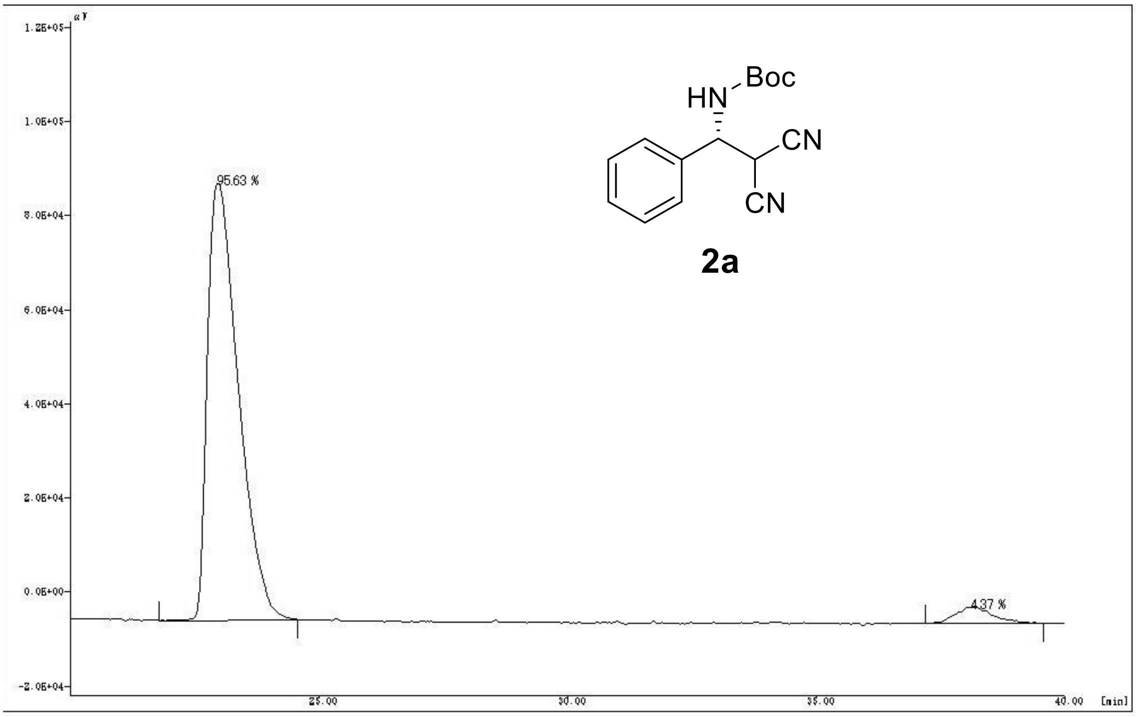

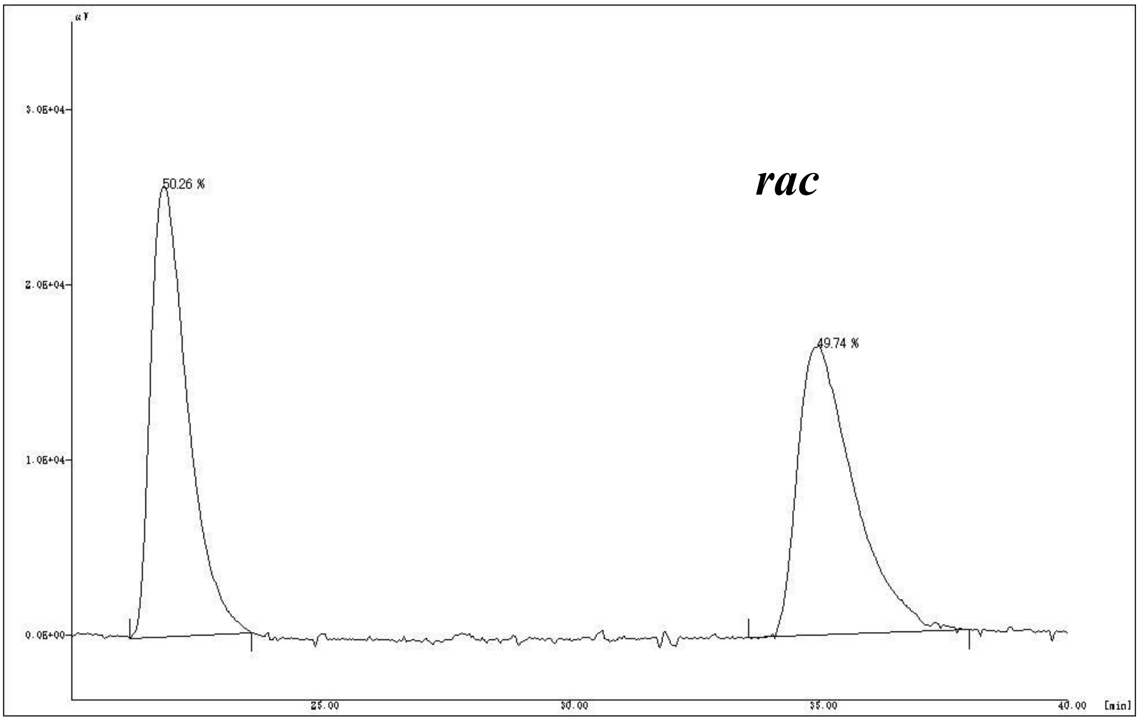

Chiralpak IA column (hexane:2-propanol = 90:10, 0.5 ml/min, 254nm)

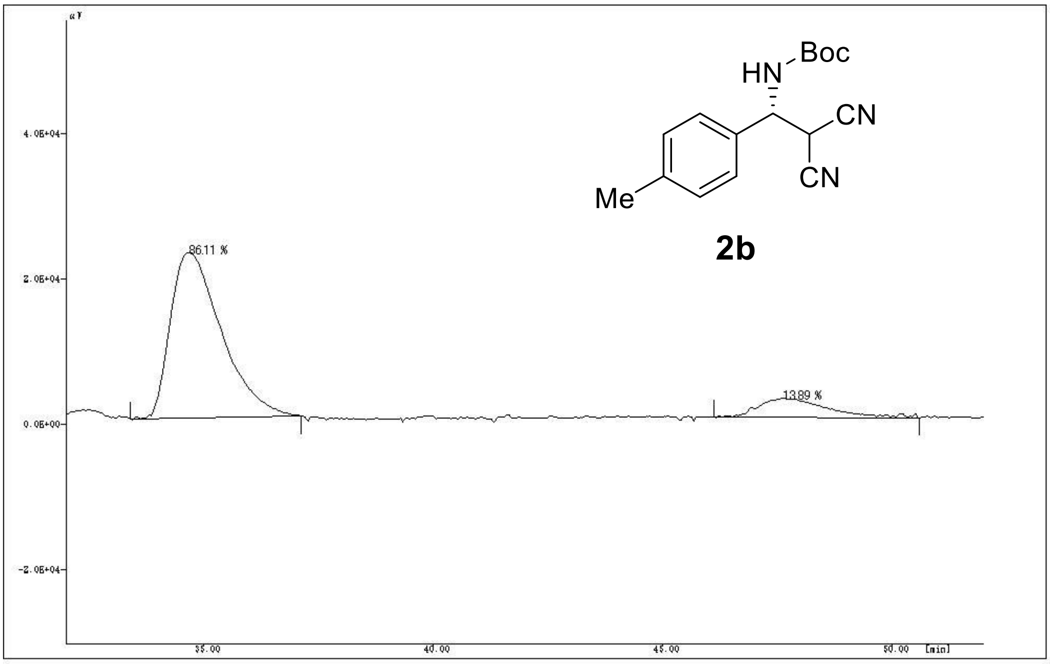

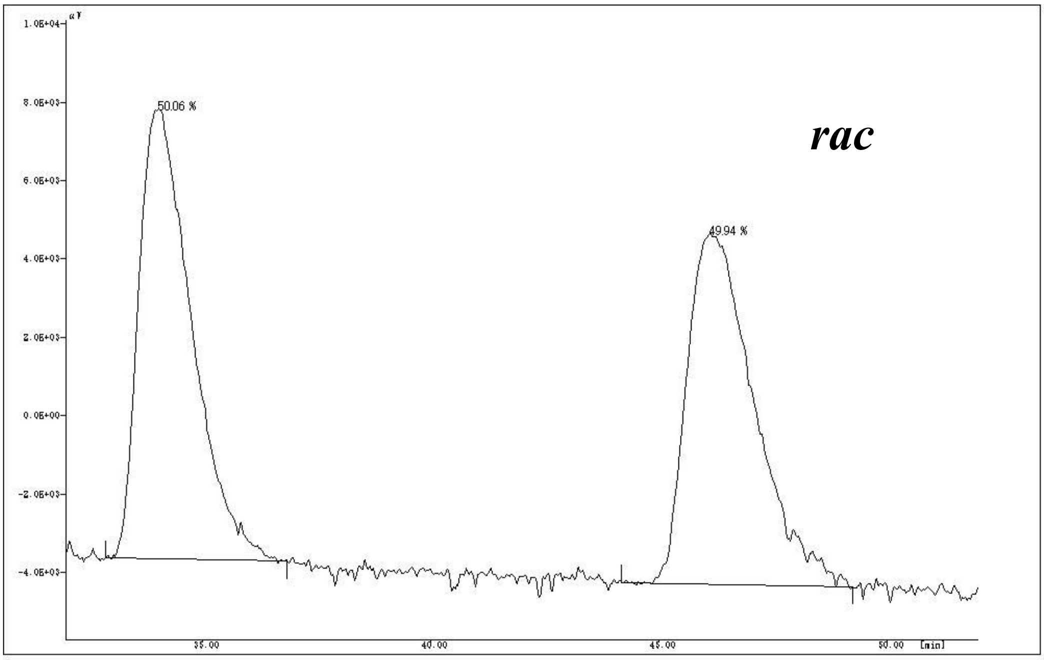

Chiralpak IA column (hexane:2-propanol = 90:10, 0.3 ml/min, 254 nm)

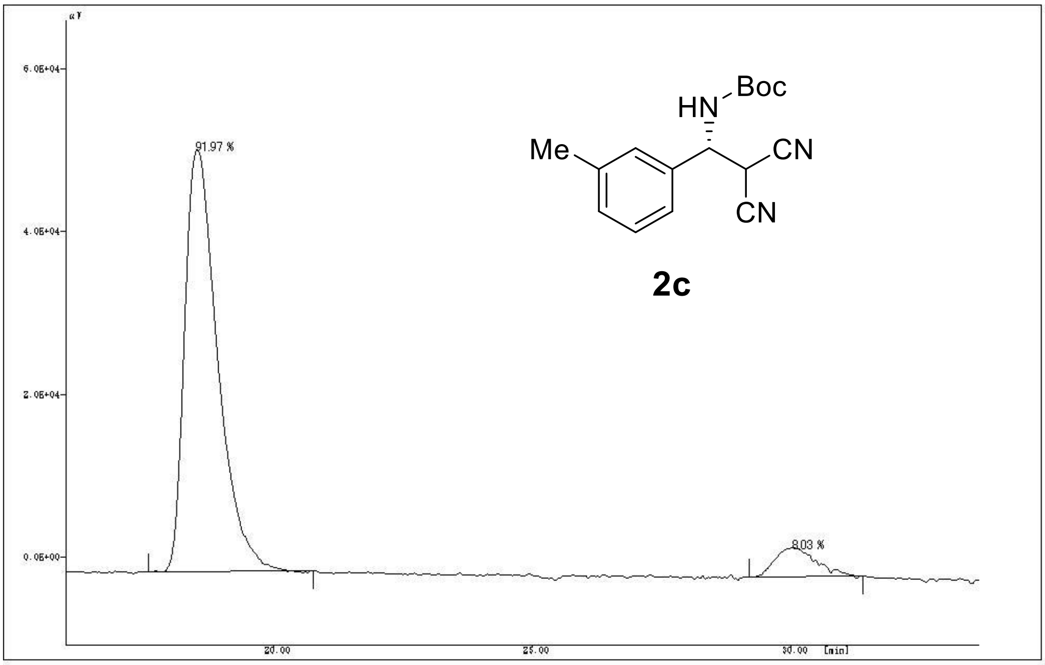

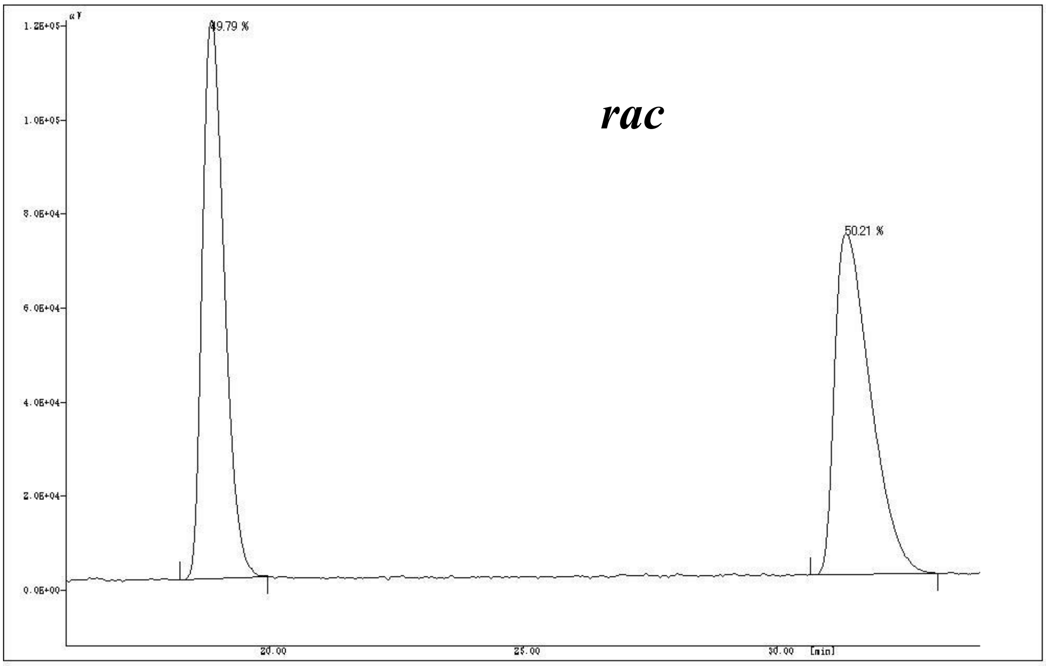

Chiralpak IA column (hexane:2-propanol = 90:10, 0.5 ml/min, 254 nm)

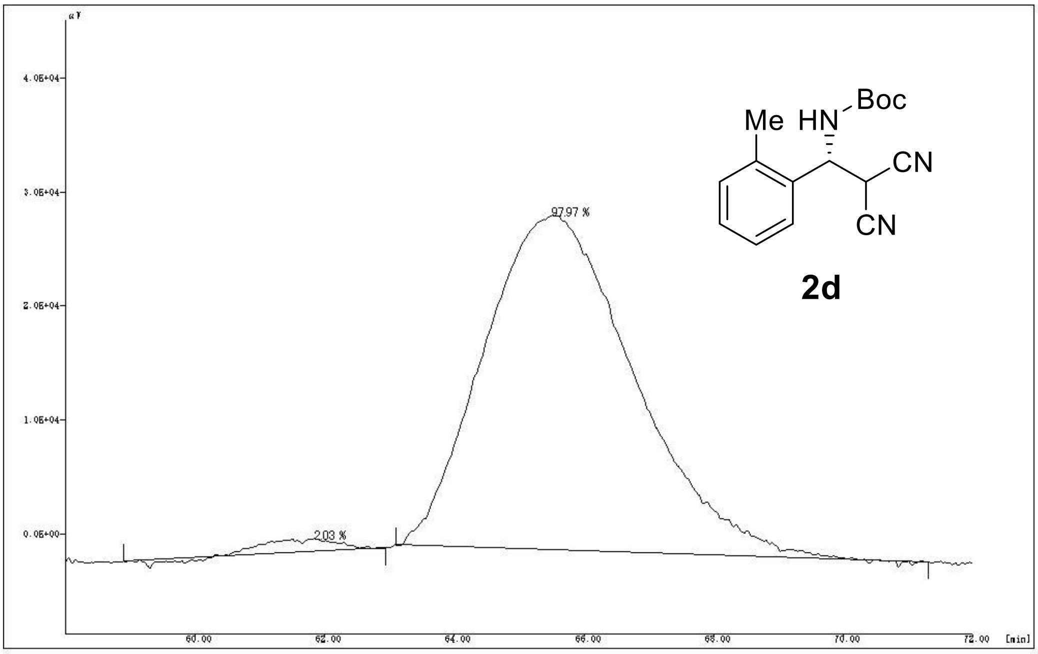

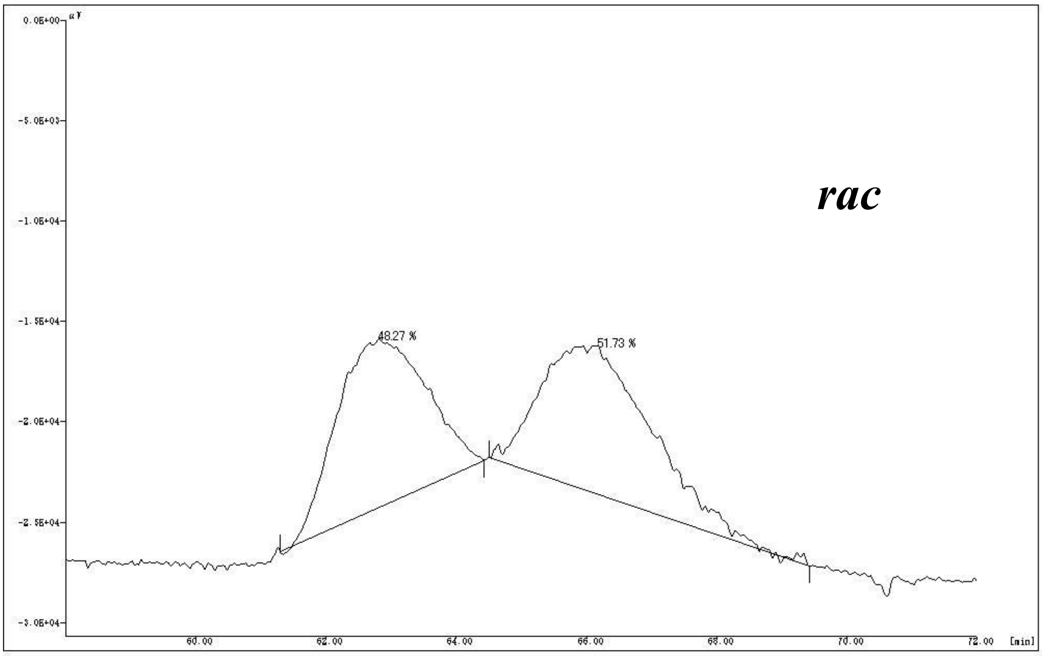

Chiralpak IA column (hexane:2-propanol = 95:5, 0.5 ml/min, 254 nm)

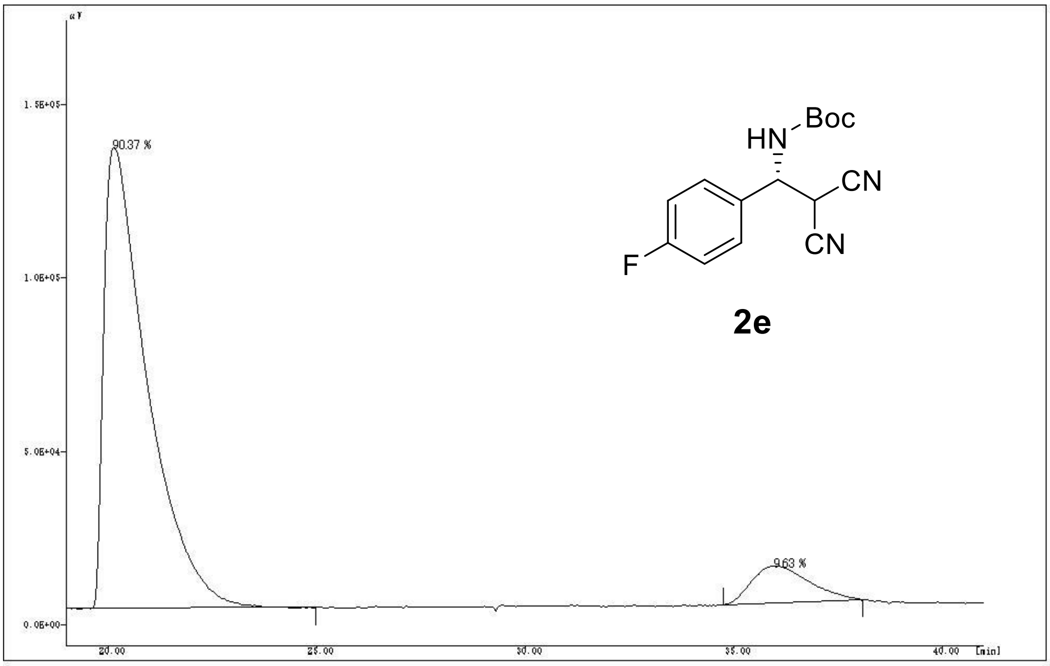

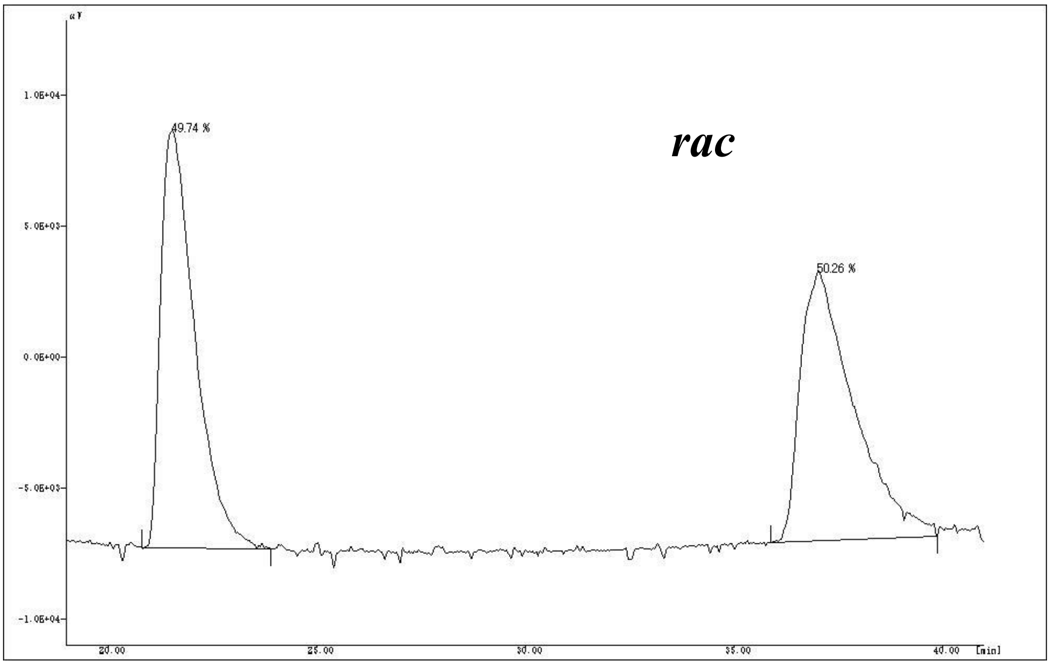

Chiralpak IA column (hexane:2-propanol = 90:10, 0.5 ml/min, 254 nm)

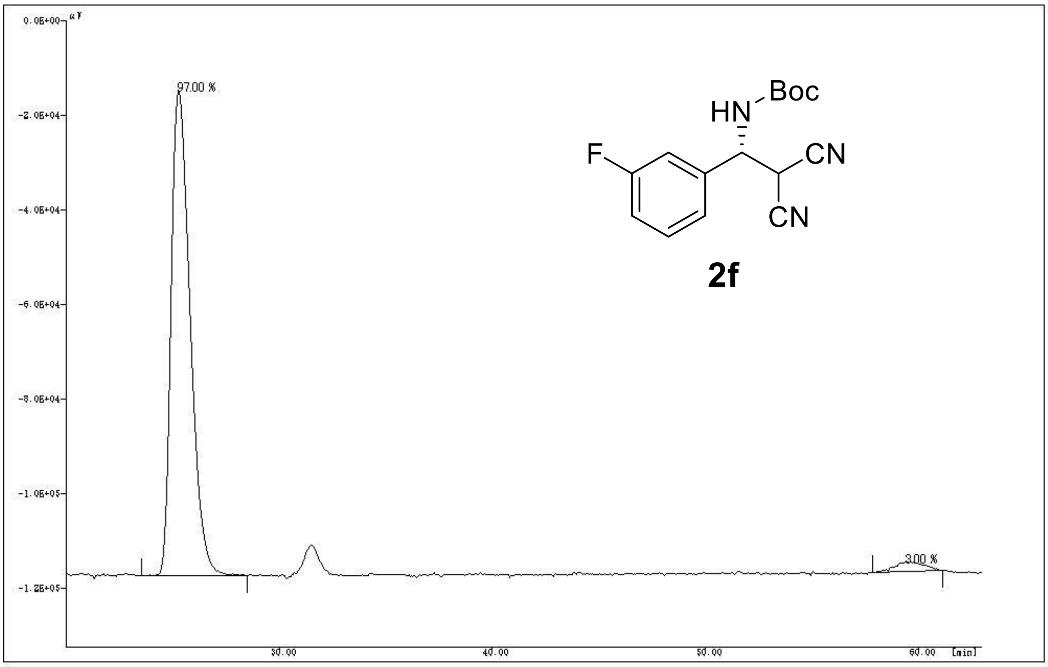

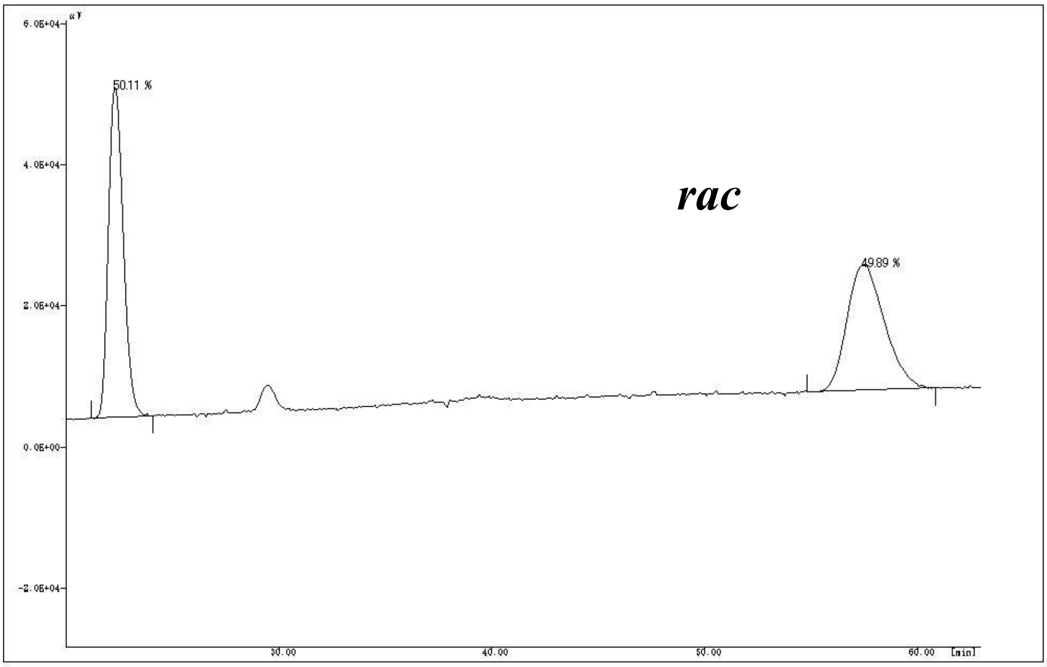

Chiralpak OD-H column (hexane:2-propanol = 90:10, 0.5 ml/min, 254 nm)

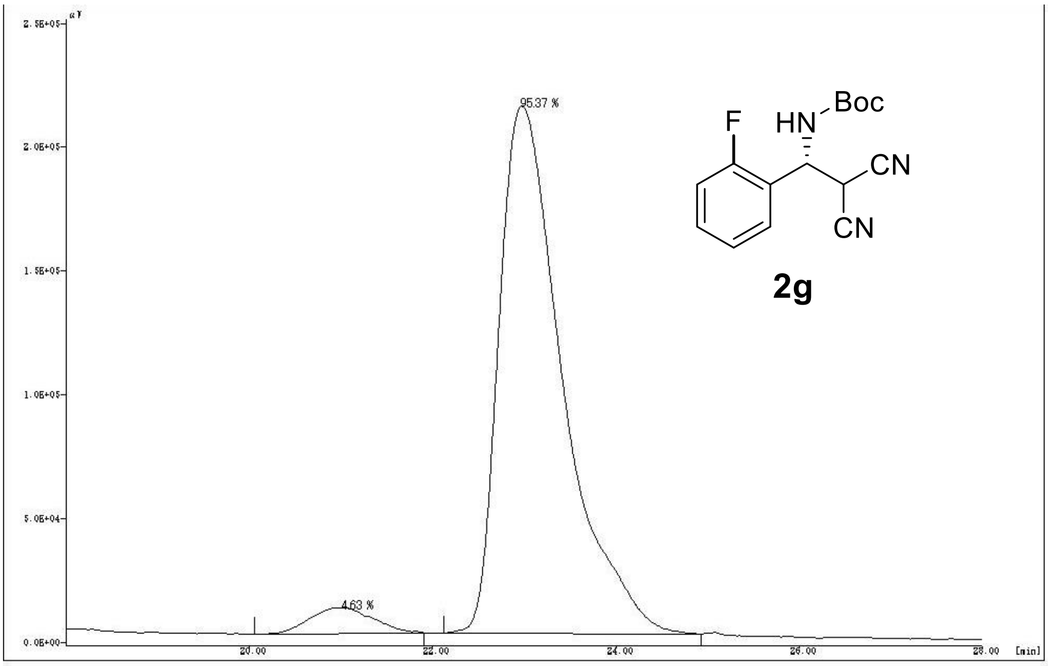

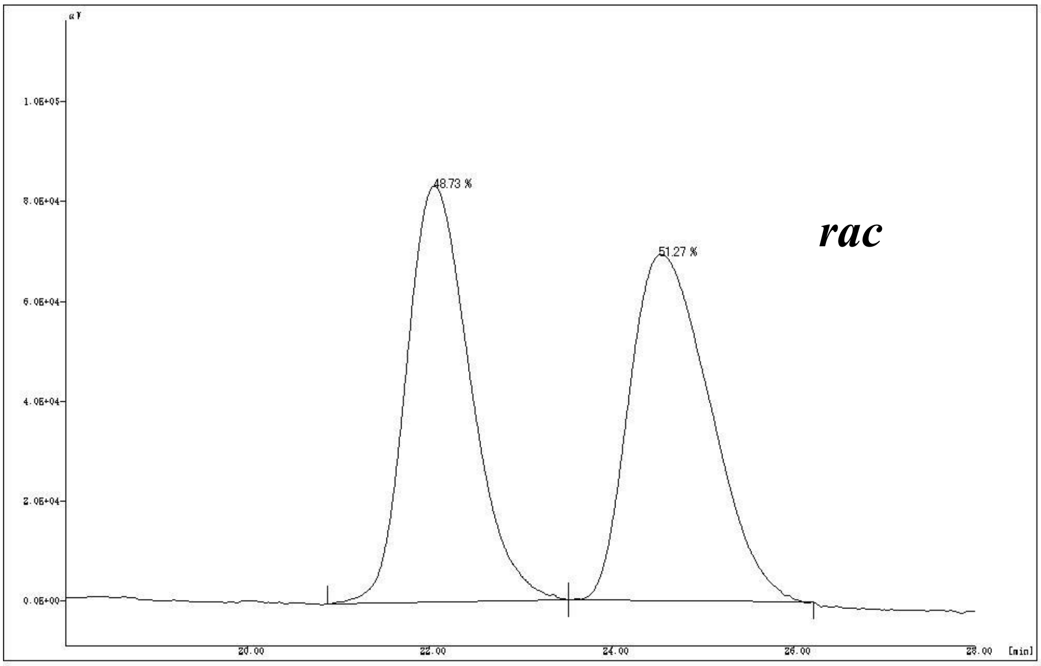

Chiralpak OD-H column (hexane:2-propanol = 75:25, 0.3 ml/min, 254 nm)

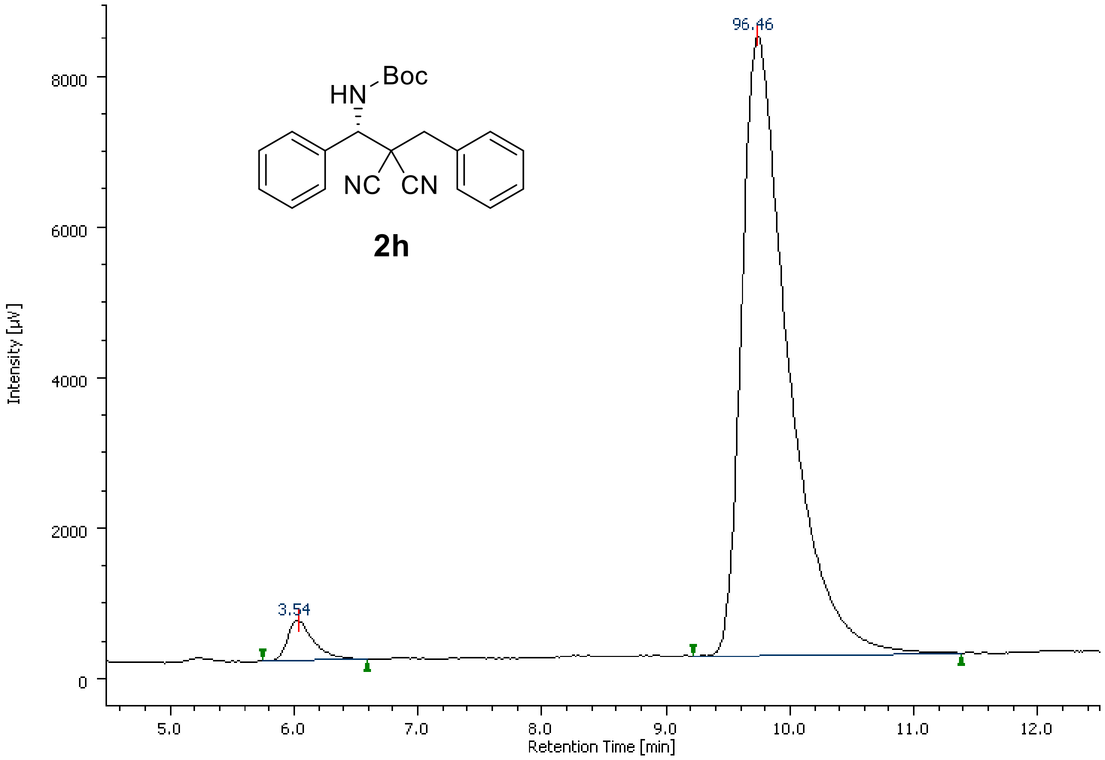

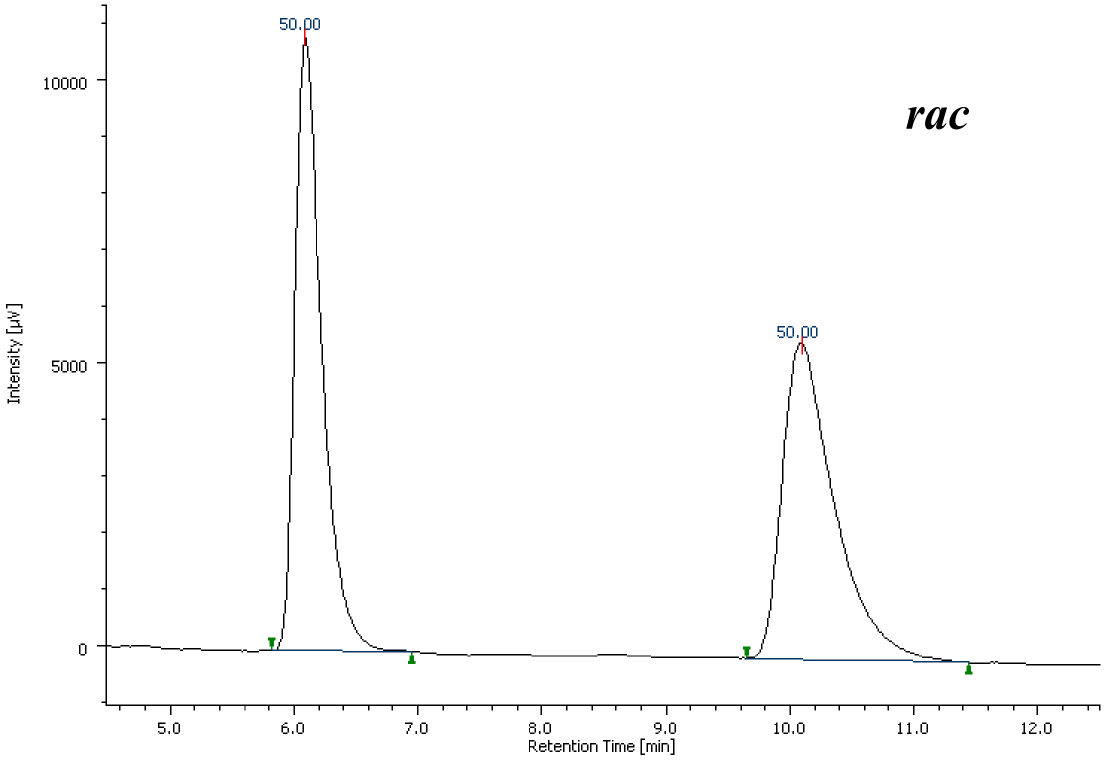

Chiralpak IA column (hexane:2-propanol = 80:20, 1.0 ml/min, 254 nm)

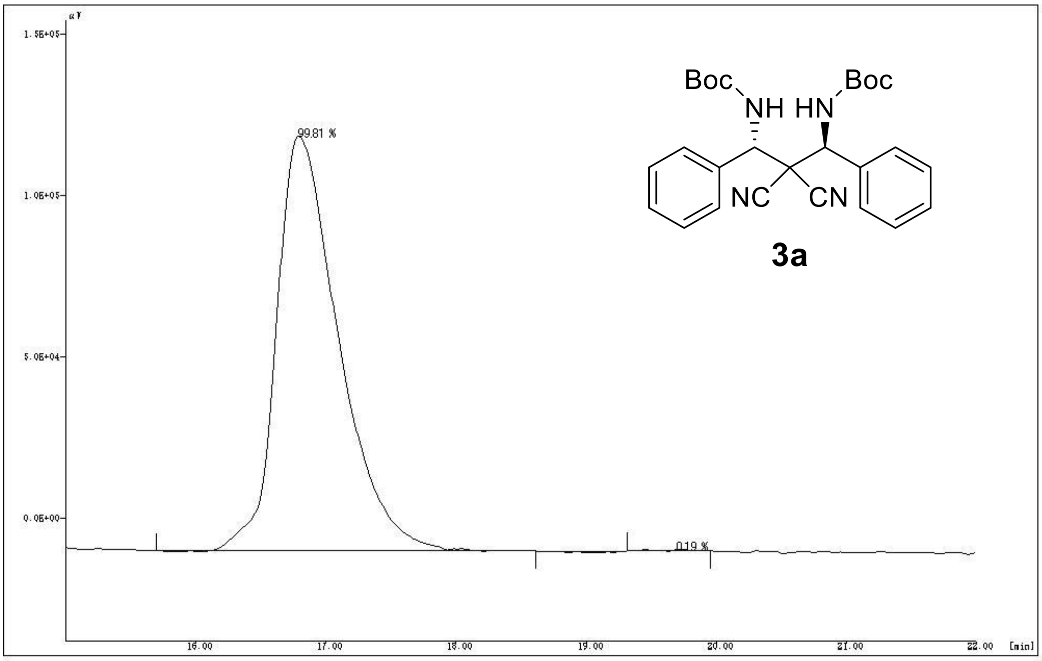

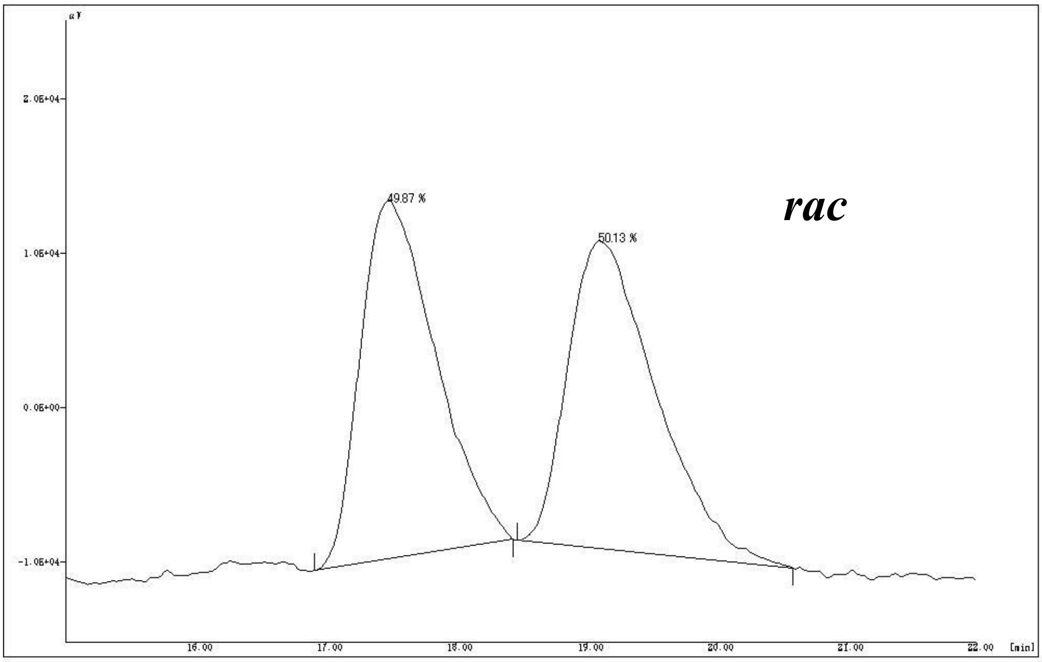

Chiralpak IA column (hexane:2-propanol = 90:10, 0.5 ml/min, 254 nm)

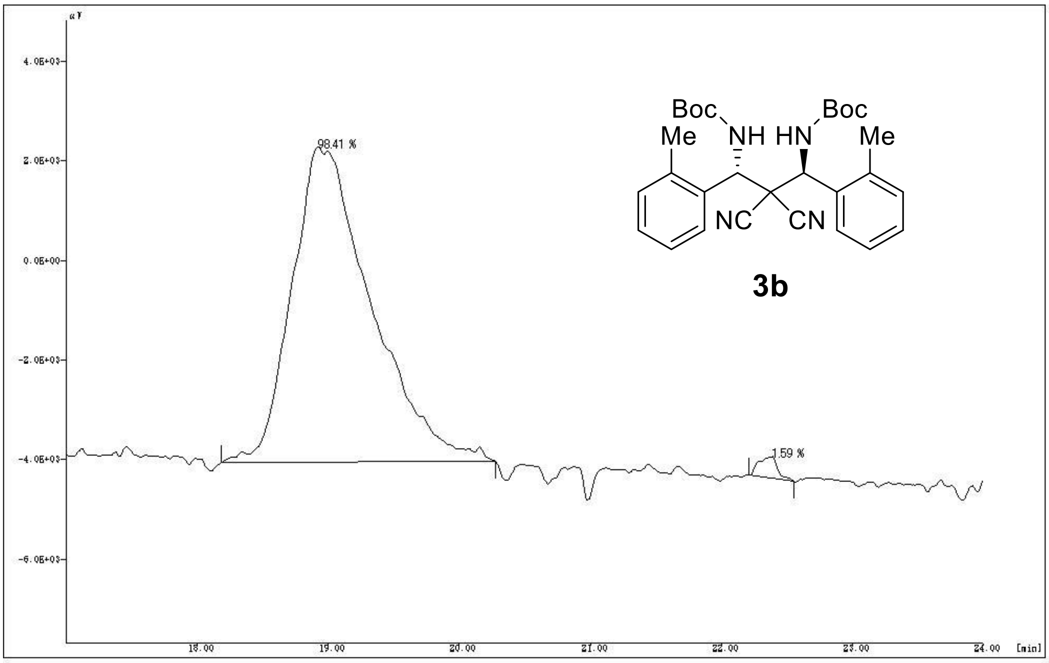

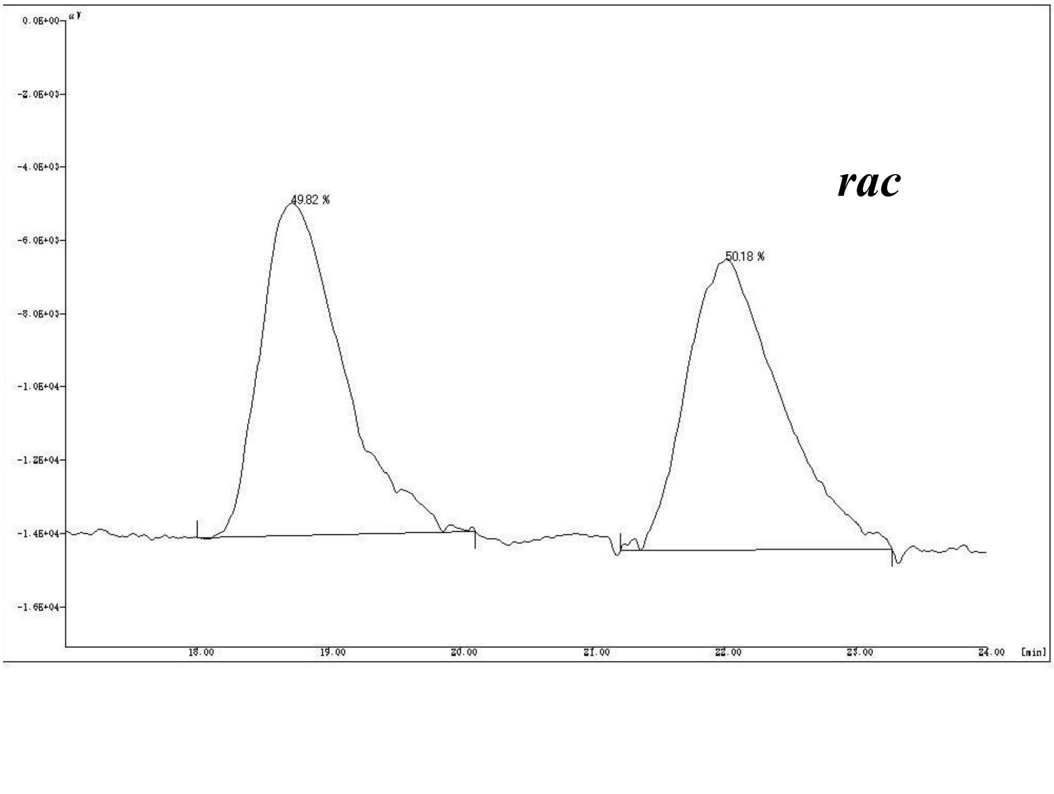

Chiralpak IA column (hexane:2-propanol = 90:10, 0.5 ml/min, 254 nm)

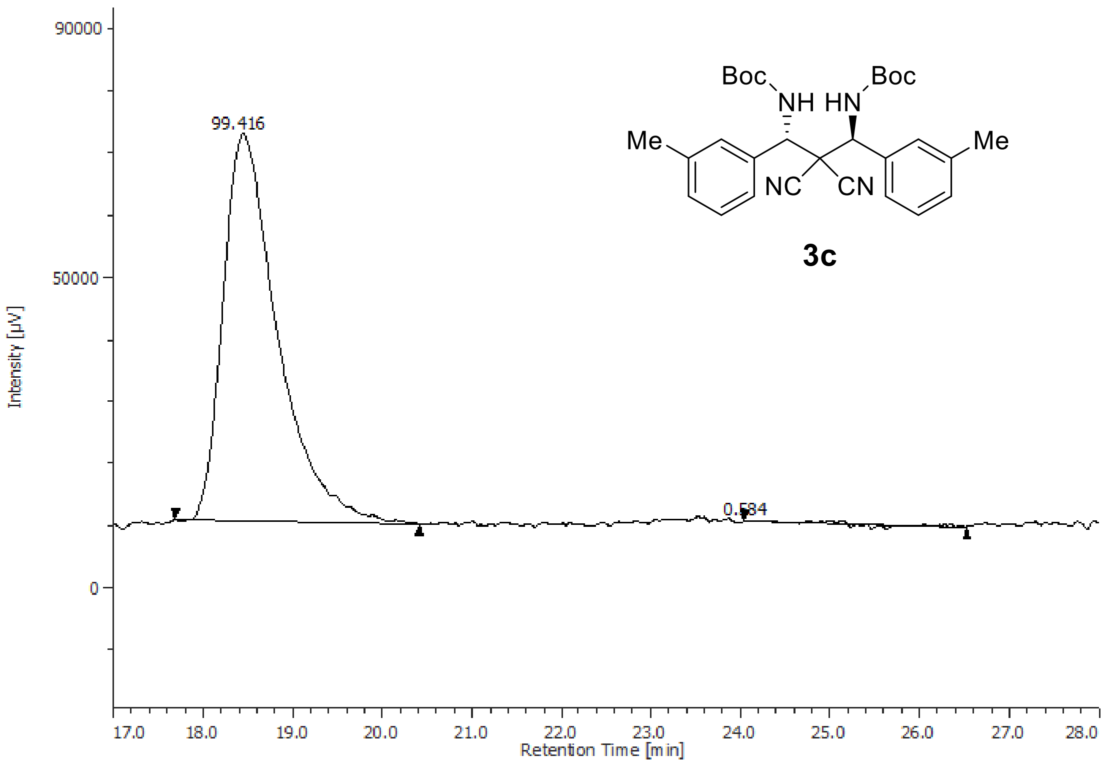

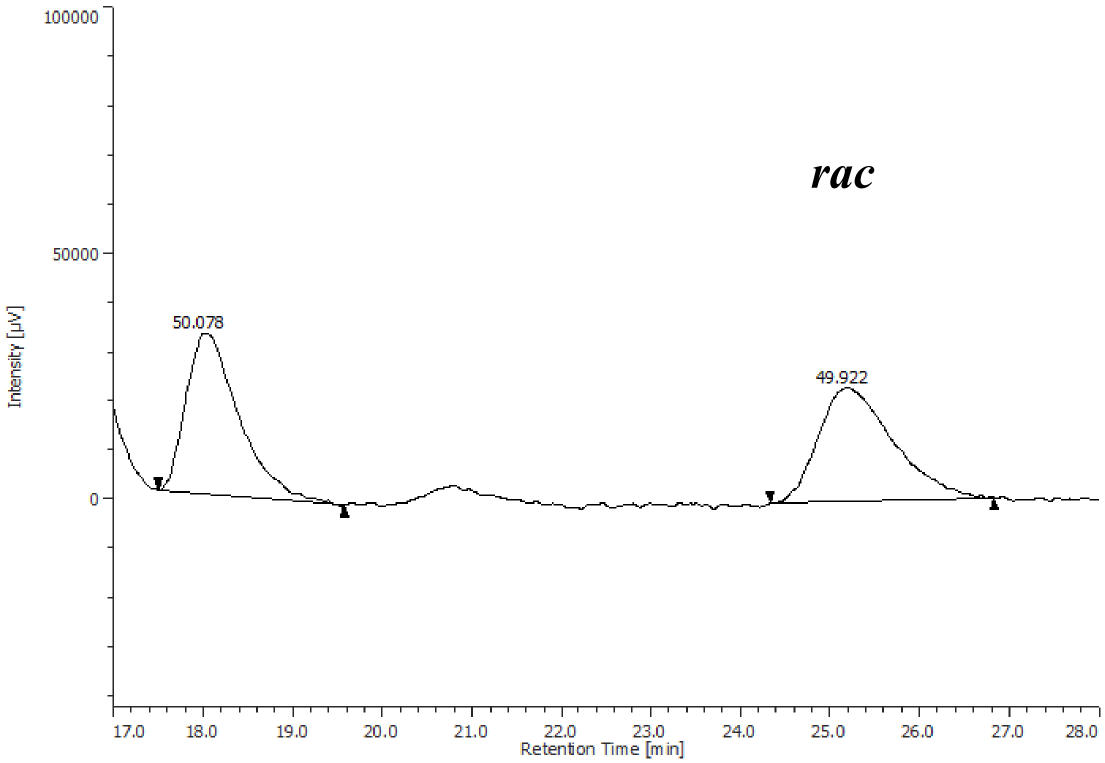

Chiralpak IA column (hexane:2-propanol = 95:5, 0.5 ml/min, 254 nm)

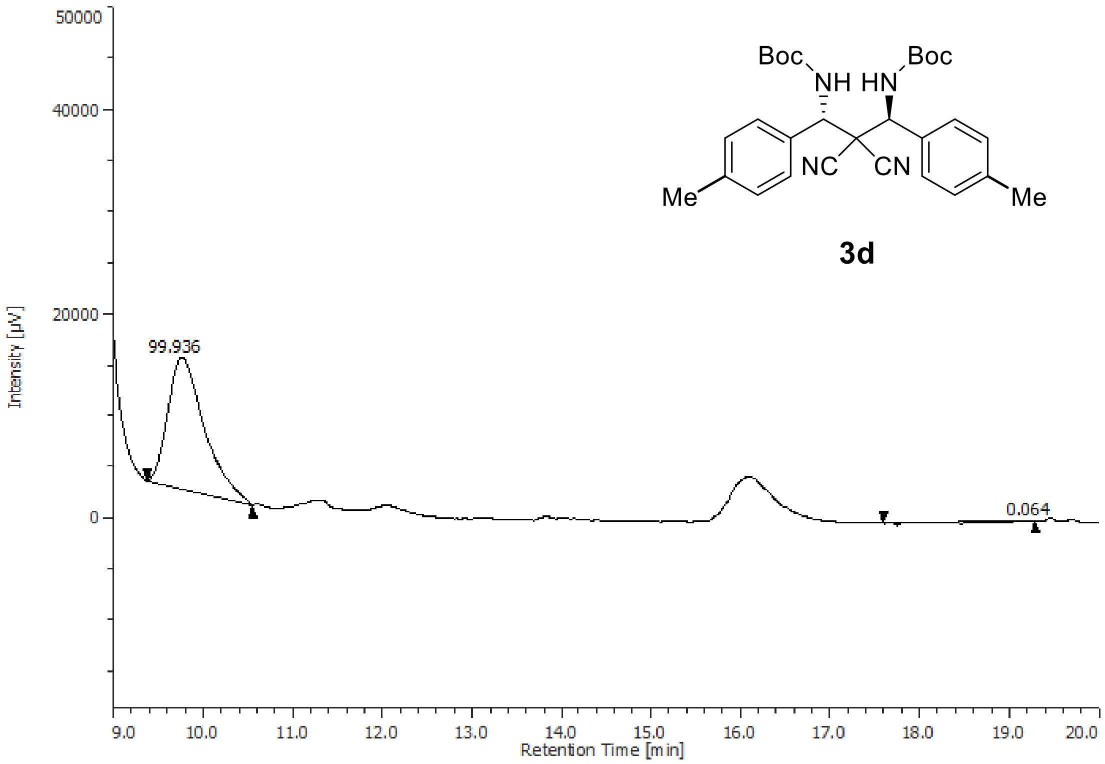

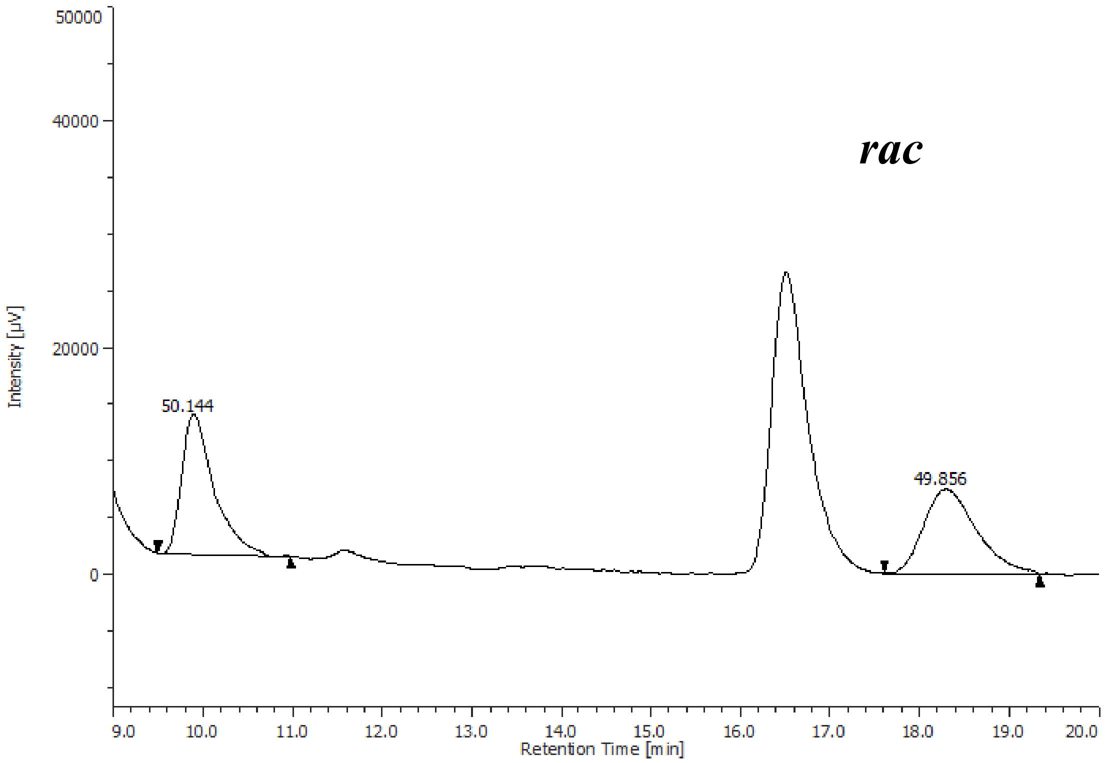

Chiralpak OD-H column (hexane:2-propanol = 70:30, 0.5 ml/min, 254 nm)

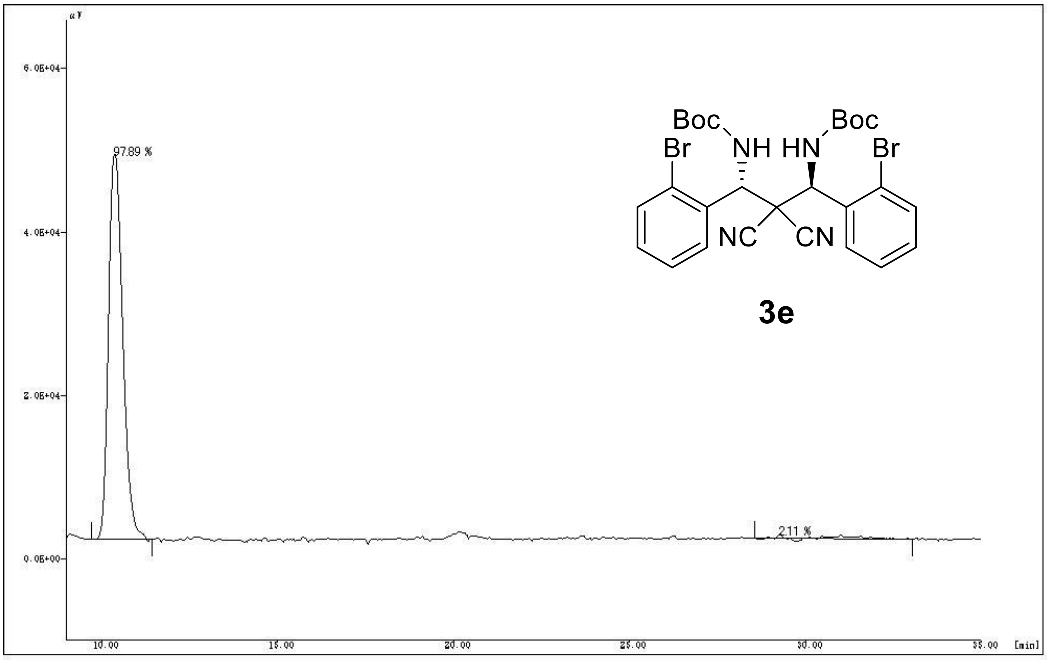

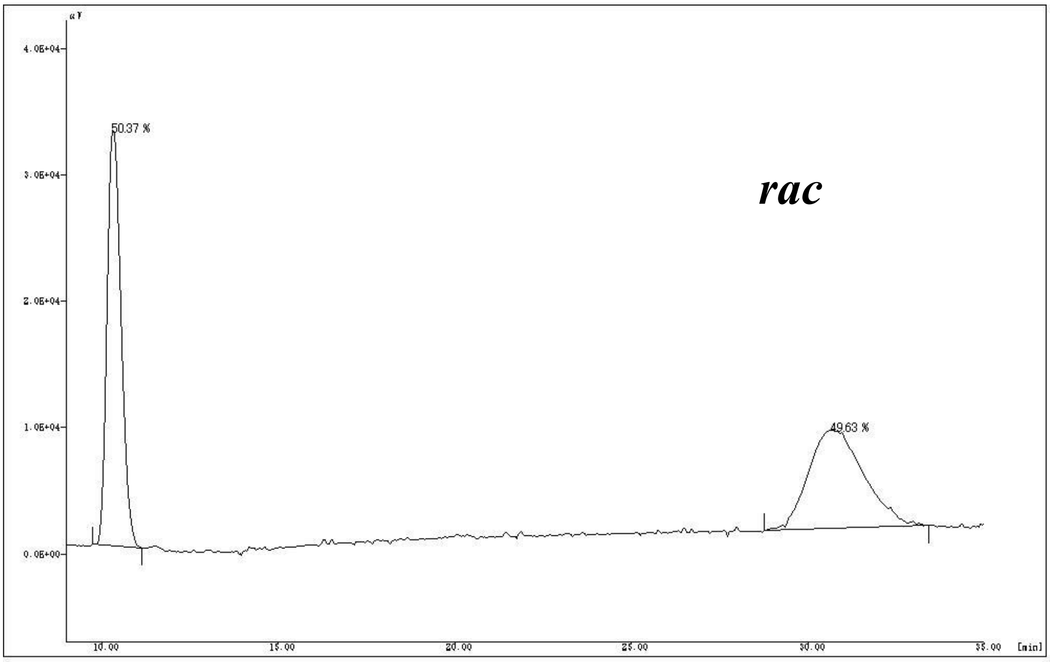

Chiralpak AD-H column (hexane:2-propanol = 90:10, 1.0 ml/min, 254 nm)

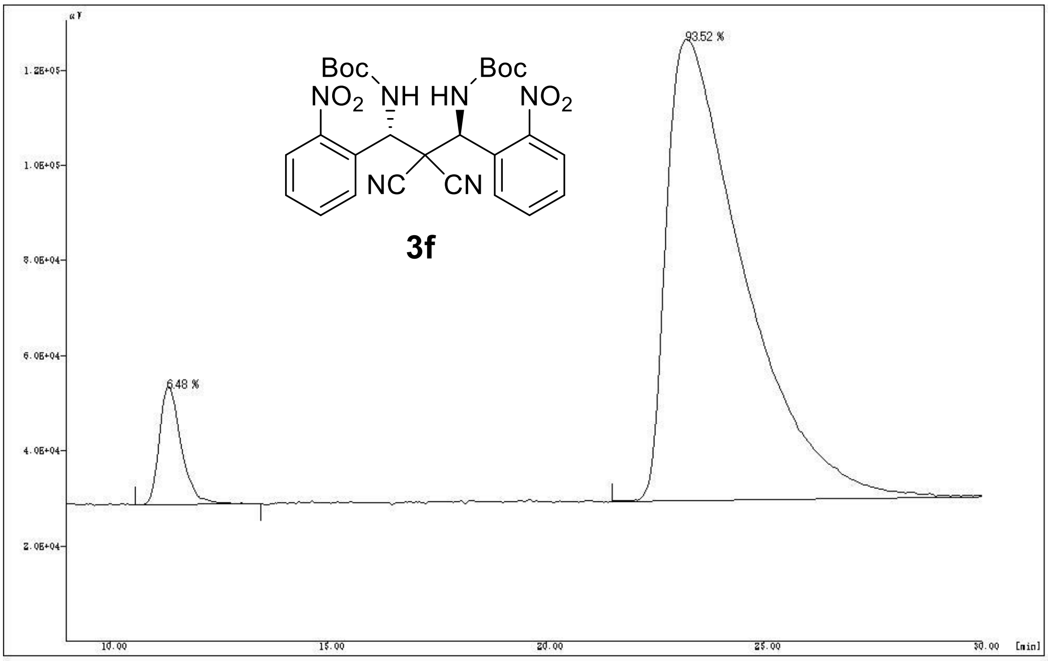

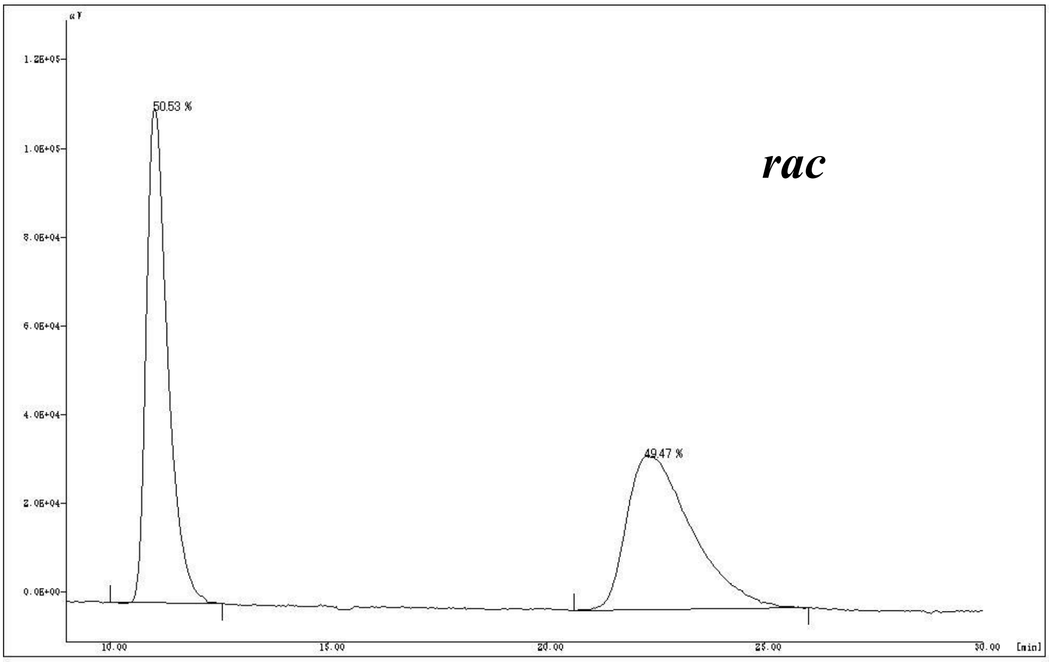

Chiralpak IA column (hexane:2-propanol = 70:30, 1.0 ml/min, 254 nm)

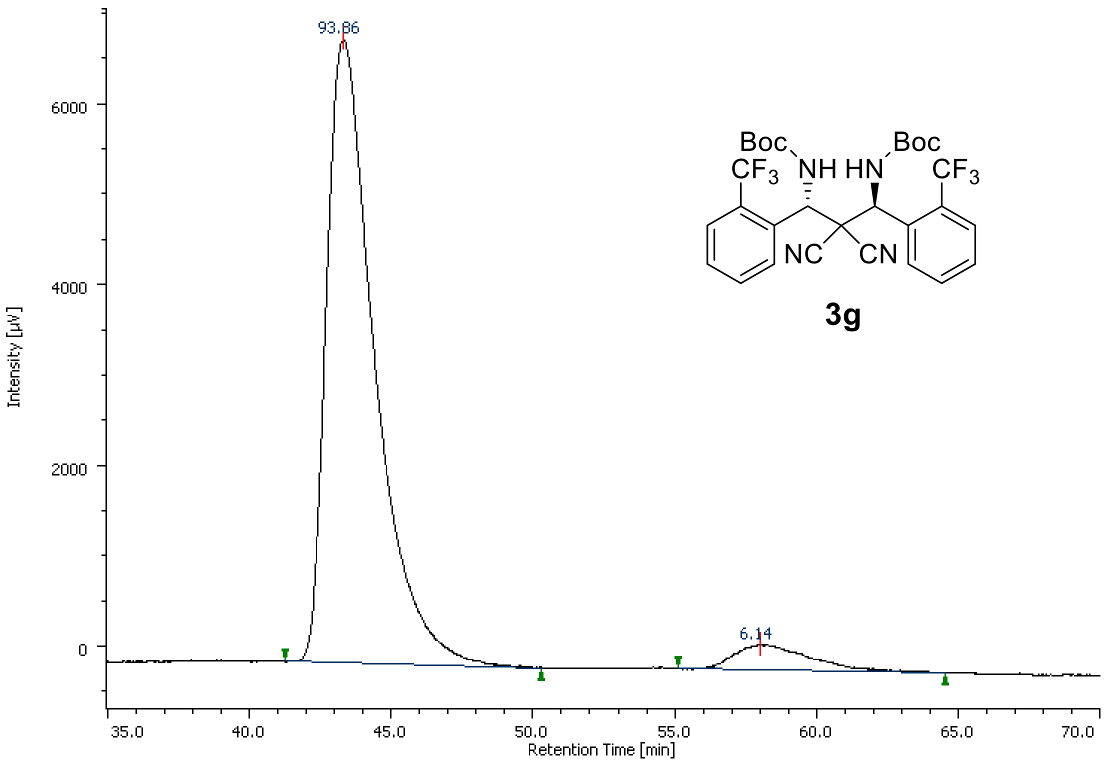

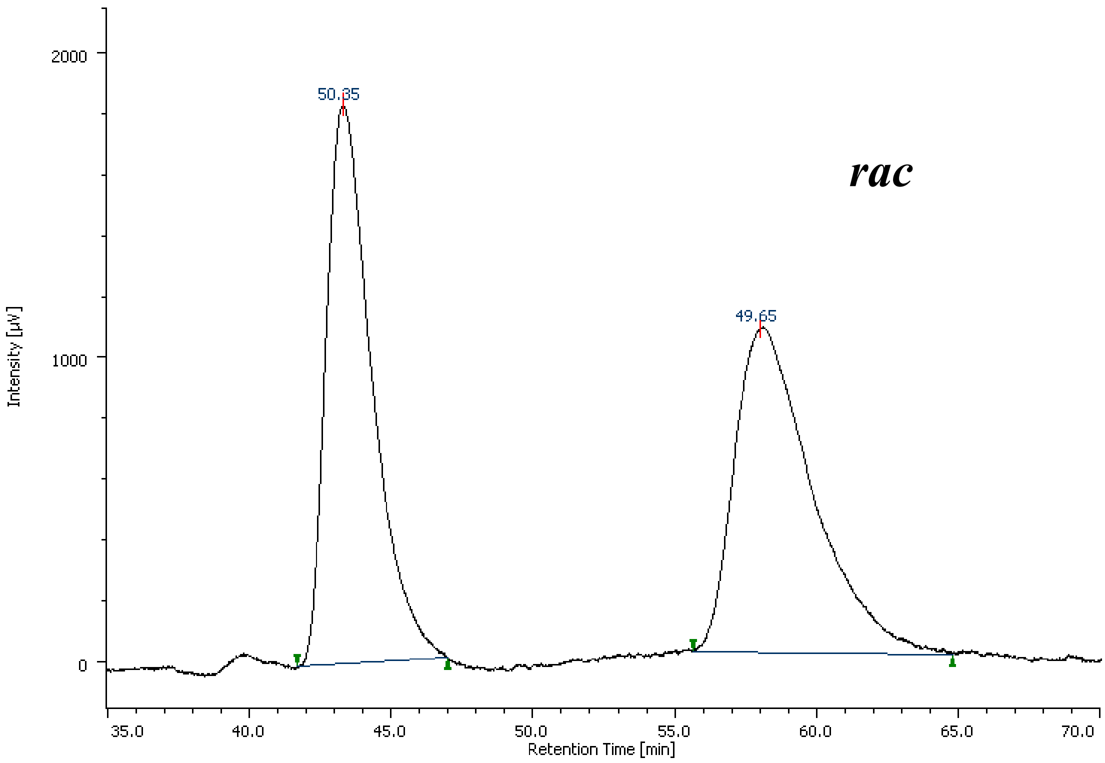

Chiralpak IA column (hexane:2-propanol = 95:5, 0.5 ml/min, 254 nm)

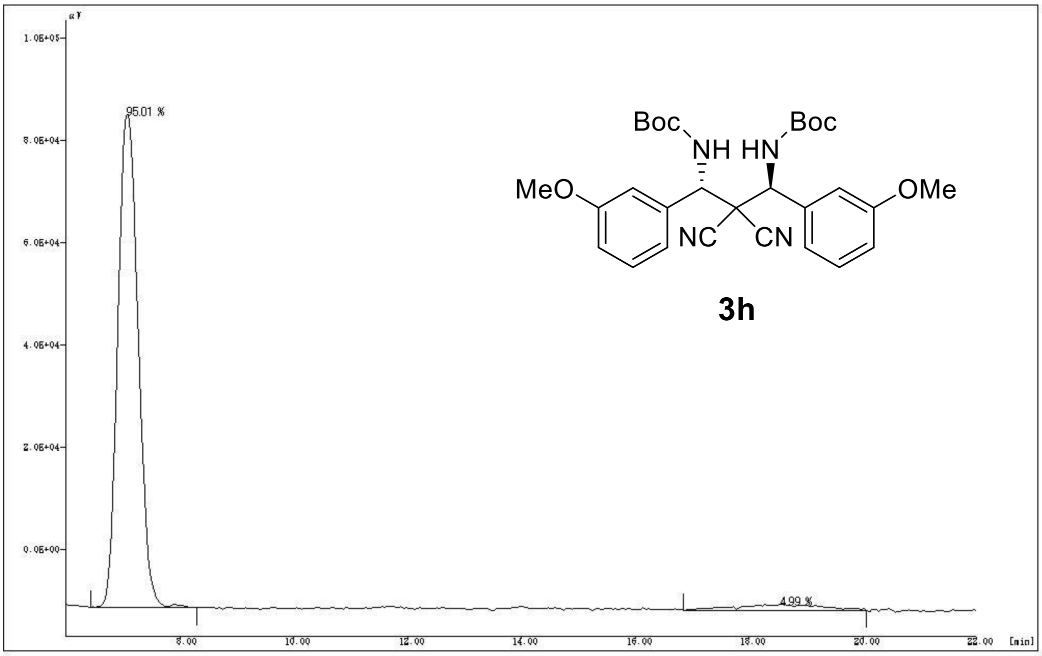

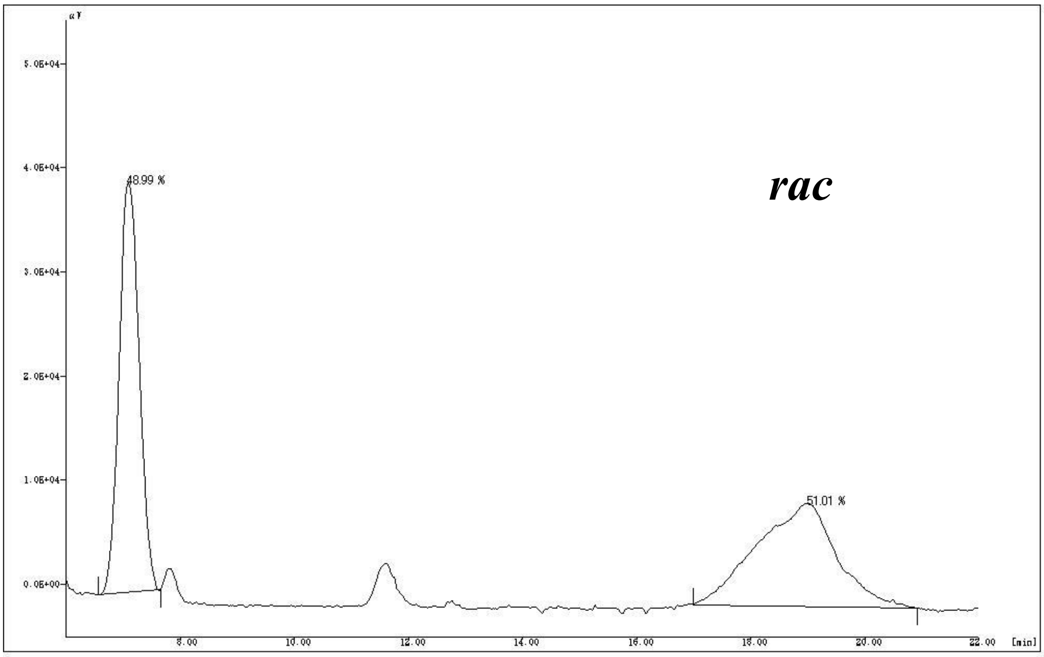

Chiralpak AD-H column (hexane:2-propanol = 85:15, 1.0 ml/min, 254 nm)

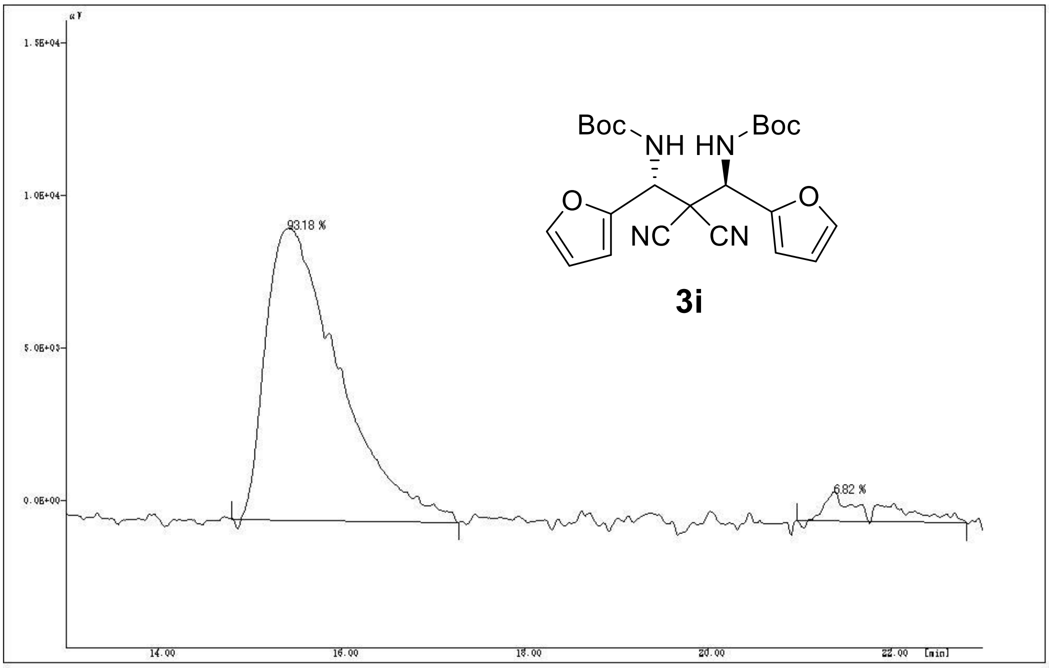

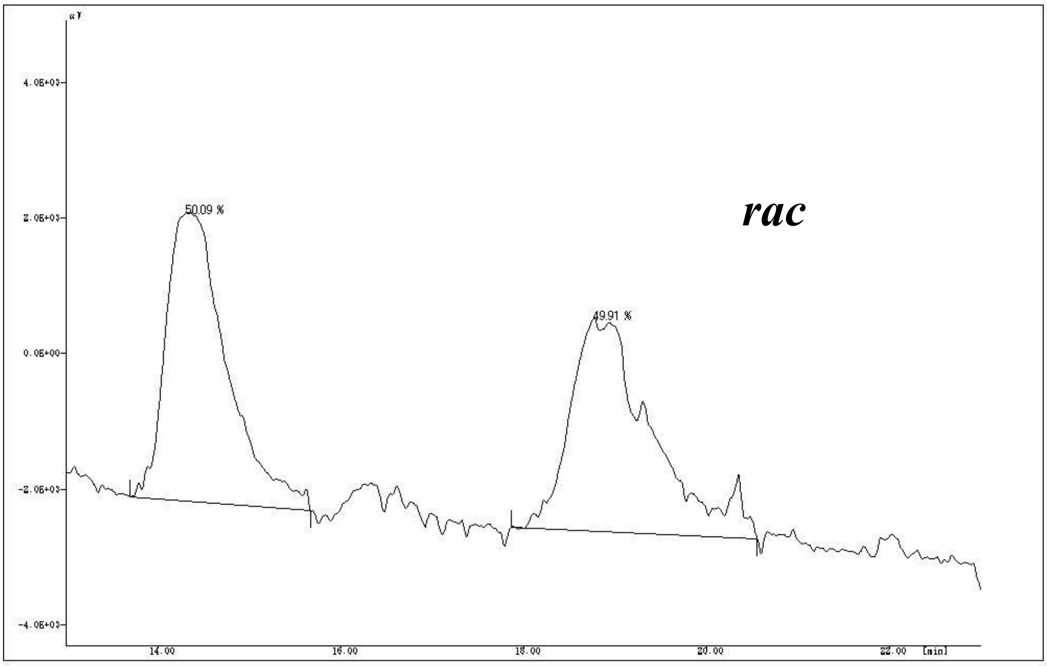

Chiralpak IA column (hexane:2-propanol = 90:10, 1.0 ml/min, 254 nm)

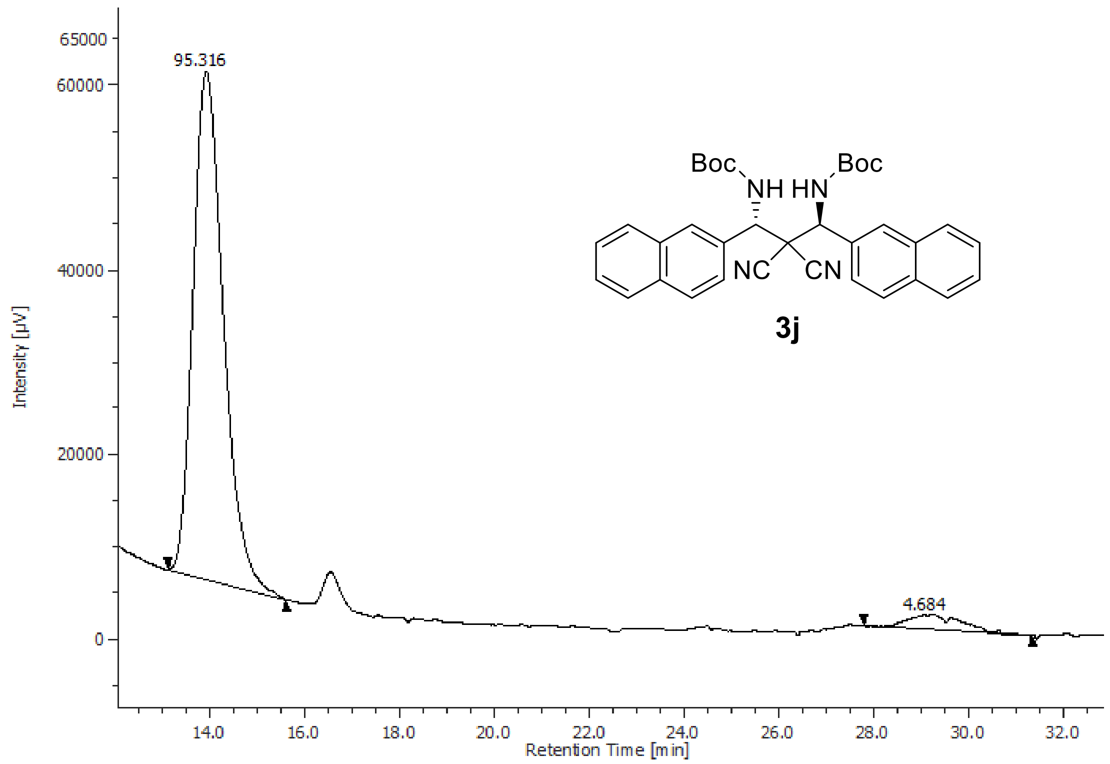

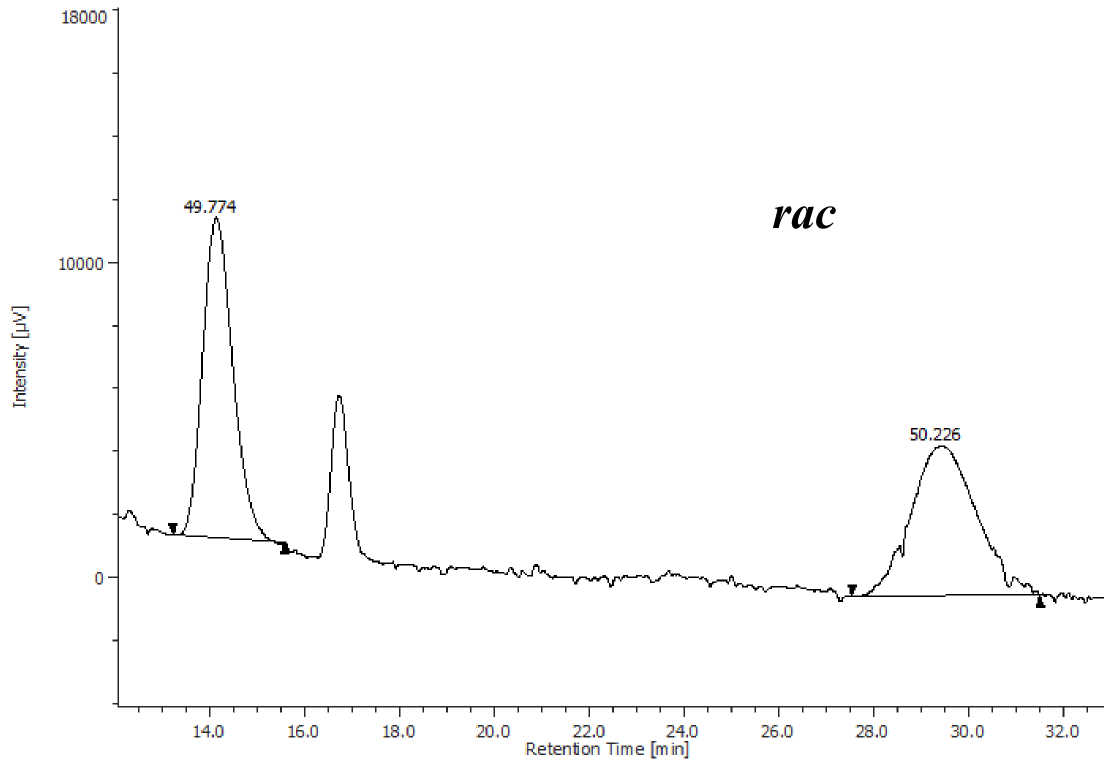

Chiralpak AS-H column (hexane:2-propanol = 90:10, 1.0 ml/min, 254 nm)

9. NMR and MS spectra of the interaction between catalyst and malononitrile

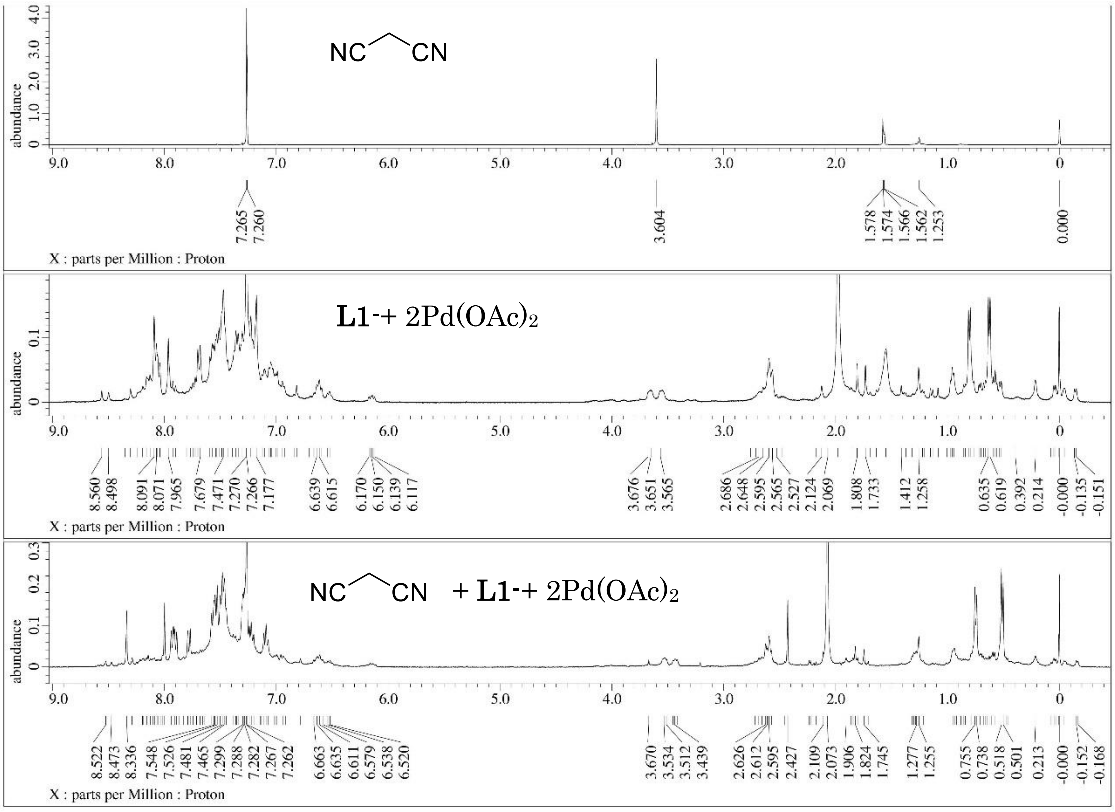

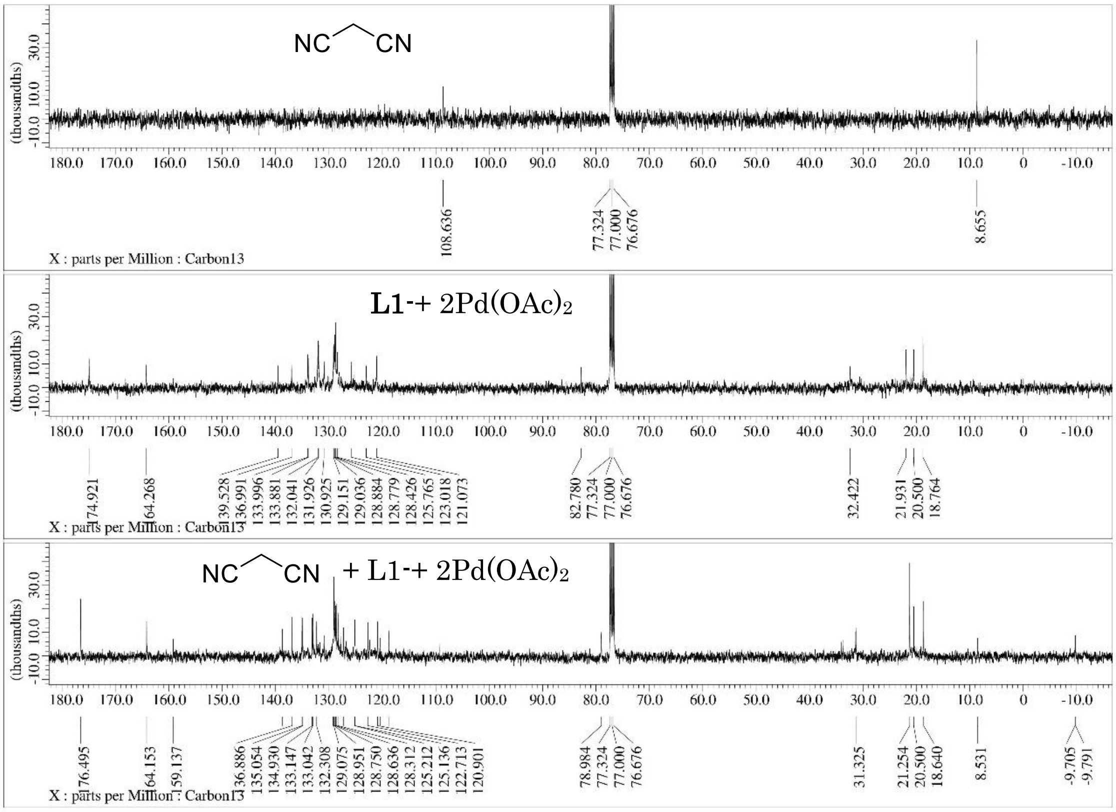

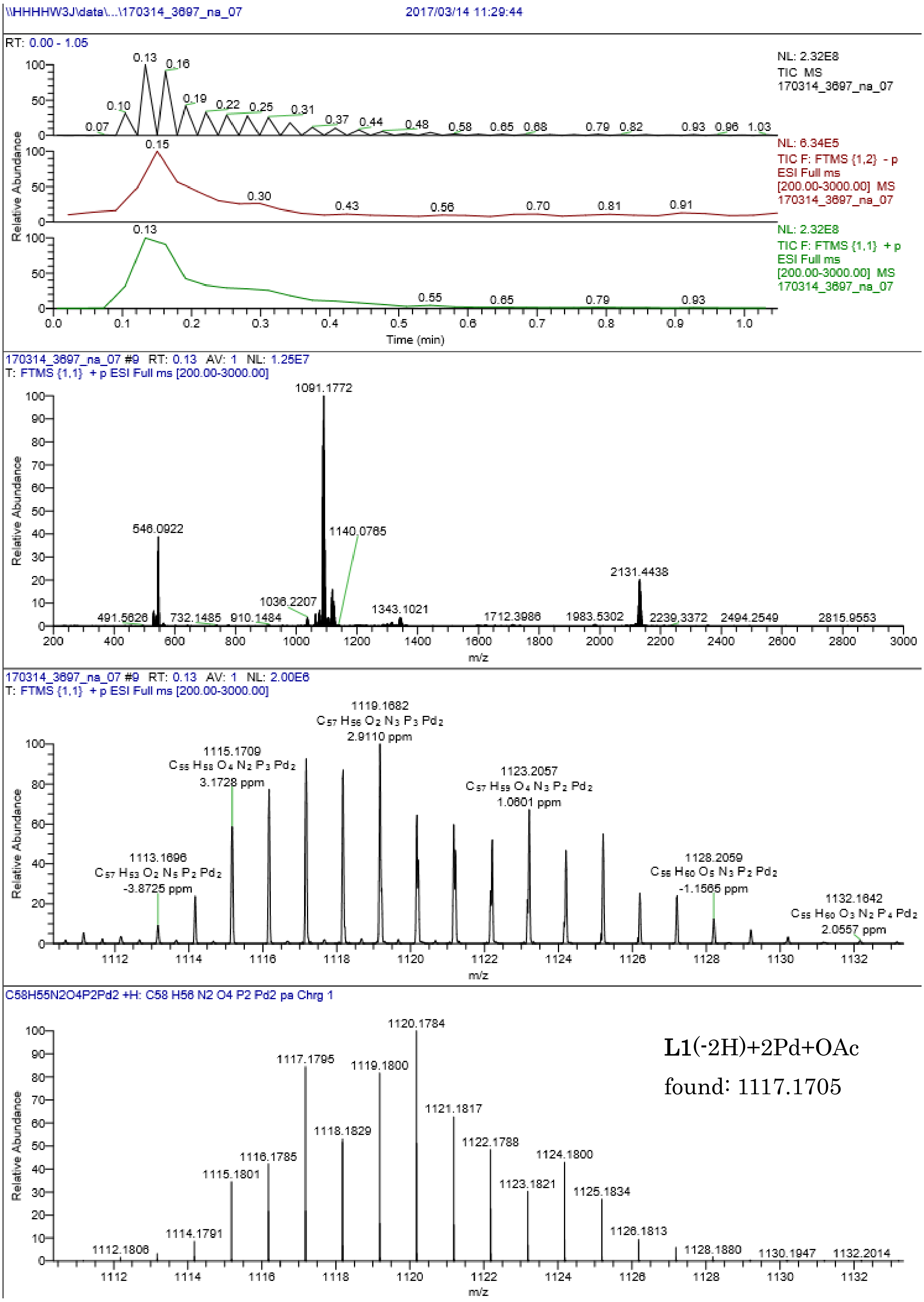

L1(-2H)+2Pd+OAc

found: 1117.1705

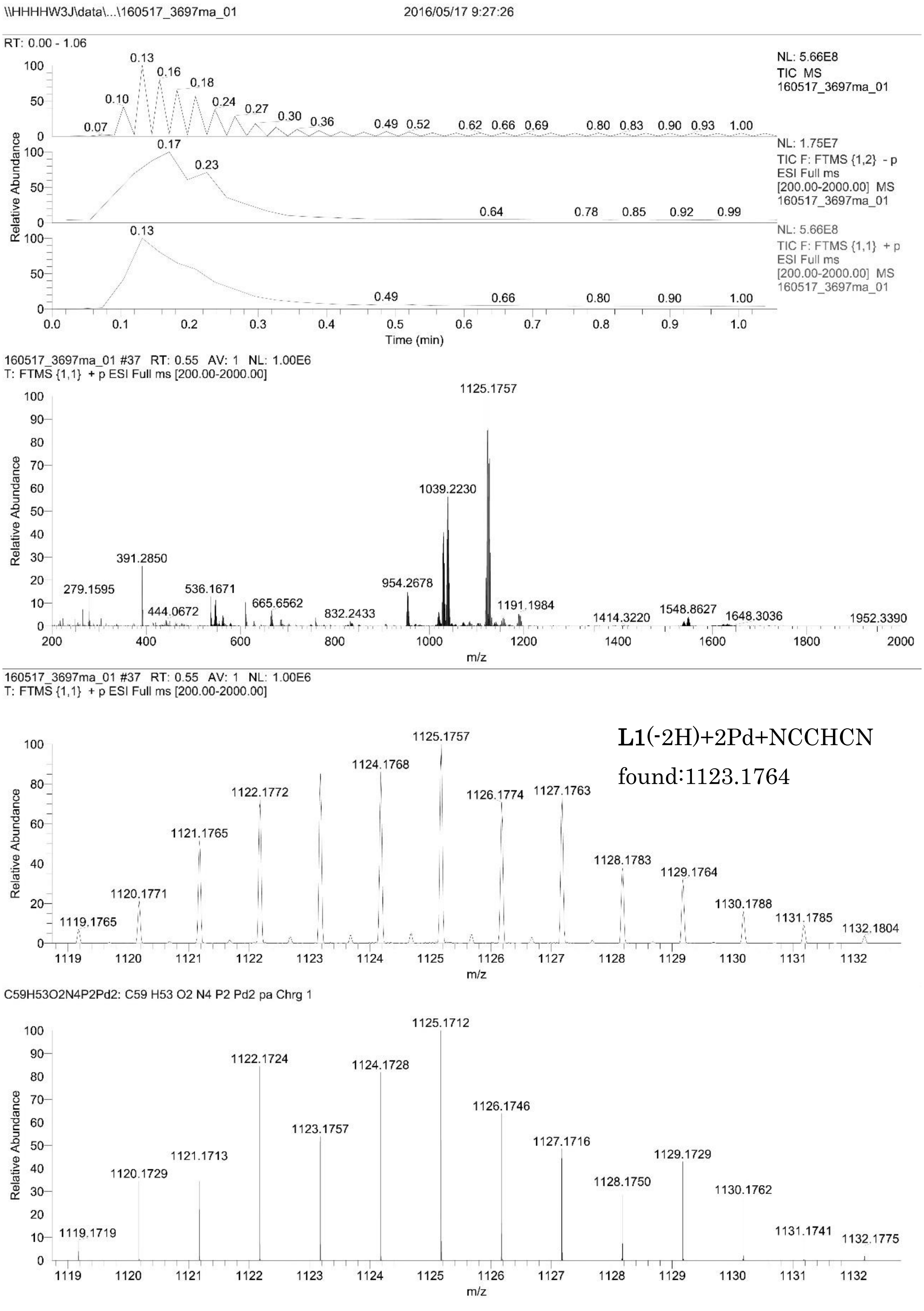
\\HHHHW3J\data\...\160517_3697ma_01
2016/05/17 9:27:26
RT: 0.00 - 1.06
NL: 5.66E8
TIC MS
160517_3697ma_01
NL: 1.75E7
TIC F: FTMS {1,2} - p ESI Full ms [200.00-2000.00] MS 160517_3697ma_01
NL: 5.66E8
TIC F: FTMS {1,1} + p ESI Full ms [200.00-2000.00] MS 160517_3697ma_01
Relative Abundance
Time (min)
160517_3697ma_01 #37 RT: 0.55 AV: 1 NL: 1.00E6
T: FTMS {1,1} + p ESI Full ms [200.00-2000.00]
1125.1757
1039.2230
954.2678
391.2850
279.1595
536.1671
444.0672
665.6562
832.2433
1191.1984
1414.3220
1548.8627
1648.3036
1952.3390
m/z
160517_3697ma_01 #37 RT: 0.55 AV: 1 NL: 1.00E6
T: FTMS {1,1} + p ESI Full ms [200.00-2000.00]
L1(-2H)+2Pd+NCCHCN
found:1123.1764
1119.1765
1120.1771
1121.1765
1122.1772
1124.1768
1125.1757
1126.1774
1127.1763
1128.1783
1129.1764
1130.1788
1131.1785
1132.1804
C59H53O2N4P2Pd2: C59 H53 O2 N4 P2 Pd2 pa Chrg 1
1119.1719
1120.1729
1121.1713
1122.1724
1123.1757
1124.1728
1125.1712
1126.1746
1127.1716
1128.1750
1129.1729
1130.1762
1131.1741
1132.1775
